# Supplementary material for: Child Health, Agriculture and Integrated Nutrition (CHAIN): protocol for a randomised controlled trial of improved infant and young child feeding in rural Zimbabwe
Source: BMJ Open. 2022 Dec 30;12(12):e056435. doi: 10.1136/bmjopen-2021-056435 (PMC9809274; doi:10.1136/bmjopen-2021-056435)
Supplement: Author's manuscript [file bmjopen-2021-056435.draft_revisions.pdf]

BMJ Open is committed to open peer review. As part of this commitment we make the peer review history of every article we publish publicly available.

When an article is published we post the peer reviewers' comments and the authors' responses online. We also post the versions of the paper that were used during peer review. These are the versions that the peer review comments apply to.

The versions of the paper that follow are the versions that were submitted during the peer review process. They are not the versions of record or the final published versions. They should not be cited or distributed as the published version of this manuscript.

BMJ Open is an open access journal and the full, final, typeset and author-corrected version of record of the manuscript is available on our site with no access controls, subscription charges or pay-per-view fees (http://bmjopen.bmj.com).

If you have any questions on BMJ Open's open peer review process please email info.bmjopen@bmj.com

BMJ Open

Child Health, Agriculture and Integrated Nutrition (CHAIN): protocol for a randomized controlled trial of improved infant and young child feeding in rural Zimbabwe

Journal:	*BMJ Open*
Manuscript ID	bmjopen-2021-056435
Article Type:	Protocol
Date Submitted by the Author:	23-Aug-2021

Complete List of Authors:	Smith, Laura ; Cornell University,<br>Chagwena, Dexter; Zvitambo Institute for Maternal and Child Health Research, Nutrition ; Ministry of Health and Child Care, Nutrition<br>Bourke, Claire ; Blizard Institute, Queen Mary University of London, UK<br>Robertson, Ruairi; Blizard Institute, Queen Mary University of London, UK,<br>Fernando, Shamiso; Zvitambo Institute for Maternal and Child Health Research, Nutrition<br>Tavengwa, Naume; Zvitambo Institute for Maternal and Child Health Research, Nutrition<br>Cairns, Jill; CIMMYT<br>Ndhlela, Thokozile; CIMMYT<br>Matumbu, Exhibit; Zvitambo Institute for Maternal and Child Health Research, Nutrition<br>Brown, Tim; Queen Mary University of London<br>Datta, Kavita; Queen Mary University of London<br>Mutasa, Batsirai; Zvitambo Institute for Maternal and Child Health Research, Nutrition<br>Tengende, Alice; Zvitambo Institute for Maternal and Child Health Research, Nutrition<br>Chidhanguro, Dzivaidzo; Zvitambo Institute for Maternal and Child Health Research, Nutrition<br>Langhaug, Lisa; Zvitambo Institute for Maternal and Child Health Research, Nutrition<br>Makanza, Maggie; Zvitambo Institute for Maternal and Child Health Research, Nutrition<br>Chasekwa, Bernard; Zvitambo Institute for Maternal and Child Health Research, Nutrition<br>Mutasa, Kuda; Zvitambo Institute for Maternal and Child Health Research, Harare, Zimbabwe<br>Swann, Jonathan; University of Southampton Faculty of Medicine<br>Kelly, Paul; Barts and The London School of Medicine<br>Ntozini, Robert; Zvitambo Institute, Biostatistics & IT<br>Prendergast, Andrew ; Queen Mary University of London,

Keywords:	Nutrition < TROPICAL MEDICINE, Immunology < THORACIC MEDICINE, QUALITATIVE RESEARCH, Community child health < PAEDIATRICS, Nutritional support < GASTROENTEROLOGY

SCHOLARONE™
Manuscripts

Child Health, Agriculture and Integrated Nutrition (CHAIN): protocol for a randomized controlled trial of improved infant and young child feeding in rural Zimbabwe

Laura E Smith[1,2], Dexter T. Chagwena[1, 3], Claire D. Bourke[4], Ruairi C. Robertson[4], Shamiso Fernando[1], Naume V. Tavengwa[1], Jill Cairns[5], Thokozile Ndhlela[5], Exhibit Matumbu[1], Tim Brown[7], Kavita Datta[7], Batsirai Mutasa[1], Alice Tengende[1], Dzivaidzo Chidhanguro[1], Lisa Langhaug[1], Maggie Makanza[1], Bernard Chasekwa[1], Kuda Mutasa[1], Jonathan Swann[6], Paul Kelly[4], Robert Ntozini[1], Andrew J. Prendergast[1,4]

[1]Zvitambo Institute for Maternal and Child Health Research, Harare, Zimbabwe

[2]Department of Population Medicine and Diagnostics, Cornell University, Ithaca, NY, USA

[3]Ministry of Health and Child Care, Harare, Zimbabwe

[4]Blizard Institute, Queen Mary University of London, UK

[5]CIMMYT, Harare, Zimbabwe

[6]University of Southampton, UK

[7]School of Geography, Queen Mary University of London, UK

For peer review only

Short title: CHAIN Trial Protocol

Word count: 5835

Abstract: 395

Corresponding author

Dexter Chagwena, Zvitambo Institute for Maternal and Child Health Research, 16 Lauchlan Ave, Meyrick Park, Mabelreign, Harare.

Tel +263 242 306 028. Email tungadex@gmail.com or d.chagwena@zvitambo.com

ABSTRACT

Introduction

Over one-quarter of children in sub-Saharan Africa are stunted; however, commercial supplements only partially meet child nutrient requirements, cannot be sustainably produced, and do not resolve physiological barriers to adequate nutrition (e.g. inflammation, microbiome dysbiosis and metabolic dysfunction). Redesigning current infant and young child feeding (IYCF) interventions using locally available foods to improve intake, uptake and utilization of nutrients could ameliorate underlying pathogenic pathways and improve infant growth during the critical period of complementary feeding, to reduce the global burden of stunting.

Methods and Analysis

Child Health Agriculture Integrated Nutrition (CHAIN) is an open-label, individually randomized trial comparing the effects of IYCF versus "IYCF-plus" on nutrient intake during infancy. The IYCF intervention comprises behaviour-change modules to promote infant nutrition delivered by village health workers, plus small-quantity lipid-based nutrient supplements (SQ-LNS) from 6-12 months of age which previously reduced stunting at 18 months of age by ~20% in rural Zimbabwe. The "IYCF-plus" intervention provides these components plus powdered NUA-45 bio-fortified sugar beans, whole egg powder, moringa leaf powder and pro-vitamin A maize. The trial will enrol 192 infants between 5-6 months of age in Shurugwi district, Zimbabwe. Research nurses will collect data plus blood, urine and stool samples at baseline (5-6 months of age) and endline (9-11 months of age). The primary outcome is energy intake, measured by multi-pass 24-hour dietary recall at 9-11 months of age. Secondary outcomes include nutrient intake, anthropometry and haemoglobin concentration. Nested laboratory sub-studies will evaluate the gut microbiome, environmental

For peer review only

For peer review only - http://bmjopen.bmj.com/site/about/guidelines.xhtml

enteric dysfunction, metabolic phenotypes and innate immune function. Qualitative sub-studies will explore the acceptability and feasibility of the IYCF-plus intervention among participants and community stakeholders, and the effects of migration on food production and consumption.

Ethics and Dissemination

This trial is registered at clinicaltrials.gov (NCT04874688) and was approved by the Medical Research Council of Zimbabwe (MRCZ/A/2679). Dissemination of trial results will be conducted through the Community Engagement Advisory Board in the study district and through national-level platforms.

Strengths and Limitations of this study:

- Efficient trial design building upon previous results from rural Zimbabwe.
- Community-based study utilising foods that could provide a sustainable solution to child growth in rural Africa.
- Improved infant and young child feeding may close nutrient gaps, ameliorate underlying pathogenic pathways and improve infant growth during the critical period of complementary feeding.
- Measurement of a broad range of biomarkers.
- Limitation of a short follow-up period to measure outcomes.

Trial registration: NCT04874688. Registered 6 May 2021- Retrospectively registered, https://clinicaltrials.gov/ct2/show/NCT04874688

For peer review only

INTRODUCTION

Undernutrition underlies 45% of child deaths among children <5 years[1]. Linear growth failure in childhood is the most prevalent form of undernutrition globally. An estimated 149 million children under 5 years of age are stunted, with a length-for-age Z-score (LAZ) more than two standard deviations below the population median[2]. Stunting affects almost one-third of children in sub-Saharan Africa, leading to reduced human capacity and increased long-term risk of chronic disease; it is therefore a surrogate marker of child health inequalities[1].

The period from 6-24 months of age is one of the most critical phases of linear growth[3], when stunting prevalence peaks due to high demand for nutrients coupled with limited quality and quantity of complementary foods[2]. Infant diets in rural sub-Saharan Africa often have low dietary diversity and a heavy reliance on white maize, which is high in starch and low in other nutrients. Interventions to improve infant and young child feeding (IYCF) typically include nutrition counselling to caregivers, plus a combination of commercial and locally available food products with or without micronutrients. However, a meta-analysis[4] of 42 studies showed only a modest impact of complementary feeding interventions on linear growth. Small-quantity lipid-based nutrient supplements (SQ-LNS), which are micronutrient-fortified ready-to-use products, show a small but measurable impact on LAZ.

We recently conducted the Sanitation Hygiene Infant Nutrition Efficacy (SHINE) trial[5], a 2×2 factorial cluster-randomized trial of improved water, sanitation and hygiene (WASH) and improved IYCF in rural Zimbabwe. A combination of IYCF messages and provision of SQ-LNS between 6-18 months of age improved LAZ of children at age 18 months by +0.16 (95%CI 0.08, 0.23) and reduced stunting by 20%[5]. The intervention also increased haemoglobin by 0.20 g/dL (95%CI 0.13, 0.28), and reduced anaemia by almost 25%[5].

For peer review only - http://bmjopen.bmj.com/site/about/guidelines.xhtml

However, despite this intensive IYCF intervention, 55%, 70%, and 20% of infants did not meet energy, folate and zinc/iron dietary intake requirements, respectively, and over one-quarter remained stunted. We also found evidence for barriers to infant nutrition, including caregiver capabilities[6], household characteristics[7], infant enteropathogen carriage[8], and systemic and intestinal inflammation[9,10], which were not resolved by the IYCF intervention[11]. Thus, we believe that persistent barriers to nutrient intake, uptake and utilization limited the impact of the IYCF intervention.

Nutrient intake is influenced by food insecurity; women's disempowerment to make decisions about land use, crop choice, and distribution of food within the household; and inequitable gender beliefs[7,12]. Nutrient uptake and utilization are influenced by intestinal pathologies which are highly prevalent among children in low-resource settings[3]. First, environmental enteric dysfunction (EED), a subclinical pathology of the small intestine characterized by intestinal inflammation and blunted villi, may impair efficient intestinal uptake of nutrients. Second, disturbance of the normal assembly of the gut microbiota may impair its roles in immune maturation, intestinal development, and nutrient metabolism, thereby impairing growth[13]. Third, systemic inflammation arising from gut pathology increases energy requirements, reduces circulating micronutrients, and inhibits the growth hormone axis[14]. Previously, barriers to intake, uptake and utilization of nutrients have largely been addressed in isolation; however, addressing these in parallel could ultimately improve growth and development in young children. Here, we present methodology for the Child Health Agriculture Integrated Nutrition (CHAIN) trial which aims to address each of these barriers together through a randomized IYCF intervention.

For peer review only - http://bmjopen.bmj.com/site/about/guidelines.xhtml

STUDY OVERVIEW

CHAIN is an open-label, individually-randomized trial comparing the effects of IYCF versus an enhanced IYCF intervention ("IYCF-plus") on energy and nutrient intake, growth and haemoglobin in infants at high risk of stunting. The overarching goal of this trial is to fill key nutrient gaps among infants in rural sub-Saharan Africa through an improved IYCF intervention using locally-available foods that could ultimately be sustainable through agriculture. Interventions will be delivered from 6-12 months of age in 192 children in rural Zimbabwe with a primary outcome of energy intake at 9-11 months of age. Our approach builds on the SHINE IYCF package, which reduced stunting but did not close all nutrient gaps[5]. CHAIN will test the impact of additional foods (powdered NUA-45 bio-fortified sugar beans, whole egg powder, moringa leaf powder and pro-vitamin A maize) that are nutrient-rich, culturally acceptable, locally sustainable and may have functional properties to ameliorate underlying pathogenic pathways, thereby tackling the identified barriers to nutrient intake, uptake and utilization. For the duration of the trial, these foods will be provided by village health workers as dried powders, which can be added to infant porridge as point-of-use fortificants. However, if shown to be efficacious, families could ultimately become self-sufficient through household-level agriculture.

STUDY OBJECTIVES

Objective 1. Evaluate the effect of an enhanced infant feeding intervention ("IYCF-plus") on energy intake at 9 months of age (window 9-11 months) in a randomized, community-based trial in rural Zimbabwe. *We hypothesize that provision of powdered fortificants (pro-vitamin A maize, NUA45 sugar beans, moringa and egg) for infants from 6 months of age will provide more energy at 9 months of age than the current standard-of-care IYCF intervention (trial primary outcome).*

For peer review only - http://bmjopen.bmj.com/site/about/guidelines.xhtml

Objective 2. Evaluate the impact of IYCF-plus on nutrient intake, growth, and haemoglobin in young children at risk of stunting. *We hypothesize that IYCF-plus will improve the intake of key nutrients (protein, iron, zinc and folate) in 9-month-old infants compared to the standard-of-care IYCF intervention, and that IYCF-plus will increase length-for-age, weight-for-age, weight-for-length and haemoglobin more than IYCF (all secondary outcomes).*

Objective 3. Evaluate the impact of IYCF-plus on biological barriers to nutrient uptake and utilisation. *We hypothesize that the IYCF-plus intervention will increase microbiota maturity, ameliorate EED, reduce systemic inflammation and improve innate immune function in children aged 9 months, compared to the standard-of-care IYCF intervention.*

Objective 4. Identify metabolic signatures of the IYCF-plus intervention in young children. *We hypothesize that the IYCF-plus intervention will increase the concentrations of essential amino acids and choline at 9 months of age more than the standard-of-care IYCF intervention.*

Objective 5. Explore the acceptability and feasibility of the IYCF-plus intervention among participants and community stakeholders utilizing qualitative methodology. *Information from this assessment will be shared with policymakers to help design a larger roll-out of this intervention at district, provincial, or national level.*

Objective 6. Explore the extent to which women's empowerment influences IYCF practices and nutrition outcomes in rural smallholder agricultural households.

For peer review only - http://bmjopen.bmj.com/site/about/guidelines.xhtml

We hypothesize that infants of women scoring in the highest tertile of the Women's Empowerment Agriculture Index (WEAI) will have improved dietary intake compared to infants of women in the lowest tertile of the WEAI index.

Objective 7. Identify the extent of migration and movement at the household level, explore the type, frequency and impact of any associated remittance flows on food consumption and production, and consider the importance of migration to any changes in established food cultures. *Information from this assessment will be shared with policymakers to help design a larger roll-out of this intervention at district, provincial, or national level.*

RATIONALE FOR INTERVENTIONS

The CHAIN trial will compare IYCF as tested in the SHINE trial[7] versus an enhanced IYCF intervention ("IYCF-plus"). IYCF comprises a set of sequential behaviour-change modules focusing on improved IYCF practices (e.g., nutrient density, feeding during illness, and dietary diversity), together with provision of daily SQ-LNS from 6-12 months of age, and powdered maize to make infant porridge. IYCF-plus comprises all the components of the IYCF intervention, plus four additional food supplements: pro-vitamin A (PVA) maize, NUA-45 sugar beans, moringa leaf powder, and whole egg powder. We have chosen this combination of 'functional' food supplements to close the remaining nutrient gaps for young children identified during SHINE (Table 1) and to ameliorate pathogenic pathways that impede uptake and utilization of nutrients.

Pro-Vitamin A (PVA) Maize is a bio-fortified maize rich in beta-carotenes, which is grown in Zimbabwe. Studies in neighbouring countries have shown that daily intake of PVA maize can improve the vitamin A status of children[15]. PVA maize appears less prone to contamination

For peer review only - http://bmjopen.bmj.com/site/about/guidelines.xhtml

with aflatoxin, which is a fungal toxin affecting agricultural crops during growth, storage and processing that may impair child growth[16].

NUA 45 sugar beans are a high-nutrient bean variety providing bio-fortified zinc and iron, a high protein efficiency ratio, plus folate and resistant starch. Bio-fortified beans significantly increased haemoglobin, serum ferritin, and body iron in Rwandan women[17].

Moringa oleifera is a widespread crop in Zimbabwe. Dried moringa leaves can be ground into a powder providing a rich source of protein, fibre, mineral and micronutrients, including vitamin A, calcium, folate, vitamin C and vitamin E, and antioxidant polyphenols. Moringa leaf powder is available in shops in Zimbabwe as a food supplement. Pilot studies show that moringa leaf powder is safe and widely accepted as a dietary supplement by children and caregivers in sub-Saharan Africa[18,19], but there have been no randomized trials.

Whole egg powder is commercially available, easily reconstituted and retains the nutrient content of whole eggs. Egg production is common in rural Zimbabwe. One egg per day for 6 months to children between 6-15 months of age increased LAZ by 0.63 in Ecuador[20]. This large effect is likely attributable to high-quality protein and choline, which are critical nutrients for linear growth[21 22]. Eggs contain all nine essential amino acids in proportions that closely match infant requirements for organ and muscle mass accretion. Choline is an essential nutrient that promotes growth in animal models[23]. Children with EED have reduced circulating concentrations of phosphatidylcholines[24], which are required for chondrogenesis at the growth plate, and reliant on adequate dietary intake of choline. One egg meets the majority of an infant's daily requirement. Eggs are also high in fat, energy-dense, and make modest but important contributions to vitamin A, iron, and zinc intake.

For peer review only - http://bmjopen.bmj.com/site/about/guidelines.xhtml

Together, these food supplements have the added plausible benefit of improving the microbiota and gut barrier function and reducing intestinal and systemic inflammation (Figure 1). The IYCF-plus intervention will increase dietary diversity and micronutrient content, which has been shown to promote healthy gut microbiota composition[25]. The resistant starch present in legumes is readily fermented by the gut microbiota to produce short-chain fatty acids, which act as a primary energy source for enterocytes, and other metabolites that maintain the integrity of the intestinal barrier[26]. Recent trials suggest that legumes modestly improve linear growth and intestinal permeability[27,28]; these effects may be enhanced through integration with other micronutrients such as vitamin A and zinc, which can improve gut barrier function[29,30]. Legume intake and high dietary quality scores have been associated with reduced systemic inflammation[31]. Finally, pre-clinical studies have reported a role for moringa in reducing oxidative stress and improving immune function[32]. We hypothesize that children receiving the IYCF-plus intervention will have a more mature microbiota, reduced EED, less systemic inflammation and an associated improvement in anti-pathogen immune cell function compared to the IYCF intervention.

Ultimately, through improved agriculture and animal husbandry practices families could become self-sufficient in producing the beans, biofortified maize, moringa and eggs used for the IYCF-plus intervention for their household. Furthermore, these inputs have useful synergies: for example, moringa pods and maize bran provide food for chickens that improves their feed efficiency and increases egg production.

For peer review only - http://bmjopen.bmj.com/site/about/guidelines.xhtml

FORMATIVE RESEARCH AND COMMUNITY ENGAGEMENT

Formative work explored delivery and acceptability of the new food supplements proposed in CHAIN[33]. Briefly, this qualitative study purposively sampled nine Village Health Workers (VHW) from the Shurugwi rural community where SHINE was conducted, in addition to 27 caregivers of children between 6-18 months of age. The aims of this formative work were to assess feasibility of delivering bean, egg and moringa powder to families; the acceptability of recipes devised to incorporate the new supplements into usual foods; and to inform IYCF-plus behavioural modules.

Activities included focus group discussions with mothers, group-based recipe formulation, testing and review as well as home visits to assess ingredient uptake, usage, storage and recipe adherence and innovations. Household observations and views from extended family members indicated high acceptability of the new ingredients. Sensory evaluation by mothers who formulated and standardised the recipes indicated high acceptability of the complementary food recipes. All formative study participants participated in developing the behaviour-change messages and finalization of the recipes in a recipe book developed for use in the CHAIN trial IYCF-plus intervention[33].

STUDY SETTING

The CHAIN trial is being conducted in Shurugwi district, Zimbabwe. This is a predominantly rural, subsistence farming area, with 15% antenatal HIV prevalence and 35% stunting prevalence[7]. VHWs are a community-level cadre of healthcare workers within the Ministry of Health and Child Care. They will sensitize families in their catchment area between birth and 5 months of age about the CHAIN study and will refer those who are interested to the trial team. A research nurse will visit the household to undertake screening and written informed

For peer review only - http://bmjopen.bmj.com/site/about/guidelines.xhtml

For peer review only

consent between 5-6 months of age. Interventions, delivered by VHWs, will start as soon as possible after randomization, and the primary outcome will be measured by research nurses at endline.

TRIAL OUTCOMES

The primary outcome is energy intake at 9 months of age (visit window 9-11 months), as measured by multi-pass 24-hour dietary recall. Secondary and tertiary outcomes are defined in Table 2.

INCLUSION CRITERIA

- Age between 5-6 months
- Planning to live in the study area for the duration of the trial

EXCLUSION CRITERIA

- Severe infant disability that interferes with feeding
- Known allergy to peanuts or eggs

RECRUITMENT AND BASELINE DATA COLLECTION

The trial schedule is shown in Table 3. Households are identified through VHW registers which record all women of reproductive age. A research nurse will visit the family's homestead to screen the child for eligibility, provide information on the trial, and undertake written informed consent in Shona or Ndebele. Baseline data will be collected via questionnaire on maternal, infant and household characteristics. Maternal and infant height, weight and mid-upper arm circumference will be measured. Infant samples of stool, urine and blood will be collected for assessment of laboratory analyses, including immunology assays,

For peer review only

microbiome and metabolic assessments (full details below). Haemoglobin will be measured at point-of-collection using a HemoCue 301 machine.

RANDOMIZATION

The randomization schema was pre-prepared by the trial statistician, using random permuted blocks, with a 1:1 allocation to IYCF or IYCF-plus. Randomization codes are securely embedded in the trial database so that the next number is accessible to the data officer, but not the entire randomization list. Twins are allocated to the same trial arm. The VHW, supervised by an intervention nurse, visits the mother to tell her the trial allocation and to begin the interventions. It is not possible to blind households or fieldworkers to the interventions, but data and laboratory analysts are blinded to the allocated arm.

INTERVENTION DELIVERY

Behavioural modules: A total of nine face-to-face modules will be delivered to caregivers in each arm by VHWs during 10 home visits, which coincide with key infant ages, so that sequential age-appropriate messages about complementary feeding are introduced and reinforced (Table 4). The VHW introduces the food supplements in both arms, demonstrates how to add them to food, monitors for any adverse reactions and provides monthly re-supplies. Modules are interactive and are delivered to all household members present. The last module will be delivered at 11 months of age, with infant food supplements provided until 12 months of age, when all trial interventions end. Using this design, we will ensure that all infants are still receiving the IYCF or IYCF-plus interventions when endline data collection occurs at 9 months (window 9-11 months) of age.

For peer review only

If a module is missed, the VHW will try to catch up by scheduling a new date, or else will summarize the missed module at the next scheduled visit. If a caregiver moves within the study area, the VHW covering that area will deliver modules to the caregiver, where possible; if the caregiver moves out of the study area, she will not receive study modules or food supplements.

IYCF arm: Core IYCF counselling modules are complemented by provision of one sachet of 20g SQ-LNS daily between 6-12 months of age. SQ-LNS is a peanut-based supplement rich in calories, protein and micronutrients, which can be consumed directly from the sachet or mixed with porridge. Families also receive a daily infant ration of white maize to feed the baby as porridge.

IYCF-plus arm: As in the IYCF arm, core counselling modules focusing on complementary feeding are delivered by the VHW, with provision of one sachet per day of 20g SQ-LNS between 6-12 months of infant age. In addition, children receive NUA-45 biofortified bean powder, whole egg powder and moringa leaf powder. The quantity of food supplements provided is based on the child's age to ensure the daily recommended nutrient intake is met (Table 5). Families also receive a daily infant ration of PVA biofortified maize to feed the baby as porridge. Supplements are delivered in sealed containers, which the mother is asked to keep in a cool part of the house. Six recipes promoting high-quality staple foods, which were developed and standardized in the formative studies[33], are outlined in a recipe book, with cooking demonstrations given by the VHW.

For peer review only

INTERVENTION DELIVERY AND UPTAKE

Each VHW will deliver the intervention to 1-3 enrolled households during the study. Eight intervention nurses are responsible for monitoring delivery of modules and food supplements and evaluating intervention uptake and compliance to recommended behaviours by caregivers. Intervention nurses provide supportive supervision to VHWs by scheduled attendance at some household visits to provide feedback and by conducting unscheduled spot checks. Intervention nurses hold monthly meetings with the VHWs they supervise, to share learning, capture data on module delivery, and provide re-training as needed. A module delivery and intervention uptake checklist is completed by VHWs at each module delivery visit, and submitted to intervention nurses during monthly meetings. The checklist records modules that have been successfully delivered and dates of delivery. Data on uptake of interventions and compliance to recommended behaviours will include utilization of food supplements, any sharing of food supplements observed and involvement of other family members in child feeding assessed by a caregiver questionnaire.

FOLLOW-UP DATA COLLECTION

Households will be visited by a research nurse at 9 months of infant age (window 9-11 months) for endline data collection. Infant weight, length, MUAC, and head circumference will be measured, and samples of blood, urine and stool collected. Haemoglobin measurement by point-of-care HemoCue 301 machine will be repeated. Children with symptomatic mild to moderate anaemia (<11 g/dL) or with severe anaemia (<7 g/dL) will be referred to local clinics. Children with moderate or severe acute malnutrition (MUAC <125mm, or weight-for-length Z-score <-2) will also be referred to local clinics.

For peer review only - http://bmjopen.bmj.com/site/about/guidelines.xhtml

The trial primary outcome will be measured by research nurses via 24-hour multi-pass dietary recall. A subgroup of 50% of randomly selected households will have a second 24-hour dietary recall visit approximately one week later. This method provides a robust and validated measure of nutrient intake based on a comprehensive and standardized assessment. The dietary recall method assesses all food and beverages consumed in the previous 24 hours (including supplements provided by the trial) and comprises five passes. In the first pass, the research nurse asks the caregiver to list all foods consumed by the child during the last day, and to list any night feeds. In the second pass, the caregiver is asked to list all activities they undertook, and whether they fed the child food between activities; this helps the caregiver remember all feeding episodes. In the third pass, more details about foods and beverages are collected, including the time and place of preparation, ingredients, and brand of foods given. In the fourth pass, the caregiver estimates the portion size fed to the child. Research nurses carry samples of the most commonly consumed foods and ask the caregiver to estimate the amount fed to the child. The fieldworker then transfers the estimated portion to a standard cup, spoon or digital scale for recording. In the final pass, the caregiver recalls if there were any foods or meals that have not already been mentioned. Caregivers are also asked about the general health of the child on the previous day, whether the child's intake was less or more than usual, and how many times the child was breastfed.

For peer review only

Actual energy and nutrient intakes will be compared with WHO-estimated energy requirements, the Institute of Medicine, Food and Nutrition Board recommended daily allowances for protein and choline, and the WHO-recommended nutrient intakes (RNIs) for other vitamins and minerals. Required nutrient intake from complementary foods is calculated by subtracting the amount of energy/nutrient in an average intake of breast milk from the total requirement. Energy requirements are calculated as kilojoules required per

For peer review only - http://bmjopen.bmj.com/site/about/guidelines.xhtml

kilogram of body weight for breastfed children[34]; however, we will apply the slightly higher energy requirement estimated by Butte for children in low-income settings, which reflect increased needs due to greater infection burden[35]. Protein requirements will be defined as the WHO-recommended safe level of protein intake for 9-11 month old children[36]. Fat requirement will be defined as 35% energy requirement, which is the mid-point of several recommendations[37]. Micronutrient requirements will be defined as WHO-recommended nutrient intakes, except for calcium which is defined as the mean of the WHO RNI and US RNI. For zinc and iron, we will assume 30% and 10% bioavailability, respectively[23,34,38,39]. For breastfed children, we estimate the required nutrient intake from complementary foods by subtracting the amount of each nutrient in breast milk from the total requirement[39]. Using this comprehensive approach, we will determine the impact of the IYCF-plus versus IYCF intervention on energy intake (primary outcome) and the relative contributions of supplements (including SQ-LNS) and other complementary foods in closing infant nutrient gaps across trial arms. In addition to assessing total protein intake, we will explore essential amino acid intake, digestibility-adjusted protein intake and inflammation-adjusted protein intake[40].

DATA COLLECTION AND MANAGEMENT

Research data are collected onto electronic case report forms (CRFs) using pre-programmed tablets, with Open Data Kit (ODK) software. Full data validation procedures are programmed into the tablets including embedded skip patterns, data completeness and plausibility checks. Back-up paper CRFs are carried by research nurses in the event of tablet failure. Data are uploaded from tablets onto a secure trial database daily and backed up onto a secure cloud database hosted on Microsoft Azure.

For peer review only

Each participant is allocated a participant identifier which is used on all forms to identify the child. Personal information and data are kept confidential and managed in accordance with the requirements of the Medical Research Council of Zimbabwe. Paper records (e.g., CRF, clinical / laboratory information and test results) will be entered into the electronic database; source documents will be stored in a secure, locked cupboard at each study site, and kept fully confidential. Data will be kept securely on a password-protected customised MS-SQL Server trial database and hosted by Microsoft Azure.

SAMPLE SIZE

The sample size of 192 infants assumes 10% loss to follow-up due to withdrawal and infant deaths, meaning there will be an estimated 86 evaluable infants per group at endline. This sample size provides 86% power at 5% significance to detect a 20% increase in the proportion of infants achieving their recommended energy intake (by 24-hour dietary recall) in the IYCF-plus arm, assuming that only 65% of infants are meeting requirements in the IYCF (standard-of-care) arm based on SHINE data. If loss-to-follow is as high as 15%, we will still have 80% power to detect a 20% increase in the proportion reaching their daily energy intake (study primary outcome).

STATISTICAL ANALYSIS

Analysis of trial outcomes will be by intention-to-treat. P values will be 2-sided and interpreted as significant if $p<0.05$. Binary outcomes will be compared between groups using the Chi-square test and logistic regression to compute odds ratios and corresponding 95% confidence intervals. Other categorical outcomes with more than two levels will be compared between groups using Chi-squared tests and multinomial regression. Continuous outcomes will be compared using simple t-tests and linear regression. Non-normal continuous outcomes

For peer review only

will be transformed appropriately before analysis. Robust standard error estimates will be used to estimate confidence intervals. All analyses will be pre-specified in a Statistical Analysis Plan and posted online at Open Science Framework before analyses begin (https://osf.io/njy2a/). A per protocol analysis will be conducted using adherence data to pre-define the per protocol population. We will undertake two subgroup analyses: i) by infant sex; and ii) by maternal HIV status, if we find some evidence of interactions with the intervention (p<0.10).

LABORATORY ANALYSES

Biological samples will be used to evaluate the nutrient profiles, EED, systemic inflammation, innate immune function, metabolic phenotype and the gut microbiota, as shown in Table 6.

EED and systemic inflammation: We will use a combination of plasma and stool ELISA assays to compare the impact of IYCF and IYCF-plus on EED, by characterising the hypothesised causal pathway from the gut to growth, measuring markers of intestinal inflammation (stool myeloperoxidase, neopterin) small intestinal damage (plasma I-FABP), intestinal permeability (alpha-1 antitrypsin), microbial translocation from the gut (plasma LBP, sCD14), systemic inflammation (AGP, CRP, TNFα) and growth hormone activity (plasma IGF-1). We will also measure aflatoxin M1 in urine, to assess recent exposure to dietary aflatoxin, which is a plausible cause of EED and may be reduced in the IYCF-plus arm since PVA maize appears less prone to fungal contamination.

Immune function: Immune cell activation is metabolically costly and chronic activation by recurrent infections/EED may create a barrier to children meeting their nutrient requirements

For peer review only - http://bmjopen.bmj.com/site/about/guidelines.xhtml

by: i) driving inflammation and oxidative stress, ii) contributing to enteropathy, iii) compromising the capacity of innate immune cells to defend against new infections, which further deplete dietary nutrients. Multiple innate and adaptive immune mediators are dysregulated in undernourished children[41], but little is known about if/how nutritional interventions affect immune defences[42]. We will use whole blood samples to compare innate immune cell phenotype and function between randomised groups. We will quantify surface expression of activation markers HLA-DR, CD64 and CD16 on blood monocytes, and CD64 and CD62L on blood monocytes and neutrophils via flow cytometry. To characterise the functional capacity of innate immune cells to respond to pathogen challenge we will quantify pro-inflammatory cytokine secretion in supernatants derived from whole blood cultures with and without bacterial lipopolysaccharide (LPS). Whole blood culture with and without fluorescent-labelled *Escherichia coli*-coated bioparticles will be used to quantify uptake of bacteria via flow cytometry.

Metabolic phenotyping: A targeted ultra-performance liquid chromatography-mass spectrometry (UPLC-MS)-based assay will be used to measure tryptophan-related metabolites in plasma[43]. This includes metabolites involved in the kynurenine, serotonin, and indole pathways. In addition, downstream NAD+ related metabolites, such as nicotinic acid, nicotinamide, and nicotinamide-riboside will be measured together with markers of systemic inflammation (neopterin), enterocyte mass (citrulline) and the neurotransmitter dopamine. ^{1}H nuclear magnetic resonance (NMR) spectroscopy will be used to characterize the metabolic profiles of urine, plasma and faecal water samples. This approach measures H-containing metabolites present above the limit of detection in the samples in an untargeted manner. This captures information on amino acids, gut microbial metabolites, and metabolites involved in

For peer review only - http://bmjopen.bmj.com/site/about/guidelines.xhtml

choline and energy metabolism. It may also be used to study dietary components and assess variation in their digestion.

Microbiome sequencing: Whole metagenome shotgun sequencing will be employed on stool samples to examine the effect of the trial interventions on the gut microbiome and its association with growth. DNA will be extracted from stool aliquots (200mg) using the Qiagen PowerFecal Pro DNA kit, followed by metagenomic sequencing library preparation. Following qualitative and quantitative assessment of sequencing libraries, sequencing will be performed via the HiSeq 2500 platform producing 6-10 million sequencing reads per sample. Following quality control and trimming of human reads, sequencing reads will be processed through validated pipelines to generate compositional (MetaPhlAn v.3) and functional (HUMAnN v.3) readouts of the gut microbiome. Microbiome maturity will be assessed as previously described using a control dataset generated from the SHINE trial[44]. Aliquots of stool stored in glycerol will be used to isolate microorganisms of interest for downstream experiments assessing the influence of the gut microbiome on EED and growth.

QUALITATIVE STUDIES

The first qualitative substudy will develop a more in-depth understanding of how the CHAIN interventions were integrated into household nutrition practices, and the cultural, economic, and social processes that shape this. Up to 20 families will be purposively sampled based on type of household, caregiver characteristics and trial arm. Semi-structured interviews will be conducted to explore a range of themes, including any changes in the participants' receptiveness to the food supplements or children's response to them, the ability to maintain compliance with the food preparation guidelines, and challenges they may have experienced (e.g., accessing or storing the food supplements). The interviews will also focus on relevant

For peer review only

cultural, economic, and social processes operating at the household level that may influence food preparation and consumption practices and explore how these shape the sustainability of the intervention. Additionally, emphasis will be placed on exploring the role that household and area-level gender dynamics may play in decision-making practices. In addition to the household participants, we will include a sample of 10 VHWs involved in delivering the interventions, as well as formal and informal leaders. Focus group discussions will consider stakeholders' understanding of the intervention and any issues they believe would need to be addressed for wider community roll-out. In addition, the focus groups will provide important information about the wider context within which the household participants operate.

A second qualitative substudy will identify the ways in which household migration (defined here as people who have moved away from a household for 3 months or more) influences food security. Up to 30 participating households will be identified from the baseline survey as having at least one family member who has migrated across a range of geographical scales (local, regional, national, and international) and where possible representing different household types (including, male- or female-headed households, orphan-headed households, elder-headed households). In-depth interviews will be conducted with study participants to explore the interactions that exist between household migration and remittance practices (receiving and sending) and their potential to influence household food consumption and production practices. In addition, the in-depth interviews aim to consider the importance of geographical scale to remitting practices and their influence on food consumption and production and investigate possible interactions between migration and household participation in the CHAIN intervention.

For peer review only - http://bmjopen.bmj.com/site/about/guidelines.xhtml

Interviews and focus groups will be audio recorded, for subsequent transcription and translation; transcripts will be entered into NVivo for coding, analysis, and interpretation. Analysis will proceed using both deductive and inductive approaches and will utilise the framework method often employed in multidisciplinary health-related research. All audio recordings will be destroyed, although transcriptions will be stored securely and made available for future analysis as required.

TRIAL RISKS AND ADVERSE EVENTS

All interventions are commercially available foods (beans, egg powder, moringa, maize) or food supplements that are widely used globally (SQ-LNS). Peanuts and eggs are both staple foods in rural Zimbabwe; although both are potentially allergenic among infants in high-income settings, the prevalence of food allergies in sub-Saharan Africa is extremely low[5,45,46]. SQ-LNS, which contains peanuts, was used in the same community in the SHINE trial among more than 2000 infants. From over 365,000 doses of SQ-LNS that were given to infants in SHINE, there were no allergic reactions, and no serious adverse events[5]. Only two adverse events were possibly or probably related to SQ-LNS; both resolved without sequelae[5]. Children with a known allergy to peanuts or eggs will be excluded from the study to further minimise the risk of adverse events. All adverse events will be reported and reviewed by the study physician, and a tabulated monthly summary will be sent to an independent safety monitor. All serious adverse events and trial-related adverse events will be reported to the Medical Research Council of Zimbabwe according to established timeframes.

The COVID-19 pandemic poses a potential risk to research activities in the community**.** We will ensure that research staff wear personal protective equipment and practice physical distancing to keep themselves and research participants safe. Interviews will be conducted in

For peer review only

a confidential outdoor part of the homestead wherever possible. VHWs conduct their activities with guidance for safe working from the Ministry of Health and Child Care. Procedures will be reviewed as the pandemic progresses, and any changes discussed with the District Health Executive and with the Medical Research Council of Zimbabwe.

ETHICS

Ethical approval was granted by the Medical Research Council of Zimbabwe, and Sponsorship provided by Queen Mary University of London. All mothers will provide written informed consent. Information and consent procedures will be administered in each mother's language of choice (Shona, Ndebele and/or English). Because mothers often need to consult with other family members before deciding about their child's participation in a trial, we will include other family members in consent discussions if the mother wishes. Illiterate mothers who understand a verbal explanation of the study can provide a thumb imprint on the consent form in the presence of an independent witness. Mothers aged between 15-18 years are considered emancipated minors under Zimbabwean law.

Since the IYCF intervention in the SHINE trial led to 20% reduction in stunting[5], we are providing IYCF as the enhanced standard-of-care for the control arm, since we believe it is unethical not to provide this intervention in the exact same population. Provision of food supplements may confer benefits for infant nutrition and growth, and for household wellbeing, but we believe it is ethically justifiable to randomize the supplements of powdered egg, NUA45 sugar beans, moringa and PVA maize, since none are routinely provided to households in the community and there is scientific equipoise as to whether these food supplements will bring additional benefit to what is already being provided in the IYCF arm.

For peer review only - http://bmjopen.bmj.com/site/about/guidelines.xhtml

DISSEMINATION

Trial results will be presented at international conferences and published in open-access journals. Data will be available on request, after publication of the primary trial findings, by contacting the Trial Management Group, with details of data access requests available on the Zvitambo website. Results will be presented to the Ministry of Health and Child Care in Zimbabwe and will be disseminated to the study district through the Community Engagement Advisory Board which comprises peers selected by the community to review ongoing research studies in the community. Results will also be presented to UNICEF to inform the design and scale-up of IYCF programmes in Zimbabwe.

PATIENT AND PUBLIC INVOLVEMENT

Research questions and outcome measures was informed from gaps identified in the SHINE Trial, incorporating patients' priorities, experiences, and preferences. Patients who participated in the Formative study and Community Engagement Advisory Board informed the design of the CHAIN Trial and the burden of the intervention. Trial findings will be disseminated to patients through community engagement meetings and a video describing the Trial process and findings in the local language.

DISCUSSION

Stunting remains a global health challenge which hinders human capital and perpetuates poverty. There is an urgent need for more efficacious nutrition-specific interventions to enhance child linear growth during complementary feeding. Currently, multiple barriers constrain nutrient intake, uptake and utilization, including marginal diets, EED, perturbations of the microbiome, metabolic dysregulation and chronic inflammation. Current IYCF approaches partly close nutrient gaps but require optimisation to fully restore healthy child

For peer review only

growth. Utilising locally available foods with functional properties to supplement current IYCF approaches could have the dual goal of closing nutrient gaps in infancy and ameliorating pathogenic barriers to nutrient uptake and utilisation. We will test these ideas using a rigorous trial design in a rural, subsistence farming community with a high burden of stunting, and evaluate acceptability, feasibility and sustainability of the approach for longer-term and larger-scale deployment. Identifying new, sustainable ways to improve dietary quality and reduce stunting would help to accelerate progress towards 2030 global targets, and could have major benefits for long-term health, development and human capital.

DECLARATIONS

Ethics approval and consent to participate: Ethical permission has been granted by the Medical Research Council of Zimbabwe (MRCZ/A/2679). All mothers will provide written informed consent to participate.

Authors' contributions: Trial design: LES, CDB, RCR, NVT, JC, TN, TB, KD, KM, JS, PK, RN, AJP. Secured funding: LES, CDB, RCR, NVT, JC, TN, TB, KD, KM, JS, PK, RN, AJP. Agriculture design and expertise: JC, TN. Laboratory design and methods development: CDB, RCR, KM, JS, PK, AJP. Data management and analysis plan: BM, BC, RN. Formative work: LES, DC, SF, NVT, LFL, TB, KD, BM, DC, AJP. Qualitative design and expertise: LES, TB, KD, DC, SF, NVT, LFL, MM. Training and implementation: AT, BM, DC, KM. Study oversight: LES, JC, LFL, DC, RN, AJP. All authors read and approved the final manuscript.

Acknowledgements: We appreciate the Community Engagement Advisory Board and Families who supported in the design of the study trial.

For peer review only - http://bmjopen.bmj.com/site/about/guidelines.xhtml

Funding: UK Research and Innovation Biotechnology and Biological Sciences Research Council (UKRI-BBSRC), from the Global Challenges Research Fund (GCRF) under Food & Nutrition Research for Health in the Developing World: Bioavailability and Nutrient Content. RCR and AJP are funded by Wellcome (grants 206455/Z/17/Z and 108065/Z/15/Z). CDB is funded by a joint award from Wellcome and the Royal Society (206225/Z/17/Z). The second qualitative sub-study is funded by the Arts and Humanities Research Council (AHRC), UK (AH/T004428/1). Funding bodies had no role in the study design, implementation, analysis and interpretation of the data.

Competing interests: The authors declare that they have no competing interests.

For peer review only

REFERENCES

1. Black RE, Victora CG, Walker SP, et al. Maternal and child undernutrition and overweight in low-income and middle-income countries. *Lancet.* 2013;382(9890):427-451.

2. UNICEF: Levels and trends in child malnutrition, United Nations Children's fund (UNICEF). World Health Organization, International Bank for Reconstruction and Development/The World Bank Levels and trends in child malnutrition: key findings of the 2019 edition of the joint child malnutrition estimates Geneva: World Health Organization 2019.

3. Prendergast AJ, Humphrey JH. The stunting syndrome in developing countries. *Paediatr Int Child Health.* 2014;34(4):250-265.

4. Dewey KG, Adu-Afarwuah S. Systematic review of the efficacy and effectiveness of complementary feeding interventions in developing countries. *Matern Child Nutr.* 2008;4 Suppl 1:24-85.

5. Humphrey JH, Mbuya MNN, Ntozini R, et al. Independent and combined effects of improved water, sanitation, and hygiene, and improved complementary feeding, on child stunting and anaemia in rural Zimbabwe: a cluster-randomised trial. *Lancet Glob Health.* 2019;7(1):e132-e147.

6. Matare CR, Mbuya MN, Dickin KL, et al. Maternal Capabilities are associated with child caregiving behaviors among women in rural Zimbabwe. *The Journal of Nutrition.* 2020.

7. Humphrey J, Mbuya M, Ntozini R. Sanitation Hygiene Infant Nutrition Efficacy (SHINE) Trial Team. Independent and combined effects of improved water, sanitation, and hygiene, and improved complementary feeding, on child stunting and

For peer review only

anaemia in rural Zimbabwe: a cluster-randomised trial. *Lancet Glob Health.* 2019;7:e132-147.

8. Rogawski McQuade ET, Platts-Mills JA, Gratz J, et al. Impact of water quality, sanitation, handwashing, and nutritional interventions on enteric infections in rural Zimbabwe: the Sanitation Hygiene Infant Nutrition Efficacy (SHINE) Trial. *The Journal of infectious diseases.* 2020;221(8):1379-1386.

9. Gough EK, Moulton LH, Mutasa K, et al. Effects of improved water, sanitation, and hygiene and improved complementary feeding on environmental enteric dysfunction in children in rural Zimbabwe: A cluster-randomized controlled trial. *PLoS Negl Trop Dis.* 2020;14(2):e0007963.

10. Mutasa K, Ntozini R, Mbuya MNN, et al. Biomarkers of environmental enteric dysfunction are not consistently associated with linear growth velocity in rural Zimbabwean infants. *Am J Clin Nutr.* 2021.

11. Gough EK, Moulton LH, Mutasa K, et al. Effects of improved water, sanitation, and hygiene and improved complementary feeding on environmental enteric dysfunction in children in rural Zimbabwe: A cluster-randomized controlled trial. *PLoS neglected tropical diseases.* 2020;14(2):e0007963.

12. Desai A, Smith LE, Mbuya MN, et al. The SHINE trial infant feeding intervention: pilot study of effects on maternal learning and infant diet quality in rural Zimbabwe. *Clinical Infectious Diseases.* 2015;61(suppl_7):S710-S715.

13. Robertson RC, Manges AR, Finlay BB, Prendergast AJ. The human microbiome and child growth–first 1000 days and beyond. *Trends in microbiology.* 2019;27(2):131-147.

14. Prendergast AJ, Rukobo S, Chasekwa B, et al. Stunting is characterized by chronic inflammation in Zimbabwean infants. *PLoS One.* 2014;9(2):e86928.

For peer review only - http://bmjopen.bmj.com/site/about/guidelines.xhtml

15. Palmer AC, Craft NE, Schulze KJ, et al. Impact of biofortified maize consumption on serum carotenoid concentrations in Zambian children. *Eur J Clin Nutr.* 2018;72(2):301-303.

16. Smith L, Stoltzfus R, Prendergast A. Food-Chain Mycotoxin Exposure, Gut Health and Impaired Growth: A conceptual framework *Advances in Nutrition.* 2012;3:1-6.

17. Haas JD, Luna SV, Lung'aho MG, et al. Consuming Iron Biofortified Beans Increases Iron Status in Rwandan Women after 128 Days in a Randomized Controlled Feeding Trial. *J Nutr.* 2016;146(8):1586-1592.

18. Barichella M, Pezzoli G, Faierman SA, et al. Nutritional characterisation of Zambian Moringa oleifera: acceptability and safety of short-term daily supplementation in a group of malnourished girls. *Int J Food Sci Nutr.* 2019;70(1):107-115.

19. Boateng L, Nyarko R, Asante M, Steiner-Asiedu M. Acceptability of Complementary Foods That Incorporate Moringa oleifera Leaf Powder Among Infants and Their Caregivers. *Food Nutr Bull.* 2018;39(1):137-148.

20. Iannotti LL, Lutter CK, Stewart CP, et al. Eggs in Early Complementary Feeding and Child Growth: A Randomized Controlled Trial. *Pediatrics.* 2017;140(1).

21. Semba RD, Shardell M, Sakr Ashour FA, et al. Child Stunting is Associated with Low Circulating Essential Amino Acids. *EBioMedicine.* 2016;6:246-252.

22. Ghosh S, Suri D, Uauy R. Assessment of protein adequacy in developing countries: quality matters. *Br J Nutr.* 2012;108 Suppl 2:S77-87.

23. Zeisel SH, Blusztajn JK. Choline and human nutrition. *Annu Rev Nutr.* 1994;14:269-296.

24. Baron J, Savendahl L, De Luca F, et al. Short and tall stature: a new paradigm emerges. *Nat Rev Endocrinol.* 2015;11(12):735-746.

For peer review only

25. Heiman ML, Greenway FL. A healthy gastrointestinal microbiome is dependent on dietary diversity. *Mol Metab.* 2016;5(5):317-320.

26. Kelly CJ, Zheng L, Campbell EL, et al. Crosstalk between Microbiota-Derived Short-Chain Fatty Acids and Intestinal Epithelial HIF Augments Tissue Barrier Function. 2015;17(5):662-671.

27. Agapova SE, Stephenson KB, Divala O, et al. Additional Common Bean in the Diet of Malawian Children Does Not Affect Linear Growth, but Reduces Intestinal Permeability. *J Nutr.* 2018;148(2):267-274.

28. Stephenson KB, Agapova SE, Divala O, et al. Complementary feeding with cowpea reduces growth faltering in rural Malawian infants: a blind, randomized controlled clinical trial. *Am J Clin Nutr.* 2017;106(6):1500-1507.

29. de Medeiros P, Pinto DV, de Almeida JZ, et al. Modulation of Intestinal Immune and Barrier Functions by Vitamin A: Implications for Current Understanding of Malnutrition and Enteric Infections in Children. *Nutrients.* 2018;10(9).

30. Wapnir RA. Zinc deficiency, malnutrition and the gastrointestinal tract. *J Nutr.* 2000;130(5S Suppl):1388S-1392S.

31. Esmaillzadeh A, Azadbakht L. Legume consumption is inversely associated with serum concentrations of adhesion molecules and inflammatory biomarkers among Iranian women. *J Nutr.* 2012;142(2):334-339.

32. Abdel-Latif M, El-Shahawi G, Aboelhadid SM, Abdel-Tawab H. Modulation of murine intestinal immunity by Moringa oleifera extract in experimental hymenolepiasis nana. *J Helminthol.* 2018;92(2):142-153.

33. DT C, S F, NV T, et al. Formulation and acceptability of local nutrient-dense foods for young children: A formative study for the Child Health Agriculture Integrated Nutrition (CHAIN) Trial in rural Zimbabwe.2021 (In preparation).

For peer review only

34. University UN, Organization WH. *Human Energy Requirements: Report of a Joint FAO/WHO/UNU Expert Consultation: Rome, 17-24 October 2001.* Food & Agriculture Org.; 2004.

35. Butte NF. Energy requirement of infants. *Public Health Nutrition.* 2009;8(7A):953-967.

36. WHO J. Protein and amino acid requirements in human nutrition. *World Health Organization technical report series.* 2007(935):1.

37. Uauy R, Castillo C. Lipid requirements of infants: implications for nutrient composition of fortified complementary foods. *The Journal of nutrition.* 2003;133(9):2962S-2972S.

38. Micronutrients IoMPo. *Dietary Reference Intakes for Energy, Carbohydrate, Fiber, Fat, Fatty Acids, Cholesterol, Protein and Amino Acids. Panel on Macronutrients Panel on the Definition of Dietary Fiber, Subcommittee on Upper Reference Levels of Nutrients, Subcommittee on Interpretation and Uses of Dietary Reference Intakes, and the Standing Committee on the Scientific Evaluation of Dietary Reference Intakes, Food and Nutrition Board.* National Academies Press; 2005.

39. Dewey KG, Brown KH. Update on technical issues concerning complementary feeding of young children in developing countries and implications for intervention programs. *FOOD AND NUTRITION BULLETIN-UNITED NATIONS UNIVERSITY-.* 2003;24(1):5-28.

40. Callaghan M, Oyama M, Manary M. Sufficient protein quality of food aid varies with the physiologic status of recipients. *The Journal of nutrition.* 2017;147(3):277-280.

41. Rytter MJH, Kolte L, Briend A, Friis H, Christensen VB. The immune system in children with malnutrition—a systematic review. *PloS one.* 2014;9(8):e105017.

For peer review only

42. Bourke CD, Jones KD, Prendergast AJ. Current understanding of innate immune cell dysfunction in childhood undernutrition. *Frontiers in immunology.* 2019;10:1728.

43. Whiley L, Nye LC, Grant I, et al. Ultrahigh-Performance Liquid Chromatography Tandem Mass Spectrometry with Electrospray Ionization Quantification of Tryptophan Metabolites and Markers of Gut Health in Serum and Plasma-Application to Clinical and Epidemiology Cohorts. *Anal Chem.* 2019;91(8):5207-5216.

44. Subramanian S, Huq S, Yatsunenko T, et al. Persistent gut microbiota immaturity in malnourished Bangladeshi children. *Nature.* 2014;510(7505):417-421.

45. Amoah AS, Obeng BB, Larbi IA, et al. Peanut-specific IgE antibodies in asymptomatic Ghanaian children possibly caused by carbohydrate determinant cross-reactivity. *J Allergy Clin Immunol.* 2013;132(3):639-647.

46. Wollmann E, Hamsten C, Sibanda E, et al. Natural clinical tolerance to peanut in African patients is caused by poor allergenic activity of peanut IgE. *Allergy.* 2015;70(6):638-652.

For peer review only

Table 1. Nutrient provision in IYCF and IYCF-plus trial arms

Red (fails to meet requirement), orange (meets >88% requirement), green (exceeds requirement).[1]

Nutrient	6-8 mo			9-11 mo			12-24 mo		
	Required from Complementary Food	IYCF	IYCF-PLUS	Required from Complementary Food	IYCF	IYCF-PLUS	Required from Complementary Food	IYCF	IYCF-PLUS
Energy (kcal)	363	118	222	464	118	247	560	118	272
Energy with mealie meal		268	372		368	497		468	622
Protein (g)	4.9	2.6	10.8	6.2	2.6	12.6	7.4	2.6	14.4
Fat (g)	0.0	9.6	15.7	4.3	9.6	15.8	8.3	9.6	16.0
Vitamin A (µg RE)	207	400	519	222	400	540	238	400	560
Folic Acid (µg)	48	80	132	50	80	160	133	80	188
Calcium (mg)	232	280	375	246	280	438	360	280	502
Iron (mg)	9.2	6.0	9.0	9.2	6.0	10.4	5.7	6.0	11.9
Zinc (mg)	3.8	8.0	9.1	3.9	8.0	9.5	3.8	8.0	9.8
Choline (mg)	47	13	145	55	13	152	114	13	158

[1]Nutrient requirements from complementary food calculated as total requirement less provided in breastmilk. Estimated mealie meal consumption is 150 kcal, 250 kcal, and 350 kcal based on observed intakes during SHINE trial. IYCF diet includes SHINE IYCF behavior change modules + 20 g Nutributter per day. IYCF-PLUS diet includes IYCF behaviour change modules + 20 g Nutributter per day + whole egg powder, moringa powder, and iron and zinc-fortified sugar bean powder.

For peer review only

For peer review only - http://bmjopen.bmj.com/site/about/guidelines.xhtml

Table 2 – Trial outcomes at 9 months of age (window 9-11 months)

Endpoint	Definition
Energy intake	Percentage of infants meeting daily energy requirements at 9 months of age, measured by multi-pass 24-hour dietary recall, as described in section 16.3.1
Protein, iron, zinc and folate intake	Percentage of infants meeting daily protein, iron, zinc and folate requirements at 9 months of age, measured by multi-pass 24-hour dietary recall, as described in section 16.3.1
Length-for-age Z-score	Length-for-age expressed as a Z-score compared to the WHO 2006 reference median
Weight-for-age Z score	Weight-for-age expressed as a Z-score compared to the WHO 2006 reference median
Weight-for-length Z score	Weight-for-length expressed as a Z-score compared to the WHO 2006 reference median
Haemoglobin	Concentration of haemoglobin (in g/dL) in a whole blood finger prick sample, measured by HemoCue point-of-care assay and adjusted for altitude
Microbiome maturity	Microbiota-for-age Z-score
Environmental enteric dysfunction (EED)	Biomarkers of intestinal inflammation (faecal neopterin and myeloperoxidase), small intestinal damage (plasma I-FABP and citrulline), intestinal permeability (faecal A1AT), microbial translocation (plasma sCD14, LBP), systemic inflammation (plasma CRP, AGP, TNF-alpha and K:T ratio) and growth hormone axis (IGF-1)
Innate immune cell phenotype	Surface marker expression by peripheral blood monocytes and neutrophils
Innate immune cell function	Surface marker expression and cytokine secretion from innate immune cells challenged with lipopolysaccharide *in vitro* relative to unstimulated controls<br><br>Capacity of innate immune cells to internalise bacteria *in vitro*
Plasma essential amino acids	Plasma concentrations of phenylalanine, valine, threonine, tryptophan, methionine, leucine, isoleucine, lysine and histidine, as measured by LC-MS-MS

For peer review only

Plasma choline	Plasma concentration of choline, as measured by LC-MS-MS
Urinary metabolic signature	Global untargeted metabolomic phenotyping undertaken by ^{1}H nuclear magnetic resonance spectroscopy

For peer review only

Table 3 – CHAIN trial schedule

**Procedure**	**Visit 1[a] Screening and enrolment**	**Randomisation and start of monthly IYCF or IYCF-plus interventions**	**Visit 2 Baseline**	**Visit 3[b] Endline**
Screening and eligibility check *Form 2*	x			
Informed consent *Form 3 and 4*	x			
Locator and contact information *Form 5*	x		x	x
Baseline interview *Form 6*			x	
Maternal weight, height and MUAC			x	
Infant weight, height, MUAC and head circumference			x	x
Infant blood collection[c]			x	x
Infant haemoglobin			x	x
Infant stool collection[c]			x	x
Infant urine collection[c]			x	x
Endline interview *Form 9*				x
24-hour dietary recall[d] *Form 10*				x
Informed consent for qualitative sub-studies [e] *Form 11*				
Qualitative sub-study 1 guide *Form 12a*				x

For peer review only

Informed consent for qualitative sub studies *Form 11* [f]				
Qualitative sub-study 2 guide *Form 12b*				x

[a]For mothers who wish to provide informed consent on a subsequent day to screening, these visits will be separated.
[b]Target date 9 months of infant age (visit window 9-11 months or 274-334 days)
[c]If any specimens cannot be collected during the visit (e.g. if the infant fails to pass stool), the specimen collection will be rescheduled for the next day, or as soon as possible after the visit. Rarely it may be necessary to repeat a specimen collection if the sample was insufficient, or fails quality control checks during processing in the laboratory. All blood draws will be kept to a safe limit, defined as maximum of 1ml/kg body weight.
[d]The primary outcome, measured by multi-pass 24-hour dietary recall, will be repeated 1 week later in a sub-sample of 50% infants for methods validation.
[e]Sub-group of up to 20 purposively selected households. The social scientist will visit the family during a separate visit at 7-9 months (214-273 days) of infant age, and obtain separate written informed consent for the qualitative interviews.
[f]Sub-group of up to 30 purposively selected households. The social scientist will obtain written informed consent for the qualitative interviews regarding migration, which will be conducted throughout the course of the study.

For peer review only

Table 4 – Module delivery

**Infant age (window period)[a]**	**Home Visit**	**Infant age (window period)[a]**	**CONTROL ARM IYCF modules and supplies**	**INTERVENTION ARM IYCF-plus modules and supplies**
5 months (5 months- <6 months)	1	5 months (5 months- <6 months) (153 – 182 days)	MODULE 1<br>• Nutrition for your baby<br>• Composition and functions of SQ-LNS [Feeding as a chore or actual work; stomach capacity and graph on nutrient gap of breastmilk and mealie meal]	MODULE 1<br>• Nutrition for your baby + introduction of the 3 powders<br>• Composition and functions of SQ-LNS and the 3 powders [Feeding as chore or actual work; stomach capacity and graph on nutrient gap of breastmilk and mealie meal)
6 months (6 months- <7 months)	2	6 months (6 months-<7 months) (183 – 213 days)	MODULE 2<br>• Introducing solid foods<br>• Additional information on SQ-LNS<br>• Breastmilk + porridge + SQ-LNS	MODULE 2<br>• Introducing solid foods<br>• Additional information on SQ-LNS and the 3 foods *(Bean powder, Egg powder and Moringa powder)*<br>• Breastmilk + porridge + SQ-LNS
6 months 1 week (6 months 1 week- <7 months)	3	6 months 1 week (6 months 1 week- <7 months) (190 - 213 days)	MODULE 2.1<br>• Frequency of complementary foods<br>• Reinforce messages<br>• Breastmilk + porridge + SQ-LNS	MODULE 2.1<br>• Introduce Bean Powder<br>• Frequency of complementary foods<br>• Reinforce messages<br>• Breastmilk + porridge + SQ-LNS + bean powder
6 months 2 weeks (6 months 2 weeks- <7 months)	4	6 months 2 weeks (6 months 2 weeks- <7 months) (197 – 213 days)	MODULE 2.2<br>• Nutrient-dense complementary meals<br>• Reinforce messages<br>• Breastmilk + porridge+ SQ-LNS + nutrient density	MODULE 2.2<br>• Introduce Egg Powder<br>• Nutrient-dense complementary meals<br>• Reinforce messages<br>• Breastmilk +porridge+ SQ-LNS + bean powder + egg powder
6 months 3 weeks (6 months 3 weeks- <7 months)	5	6 months 3 weeks (6 months 3 weeks- <7 months) (204 – 213 days)	MODULE 2.3<br>• Complementary feeding schedule and family support<br>• Reinforce messages<br>• Breastmilk + porridge + SQ-LNS	MODULE 2.3<br>• Introduce Moringa<br>• Complementary feeding schedule and family support<br>• Reinforce messages<br>• Breastmilk + porridge + SQ-LNS + bean powder + egg powder + moringa powder
7 months (7 months- <8 months)	6	7 months (7 months- <8 months) (214 – 243 days)	MODULE 3<br>• Introducing more foods<br>• Reinforce messages<br>• Breastmilk + SQ-LNS + porridge (sadza/soup/potatoes/maheu+ other locally available foods e.g. vegetables etc.)	MODULE 3<br>• Introducing more foods<br>• Reinforce messages<br>• Breastmilk + SQ-LNS + bean powder + egg powder + moringa powder + porridge (sadza /soup/potatoes/maheu + other locally available foods e.g. vegetables etc.)
8 months (8 months- <9 months)	7	8 months (8 months- <9 months) (244 – 273 days)	MODULE 4<br>• Feeding during illness<br>• Reinforce messages<br>• Breastmilk + SQ-LNS + porridge (sadza/soup/potatoes/maheu+	MODULE 4<br>• Feeding during illness<br>• Reinforce messages<br>• Breastmilk + SQ-LNS + bean powder + egg powder + moringa powder +

			other locally available foods e.g. vegetables etc.)	porridge (sadza /soup/potatoes/maheu + other locally available foods e.g. vegetables etc.)
9 months (9 months- <10 months)	8	9 months (9 months- <10 months) (274 – 304 days)	MODULE 5<br>• Dietary Diversity<br>• Reinforce messages<br>• Dietary diversity<br>• Breastmilk + SQ-LNS + porridge (sadza/soup/potatoes/maheu+ other locally available foods e.g. vegetables etc.)	MODULE 5<br>• Dietary Diversity<br>• Reinforce messages<br>• Dietary diversity<br>• Breastmilk + SQ-LNS + bean powder + egg powder + moringa powder + porridge (sadza /soup/potatoes/maheu + other locally available foods e.g. vegetables etc.)
10 months (10 months- <11 months)	9	10 months (10 months- <11 months) (305 – 334 days)	MODULE 6<br>• Reinforcing all Modules<br>• Breastmilk + SQ-LNS + porridge (sadza/soup/potatoes/maheu+ other locally available foods e.g. vegetables etc.)	MODULE 6<br>• Reinforcing all Modules<br>• Breastmilk + SQ-LNS + bean powder + egg powder + moringa powder + porridge (sadza /soup/potatoes/maheu + other locally available foods e.g. vegetables etc.)
11 months (11 months- <12 months)	10	11 months (11 months- <12 months) (345 – 365 days)	MODULE 6<br>• Reinforcing all Modules<br>• Breastmilk + SQ-LNS + porridge (sadza/soup/potatoes/maheu+ other locally available foods e.g. vegetables etc.)	MODULE 6<br>• Reinforcing all Modules<br>• Breastmilk + SQ-LNS + bean powder + egg powder + moringa powder + porridge (sadza /soup/potatoes/maheu + other locally available foods e.g. vegetables etc.)

[a] If a module is not delivered within the intervention window (i.e. appropriate infant age), the VHW will try to catch up by scheduling a new date as soon as possible. Each module will be delivered to the mother and her family. If the rescheduled module for modules 1.0 and 2.0 overlap, these two modules will be delivered at the same time. If the rescheduled module overlaps with the next visit for other modules (2.0, 2.1, 2.2, 2.3 etc) the visits will be scheduled at least 3 days apart so that families have time to absorb the new material. Delivery of IYCF-plus modules has therefore been designed to be flexible following complementary feeding guidance. Experience from formative work showed that it is feasible to deliver the combined modules at once.

For peer review only - http://bmjopen.bmj.com/site/about/guidelines.xhtml

Table 5: Quantities of food supplements in the IYCF-plus arm

Infant Age Group (months)	Mealie Meal (PVA maize)[1]	SQ-LNS[2]	Whole egg powder[3]	Moringa leaf, dried and ground[4]	Sugar bean legume, finely ground[5]
**6-8**	≥ 42 g (3 Tbsp)	20 g	14 g (3 tsp)	5 g (1 tsp)	5 g (1 tsp)
**9-11**	≥ 71 g (4.5 Tbsp)	20 g	14 g (3 tsp)	10 g (2 tsp)	10 g (2 tsp)

Food supplements will be delivered monthly by VHWs. [1]Mealie Meal (PVA maize) will be provided in 500g bags, with 3 bags/month between 6-8 months of age and 5 bags/month between 9-11 months of age. [2]SQ-LNS will be supplied monthly to ensure 1 x 20g sachet per day can be provided. [3]Whole egg powder will be delivered as a 500g bag per month. [4]Moringa leaf powder will be supplied in 175g bags, with 1 bag/month between 6-8 months of age and 2 bags/month between 9-11 months of age. [5]Sugar beans will be supplied in 175g bags, with 1 bag/month between 6-8 months of age and 2 bags/month between 9-11 months of age. These quantities allow for 15% extra in case of spillage or sharing

For peer review only

Table 6 – Laboratory analyses

Sample type	Assay	Method	Location of work	Study subjects	Time-points
Plasma	I-FABP, CRP, AGP, TNFα, LBP, sCD14, IGF-1	ELISA	Zvitambo	All	Baseline, endline
Peripheral blood leukocytes	Innate immune cell phenotype[a]	Flow cytometry	Zvitambo	All	Baseline, endline
Whole blood	Whole Blood Culture with and without LPS	Cell culture, flow cytometry and ELISA	Zvitambo	All	Baseline, endline
Whole blood	Bacterial binding Assay	Cell culture, flow cytometry	Zvitambo	All	Baseline, endline
Stool	Myeloperoxidase, neopterin, alpha-1 antitrypsin	ELISA	Zvitambo	All	Baseline, endline
Urine	Global untargeted metabolomic phenotyping	^{1}H NMR spectroscopy	Southampton, UK	All	Baseline, endline
Plasma	Global untargeted metabolomic phenotyping	^{1}H NMR spectroscopy	Southampton, UK	All	Baseline, endline
Plasma	Kynurenine:tryptophan ratio, citrulline, essential amino acids, choline	Ultrahigh-performance liquid chromatography tandem mass spectrometry with electrospray ionization	Southampton, UK	All	Baseline, endline
Stool	Whole metagenome shotgun sequencing	Illumina HiSeq	Blizard Institute, UK	All	Baseline, endline
Stool	Metabolic phenotyping of fecal water	^{1}H NMR spectroscopy	Southampton, UK	All	Baseline, endline
Urine	Aflatoxin M1 and creatinine	ELISA	Zvitambo	All	Baseline, endline

[a] Expression of surface activation markers HLA-DR, CD64 and CD16 on monocytes and HLA-DR, CD64, CD16 and CD62L on neutrophils

I-FABP: Intestinal fatty acid binding protein; AGP: Alpha-1 acid glycoprotein; CRP: C-reactive protein; LBP: lipopolysaccharide binding protein; IGF-1: insulin like growth factor 1

review only

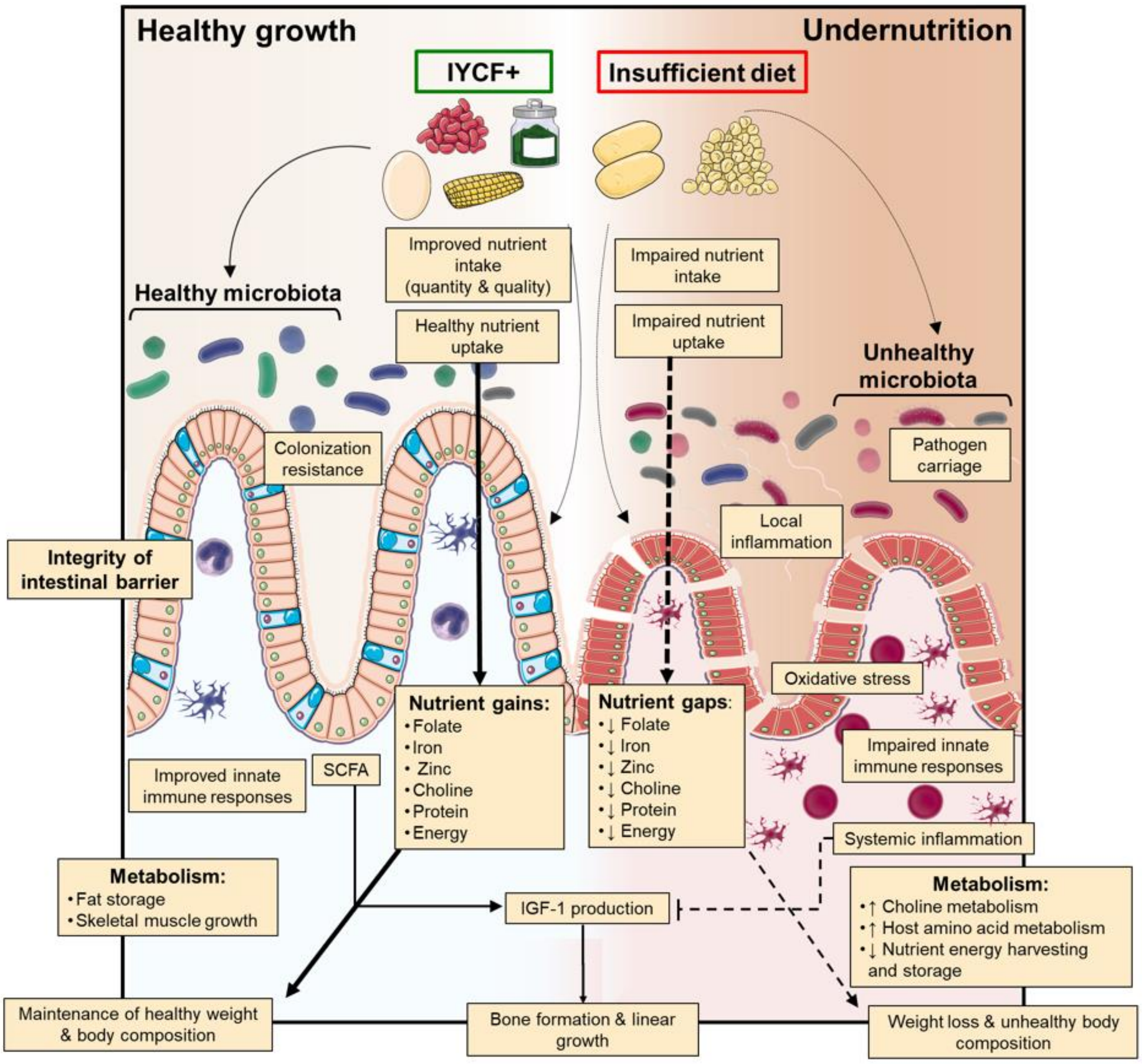

For peer review only

Articles

Independent and combined effects of improved water, sanitation, and hygiene, and improved complementary feeding, on child stunting and anaemia in rural Zimbabwe: a cluster-randomised trial

*Jean H Humphrey, Mduduzi N N Mbuya, Robert Ntozini, Lawrence H Moulton, Rebecca J Stoltzfus, Naume V Tavengwa, Kuda Mutasa, Florence Majo, Batsirai Mutasa, Goldberg Mangwadu, Cynthia M Chasokela, Ancikaria Chigumira, Bernard Chasekwa, Laura E Smith, James M Tielsch, Andrew D Jones, Amee R Manges, John A Maluccio, Andrew J Prendergast, for the Sanitation Hygiene Infant Nutrition Efficacy (SHINE) Trial Team**

Summary

Background Child stunting reduces survival and impairs neurodevelopment. We tested the independent and combined effects of improved water, sanitation, and hygiene (WASH), and improved infant and young child feeding (IYCF) on stunting and anaemia in in Zimbabwe.

Methods We did a cluster-randomised, community-based, 2×2 factorial trial in two rural districts in Zimbabwe. Clusters were defined as the catchment area of between one and four village health workers employed by the Zimbabwe Ministry of Health and Child Care. Women were eligible for inclusion if they permanently lived in clusters and were confirmed pregnant. Clusters were randomly assigned (1:1:1:1) to standard of care (52 clusters), IYCF (20 g of a small-quantity lipid-based nutrient supplement per day from age 6 to 18 months plus complementary feeding counselling; 53 clusters), WASH (construction of a ventilated improved pit latrine, provision of two handwashing stations, liquid soap, chlorine, and play space plus hygiene counselling; 53 clusters), or IYCF plus WASH (53 clusters). A constrained randomisation technique was used to achieve balance across the groups for 14 variables related to geography, demography, water access, and community-level sanitation coverage. Masking of participants and fieldworkers was not possible. The primary outcomes were infant length-for-age Z score and haemoglobin concentrations at 18 months of age among children born to mothers who were HIV negative during pregnancy. These outcomes were analysed in the intention-to-treat population. We estimated the effects of the interventions by comparing the two IYCF groups with the two non-IYCF groups and the two WASH groups with the two non-WASH groups, except for outcomes that had an important statistical interaction between the interventions. This trial is registered with ClinicalTrials.gov, number NCT01824940.

Findings Between Nov 22, 2012, and March 27, 2015, 5280 pregnant women were enrolled from 211 clusters. 3686 children born to HIV-negative mothers were assessed at age 18 months (884 in the standard of care group from 52 clusters, 893 in the IYCF group from 53 clusters, 918 in the WASH group from 53 clusters, and 991 in the IYCF plus WASH group from 51 clusters). In the IYCF intervention groups, the mean length-for-age Z score was 0·16 (95% CI 0·08–0·23) higher and the mean haemoglobin concentration was 2·03 g/L (1·28–2·79) higher than those in the non-IYCF intervention groups. The IYCF intervention reduced the number of stunted children from 620 (35%) of 1792 to 514 (27%) of 1879, and the number of children with anaemia from 245 (13·9%) of 1759 to 193 (10·5%) of 1845. The WASH intervention had no effect on either primary outcome. Neither intervention reduced the prevalence of diarrhoea at 12 or 18 months. No trial-related serious adverse events, and only three trial-related adverse events, were reported.

Interpretation Household-level elementary WASH interventions implemented in rural areas in low-income countries are unlikely to reduce stunting or anaemia and might not reduce diarrhoea. Implementation of these WASH interventions in combination with IYCF interventions is unlikely to reduce stunting or anaemia more than implementation of IYCF alone.

Funding Bill & Melinda Gates Foundation, UK Department for International Development, Wellcome Trust, Swiss Development Cooperation, UNICEF, and US National Institutes of Health.

Copyright © 2018 The Author(s). Published by Elsevier Ltd. This is an Open Access article under the CC BY 4.0 license.

Lancet Glob Health 2019; 7: e132–47

See **Comment** page e16

*Members of the SHINE Trial team are listed at https://doi.org/10.1093/cid/civ844

Department of International Health, Johns Hopkins Bloomberg School of Public Health, Baltimore MD, USA (Prof J H Humphrey ScD, M N N Mbuya PhD, Prof L H Moulton PhD, Prof A J Prendergast DPhil); **Zvitambo Institute for Maternal and Child Health Research, Harare, Zimbabwe** (Prof J H Humphrey, M N N Mbuya, R Ntozini, N V Tavengwa MSW, K Mutasa MPH, F Majo RGN, B Mutasa MBA, B Chasekwa MSc, L E Smith PhD, Prof A J Prendergast); **Division of Nutritional Sciences, Cornell University, Ithaca, NY, USA** (M N N Mbuya, R J Stoltzfus PhD); **Global Alliance for Improved Nutrition, Washington, DC, USA** (M N N Mbuya); **Ministry of Health and Child Care, Harare, Zimbabwe** (G Mangwadu MSc, C M Chasokela PhD, A Chigumira MSc); **Department of Epidemiology and Environmental Health, School of Public Health and Health Professions, University at Buffalo, Buffalo, NY, USA** (L E Smith); **Department of Global Health, Milken Institute School of Public Health, George Washington University, Washington, DC, USA** (Prof J M Tielsch PhD); **Department of Nutritional Sciences, School of Public Health, University of Michigan, Ann Arbor, MI, USA** (A D Jones PhD); **University of British Columbia, Vancouver,**

Introduction

Globally, linear growth faltering (ie, stunting) is the most prevalent form of undernutrition.[1,2] Stunting largely occurs between conception and age 24 months, when mean length-for-age Z scores among Asian and African children plummet to –2·0, with little change thereafter.[1,2] Stunting reduces child survival, educational attainment, and adult economic productivity.[1,2] Furthermore, the offspring of adults who were stunted as children are at increased risk of stunting, creating an intergenerational

For peer review only - http://bmjopen.bmj.com/site/about/guidelines.xhtml

BC, Canada (A R Manges PhD); **Middlebury College, Middlebury, VT, USA** (Prof J A Maluccio PhD); and **Blizard Institute, Queen Mary University of London, London, UK** (Prof A J Prendergast)

Correspondence to: Prof Jean H Humphrey, Department of International Health, Johns Hopkins Bloomberg School of Public Health, 615 North Wolfe Street, Baltimore, MD 21205, USA **jhumphrey@zvitambo.co.zw**

Research in context

Evidence before this study

Before this trial, a Review done for *The Lancet* Nutrition Series highlighted that child stunting is a highly prevalent condition with adverse short-term and long-term sequelae. A systematic review of the literature showed that complementary feeding studies of improvements to the quality or quantity of infant diet could improve linear growth, but only moderately. That review was followed by an updated systematic review published just after our trial was completed; it showed that complementary feeding had an effect of 0·11 on length-for-age Z scores in food-secure populations, which is about 5–10% of the deficit experienced by Asian and African children. Finally, in seminal research done in The Gambia, child linear growth failure was strongly associated with indicators of environmental enteric dysfunction—increased gut permeability and systemic inflammation resulting from translocation of gut microbes. Although untested, the cause of environmental enteric dysfunction has been widely attributed to faecal–oral exposure resulting from living in conditions of poor water, sanitation, and hygiene (WASH). Before this trial, a meta-analysis of data from Demographic and Health Surveys for low-income and middle-income countries showed an association between linear growth and sanitation, but no randomised trials had been published in which the effect of sanitation on any child health outcome, including diarrhoea, had been tested. Additionally, many trials of handwashing with soap and chlorination of drinking water showed reductions in diarrhoea, but none reported the effects of these interventions on gut health or child growth. Since we began this trial, four published trials have assessed the effect of community-based sanitation on stunting. In two of these trials, both done in India, uptake of sanitation was low, the frequency of open defecation remained high, and no benefits for child health were reported in either. In a trial done in Indonesia, intervention uptake was modest. The intervention reduced diarrhoea but had no effect on linear growth. Finally, in a trial done in Mali, the intervention nearly doubled latrine coverage and substantially reduced the frequency of open defecation. It had no effect on diarrhoea, but increased length-for-age Z scores by 0·18. Most recently, the WASH Benefits trials in Bangladesh and Kenya tested the effect of six interventions (water treatment, handwashing, sanitation, all three WASH interventions together, infant feeding, and infant feeding plus all three WASH interventions) on linear growth and diarrhoea. In both trials, modest reductions in stunting were noted in the infant feeding group and the infant feeding plus WASH group, but no effects on linear growth were noted in any of the WASH-only group. Diarrhoea was reduced in all active groups in the Bangladesh trial except in the water treatment only group. None of the interventions reduced diarrhoea in the Kenya trial.

Added value of this study

The Sanitation Hygiene Infant Nutrition Efficacy (SHINE) trial was a 2 × 2 factorial cluster-randomised trial to test the independent and combined effects of improving infant diet and household WASH on length-for-age Z score and haemoglobin (primary outcomes) at 18 months of age. The WASH intervention included a play space to minimise geophagia and ingestion of chicken faeces by children in addition to conventional WASH interventions (sanitation, water treatment, handwashing, and hygienic preparation of food). The infant and young child feeding intervention included 20 g of a small-quantity lipid-based nutrient supplement per day from 6 to 18 months in addition to counselling that targeted key barriers to optimal infant feeding in this context. Intervention intensity (ie, frequency of contact between behaviour-change promoters and participants) was monthly. The IYCF intervention increased the mean length-for-age Z score by 0·16 (95% CI 0·08–0·23) and the mean haemoglobin concentration by 2·0 g/L (1·3–2·8), reduced stunting and anaemia, and improved ponderal growth compared with the non-IYCF interventions. The WASH intervention had no effect on these outcomes. Neither intervention reduced child diarrhoea or mortality.

Implications of all the available evidence

Our trial provides further high-level evidence that elementary WASH interventions (ie, provision of point-of-use water chlorination, handwashing stations not connected to water supply, and improved pit latrines, with promotion of hygiene behaviours) delivered at the household level in rural areas of low-income countries are unlikely to reduce stunting and might not reduce diarrhoea. Implementation of these WASH interventions together with IYCF interventions will not reduce stunting more than implementation of IYCF alone.

cycle of low human capital.[1,2] Stunting has been largely intractable to targeted interventions. On average, complementary feeding interventions increase length-for-age Z scores by 0·1,[3] and elimination of all diarrhoea in the first 2 years of life is estimated to increase length by 0·38 cm (or length-for-age Z scores by 0·13).[4] Similar to stunting, childhood anaemia is prevalent among children younger than 2 years in Africa and Asia, and is a primary cause of cognitive delay.[2] Increasing dietary iron intake reduces anaemia by 32–62%, which leaves a substantial proportion of disease unaddressed.[2]

The UNICEF framework for undernutrition has been a guiding document for nearly 30 years.[5] It highlights inadequate dietary intake and disease as the immediate causes of child undernutrition, and specifies that a multisectoral approach that addresses both proximal and distal determinants is required. Based on this framework, integration of improved infant diets with improved water, sanitation, and hygiene (WASH) is a logical approach, because of the role of WASH in reducing morbidity, especially diarrhoea. Along with others,[6] we[7] further hypothesised that the adverse

For peer review only - http://bmjopen.bmj.com/site/about/guidelines.xhtml

effects of poor WASH on growth are primarily mediated through environmental enteric dysfunction, a subclinical condition of the small intestine characterised by blunted villi, intestinal inflammation, and intestinal permeability.[7] Environmental enteric dysfunction reduces nutrient absorption, triggers chronic systemic inflammation, and is seemingly ubiquitous among people living in impoverished conditions in low-income and middle-income countries. We further hypothesised that a household WASH intervention targeting pathways of faecal–oral exposure in young children will reduce environmental enteric dysfunction, increase linear growth, and reduce anaemia by facilitating iron mobilisation and erythropoiesis. Finally, we hypothesised that the beneficial effects of WASH on growth and anaemia will be additive to those of improving infant diets.[2] The Sanitation, Hygiene, Infant Nutrition Efficacy (SHINE) trial was designed to test these hypotheses.

Methods

Study design and participants

The design and methods of SHINE have been reported previously,[2] and the protocol and statistical analysis plan are available online. Briefly, SHINE was a cluster-randomised, community-based 2×2 factorial trial in two contiguous rural districts in Zimbabwe (Chirumanzu and Shurugwi). The districts have a 15% prevalence of antenatal HIV[8] and a high prevalence of schistosomiasis, but a very low prevalence of soil-transmitted helminths.[9] Rotavirus vaccination was introduced during the trial from May, 2014. Households are usually single-family dwellings surrounded by farm land. Before the trial, mean distance between households was 82·6 m,[10] and population density was 18·6 people per km². Clusters were defined as the catchment area of between one and four village health workers (VHWs) employed by the Zimbabwe Ministry of Health and Child Care. Urban and uninhabited areas were excluded. Between Nov 22, 2012 and March 27, 2015, VHWs prospectively surveyed new pregnancies, established the date of last menstrual period, and referred pregnant women to SHINE research nurses, who enrolled eligible women. Women were eligible for inclusion if they permanently resided in a study cluster and were confirmed pregnant. During the recruitment period, the cutoff of gestational age for eligibility was gradually loosened (from 14 weeks' gestational age to just before parturition) to maximise recruitment (appendix). The Medical Research Council of Zimbabwe and the Institutional Review Board of the Johns Hopkins Bloomberg School of Public Health approved the study protocol. All participants provided written informed consent.

Randomisation and masking

Clusters were allocated (1:1:1:1) to one of four treatment groups: standard of care, infant and young child feeding (IYCF), WASH, or IYCF plus WASH. LHM, the study's senior statistician, used a constrained randomisation technique[11] to identify 5000 allocation schemes that achieved balance across the groups for 14 variables related to geography, demography, water access, and sanitation coverage, and also met bias and validity specifications (appendix). From these, ten allocations were randomly selected. The final allocation was selected at a public randomisation event attended by elected representatives of the study districts. Masking of participants and fieldworkers was not possible because of the obvious visual differences between interventions, but investigators were blinded to treatment groups until the final analysis of each prespecified outcome.

Procedures

Interventions were informed by extensive formative research and piloting.[12–15] Behaviour-change modules were delivered by group-specific VHWs, who underwent training for 20 days to deliver standard of care, 30 days to deliver WASH, 32 days to deliver IYCF, and 35 days to deliver IYCF plus WASH. All enrolled women were scheduled to receive 15 behaviour-change modules with specific messages and interactive tools from enrolment until 12 months after the birth of their children (roughly one visit per month). Other family members were also encouraged to participate. A sequential integrated longitudinal intervention was delivered, and at each visit, previous information was reviewed before new information was introduced. Previously missed modules were delivered before any new material. Between 13 months and 17 months postnatal, VHWs continued monthly visits providing routine care. During these visits, VHWs informally encouraged participants to practise behaviours relevant to their treatment group, although formal modules were not implemented. At 18 months, a review module was implemented in all treatment groups before the mother completed the trial.

In the standard-of-care group, VHWs promoted exclusive breastfeeding to 6 months,[16] advised on neonatal care, and promoted uptake of Ministry of Health and Child Care services, including antenatal care, immunisations, and family planning. In the WASH group, VHWs delivered all the standard-of-care messages, plus information about safe disposal of faeces, handwashing with soap at key times, protection of infants from geophagia and ingestion of animal faeces, chlorination of drinking water (especially for infants), and hygienic preparation of complementary food. Additionally, ventilated improved pit latrines were constructed and two handwashing stations were installed by community builders supervised by Ministry of Health and Child Care environmental health technicians within 6 weeks of enrolment. A plastic mat and play yard (North States, Minneapolis, MN, USA) were delivered to the home by a trial logistician at 2 and 6 months postnatal, respectively, and VHWs made monthly deliveries of soap

For the **protocol and statistical analysis plan** see https://osf.io/w93hy

See Online for appendix

For peer review only - http://bmjopen.bmj.com/site/about/guidelines.xhtml

from the time of the handwashing module (roughly 30 weeks antenatal) and chlorine from the time of the water treatment module (4 months postnatal) until 18 months postnatal. In the IYCF group, VHWs delivered all the standard-of-care messages plus information about the importance of nutrition for infant health, growth, and development; feeding nutrient-dense food and 20 g of a small-quantity lipid-based nutrient supplement[17] (Nutriset, Malaumay, France) daily from age 6–18 months; processing foods to facilitate mastication and swallowing; feeding during illness; and dietary diversity. Participants also received monthly deliveries of 30 sachets of the small-quantity lipid-based nutrient supplement from VHWs from infant age 6 months to 18 months postnatal. In the combined group, participants received all standard-of-care, WASH, and IYCF interventions. Ventilated improved pit latrines were built for participants in the standard-of-care and IYCF groups after trial completion.

Research nurses made home visits at baseline (roughly 2 weeks after mothers provided consent), 32 weeks' gestation, and 1, 3, 6, 12, and 18 months post partum to assess maternal and household characteristics and trial outcomes. Intervention uptake was assessed at all visits and reported here as prespecified for the 12-month postnatal visit. At baseline, mothers' height, weight, mid-upper-arm circumference, and haemoglobin concentrations (with Hemocue, Ängelholm, Sweden) were measured. They were also tested for *Schistosoma haematobium* (by urinary microscopy) and HIV (via the rapid test algorithm). HIV-positive women were urged to seek immediate antenatal care for prevention of mother-to-child transmission. Other maternal and household characteristics assessed included dietary diversity, food insecurity, household wealth, and maternal capabilities.[18]

Infant birth date, weight, and delivery details were transcribed from health facility records. We provided Tanita BD-590 (Arlington Heights, IL, USA) infant scales to all health institutions in the study area and trained facility staff in use of the scales. Gestational age at delivery was calculated from the date of the mother's last menstrual period. Infant weight, length, head circumference, and mid-upper-arm circumference were measured at every postnatal visit (appendix). At the 18-month postnatal visit (ie, the trial endpoint), haemoglobin concentrations were measured, and length was calculated as the median of three measurements. Nurses were standardised against a gold standard anthropometrist by measuring non-study children from the community during a quality-control session held every 6 months. Infant diarrhoea (three or more loose or watery stools in 24 h), dysentery (stool with blood or mucus), and acute respiratory infection (fast or difficult breathing) were assessed by 7-day maternal recall at postnatal visits. Infants with acute malnutrition or illness were referred to clinics. At the 18-month postnatal visit (ie, the trial endpoint), mothers and infants were visited anywhere in the country. However, in view of the household-based nature of interventions, intermediate visits were done only when the mother was available in the household where she consented.

Serious adverse events and adverse events were ascertained during data collection visits and by VHWs, and were referred to a senior research nurse who collected details. Events were reviewed by the study physician (AJP) to establish relatedness to trial interventions before reporting them to responsible institutional review boards. An independent data safety and monitoring board comprising two physicians from Zimbabwe and a statistician from the UK reviewed interim adverse event data.

Outcomes

We prespecified that primary trial inferences would be based on findings among infants born to mothers who were HIV negative during pregnancy. Primary outcomes were mean length-for-age Z score and haemoglobin concentration at 18 months (target age 76–80 weeks, allowable range 76–130 weeks) in all enrolled participants (intention-to-treat analysis). Secondary outcomes were mean weight-for-age Z scores, weight-for-length Z scores, mid-upper-arm circumference-for-age Z scores, and head circumference-for-age Z scores; the proportion of infants who were stunted (ie, length-for-age Z score less than –2), severely stunted (ie, length-for-age Z score less than –3), anaemic (ie, haemoglobin concentration <105 g/L), severely anaemic (ie, haemoglobin concentration <70 g/L), underweight (ie, weight-for-age Z scores less than –2), and wasted (ie, weight-for-height Z scores less than –2); mean prevalence of diarrhoea, dysentery, and acute respiratory infection based on 7-day maternal history at infant age 12 months and 18 months; and cumulative mortality up to age 18 months.

Statistical analysis

The original sample size calculation was 4800 women to allow for 15% exclusion because of maternal HIV and 20% loss because of fetal and infant deaths, withdrawal, and loss to follow-up. Actual recruitment was 10% higher than the sample size calculation to ensure sufficient power for sensitivity analyses. With a minimum of 816 HIV-unexposed infants per group at 18 months, the trial was powered to detect a 0·2 difference in length-for-age Z scores, a reduction of 8 percentage points in stunting, and a 2·6 g/L shift in haemoglobin for the marginal effect of either intervention, with 90% power and type 1 error of 5%.[2] This calculation was based on an assumed coefficient of variation of the true proportions of 0·43, and an effective loss of 33% of sample size because of variability in cluster size.

All analyses were done on an intention-to-treat basis at the child level. For primary analyses, we used generalised estimating equations that accounted for within-cluster correlation and contained two dummy variables

For peer review only - http://bmjopen.bmj.com/site/about/guidelines.xhtml

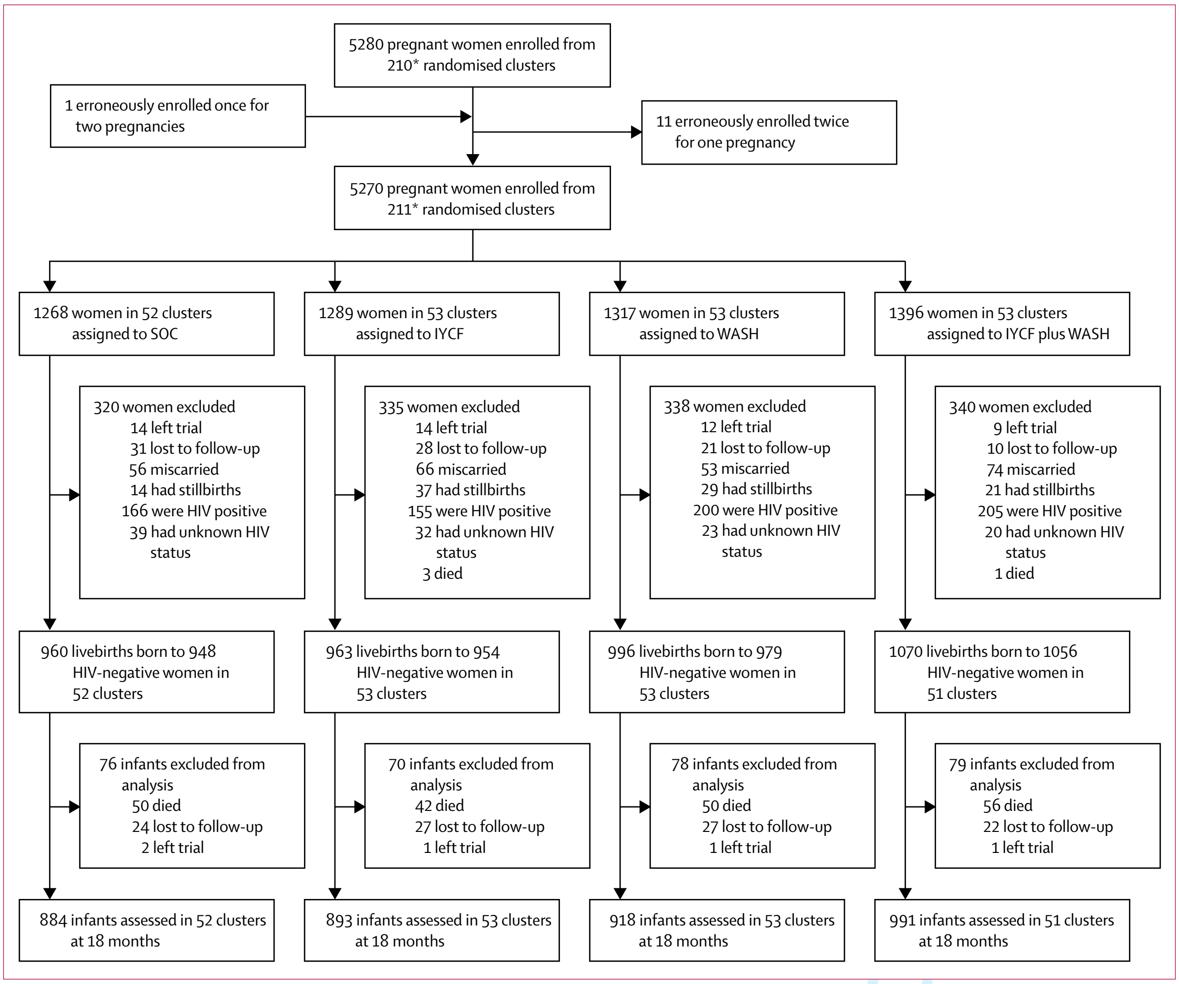

***Figure:* Trial profile**

SOC=standard of care. IYCF=infant and young child feeding. WASH=water, sanitation, and hygiene. *212 clusters were randomly assigned, 53 in each of the four trial groups. After randomisation, one cluster was excluded because it was in an urban area, one was excluded because the village health workers covering it mainly had clients outside the study area, and two more were merged on the basis of subsequent data for village health worker coverage. Three new cluster designations were created because of anomalies in the original mapping. For two of these clusters, the trial group was clear; the third contained areas that were in two trial groups, and was assigned to the under-represented group, resulting in 53 clusters in each group. All these changes occurred before enrolment began. When enrolment was completed, however, no women were enrolled in one cluster in the SOC group and thus 211 clusters were available for analysis.

representing the main effect of the IYCF intervention (the two IYCF groups compared with the two non-IYCF groups) and the WASH intervention (the two WASH groups compared with the two non-WASH groups), unadjusted for other covariates, with an exchangeable working correlation structure.[11] Although the study was not powered to detect a statistical interaction between the two interventions, we estimated these interactions for each outcome. When the interaction was significant (ie, $p<0\cdot05$ according to the Wald test) or had a sizeable point estimate (ie, relative risk [RR] >2 or <0·5 when comparing ratio-of-ratios, or difference-of-differences >0·25 SDs when comparing continuous outcomes), results were based on a regression model with three dummy variables to represent IYCF, WASH, and IYCF plus WASH compared with standard of care instead of the model of two terms. In adjusted analyses we controlled for prespecified baseline covariates, which were initially assessed in bivariate analyses to identify those with an important association with the outcome (ie, $p<0\cdot2$ or RR >2·0 or <0·5 for dichotomous outcomes, and $p<0\cdot2$ or difference >0·25 SDs for continuous outcomes). Selected covariates were entered in a multivariable regression model; a forward stepwise selection procedure was implemented with $p<0\cdot2$ to enter. A log-binomial specification was used to facilitate estimation of RRs. Depending on the analysis, other methods for comparison of groups while accounting for within-cluster correlation included multinomial and ordinal regression models with robust variance estimation, and Somers' D for medians.

In a per-protocol analysis, we examined the effect of the interventions when behaviour-change modules were delivered at high fidelity (which was predefined

For peer review only - http://bmjopen.bmj.com/site/about/guidelines.xhtml

	Standard of care	Infant and young child feeding	Water, sanitation, and hygiene	Water, sanitation, and hygiene plus infant and young child feeding
Mothers	948	954	979	1056
Infants	960	963	996	1070
Mothers completing baseline visit	866	867	943	1013
Household characteristics				
Median number of occupants (IQR)	5 (3–6)	5 (4–6)	5 (3–6)	5 (3–6)
Wealth quintile[19]				
1 (lowest)	189/858 (22%)	132/861 (15%)	189/935 (20%)	170/1005 (17%)
2	163/858 (19%)	156/861 (18%)	181/935 (19%)	206/1005 (20%)
3	171/858 (20%)	187/861 (22%)	178/935 (19%)	207/1005 (21%)
4	169/858 (20%)	188/861 (22%)	202/935 (22%)	208/1005 (21%)
5 (highest)	166/858 (19%)	198/861 (23%)	185/935 (20%)	214/1005 (21%)
Electricity				
Power grid	26/855 (3%)	33/857 (4%)	26/938 (3%)	15/1006 (1%)
Other power source				
Solar	548/855 (64%)	584/858 (68%)	653/938 (70%)	698/1006 (69%)
Generator	22/855 (3%)	34/858 (4%)	31/938 (3%)	31/1006 (3%)
No electricity	285/855 (33%)	240/858 (28%)	254/938 (27%)	277/1006 (28%)
Sanitation				
Household members defecate in the open	1924/3602 (53%)	1854/3900 (48%)	1916/4009 (48%)	1988/4354 (46%)
Any latrine at household	280/852 (33%)	346/850 (41%)	331/917 (36%)	367/987 (37%)
Improved latrine at household	245/852 (29%)	301/849 (35%)	293/914 (32%)	318/986 (32%)
Improved latrine with well trodden path not shared with other households and not used for storage	178/829 (21%)	233/817 (29%)	229/886 (26%)	250/952 (26%)
Water				
Main source of household drinking water improved	529/855 (62%)	558/854 (65%)	567/923 (61%)	639/993 (64%)
Treated drinking water to make safer	118/840 (14%)	108/836 (13%)	109/912 (12%)	114/980 (12%)
Median one-way walk time to fetch water (IQR), min	10 (5–20)	10 (5–18)	10 (5–20)	10 (5–20)
Mean water volume collected per person in past 24 h (SD), L	9·5 (10·5)	9·6 (8·6)	9·9 (16·6)	9·6 (10·0)
Hygiene				
Handwashing station at household	37/796 (5%)	20/812 (2%)	121/885 (14%)	130/940 (14%)
Handwashing station with water and rubbing agent	10/793 (1%)	1/812 (<1%)	8/884 (1%)	10/938 (1%)
Improved floor*	442/845 (52%)	481/851 (57%)	516/921 (56%)	557/990 (56%)
Median number of chickens (IQR)	6 (2–10)	7 (2–12)	6 (2–10)	5 (2–10)
Livestock observed inside home	324/859 (38%)	345/861 (40%)	341/931 (37%)	363/1003 (36%)
Faeces observed in the yard	271/855 (32%)	323/857 (38%)	274/928 (30%)	268/995 (27%)
Diet quality and food security				
Household meets minimum Diet Diversity Score[20]	292/769 (38%)	326/744 (44%)	323/833 (39%)	353/878 (40%)
Median Coping Strategies Index score[21] (IQR)	1 (0–7)	0 (0–6)	1 (0–7)	1 (0–7)

(Table 1 continues on next page)

for the IYCF plus WASH group as receiving ten core modules and for the other study groups as receiving all modules scheduled at the same timepoints when IYCF plus WASH core modules were delivered). Several sensitivity analyses were prespecified: exclusion of mothers enrolled before Nov 1, 2013, to account for initial delays in latrine construction; exclusion of children born to women who were HIV negative during pregnancy but HIV positive at 18 months; and restriction of analyses to children in whom primary outcomes were measured during tight infant age windows (ie, at age 76–80 weeks and 76–100 weeks). A prespecified subgroup analysis of primary outcomes by infant sex was planned if the interaction terms were significant.

We used Stata (version 14.1) for all analyses. This trial is registered with ClinicalTrials.gov, number NCT01824940.

Role of the funding source

The study funders approved the trial design, but had no roles in data collection, analysis, or interpretation, or writing of the report. The corresponding author had full access to all study data and had final responsibility for the decision to submit for publication.

For peer review only - http://bmjopen.bmj.com/site/about/guidelines.xhtml

	Standard of care	Infant and young child feeding	Water, sanitation, and hygiene	Water, sanitation, and hygiene plus infant and young child feeding
(Continued from previous page)				
Maternal characteristics				
Mean age (SD), years	25·4 (8·5)	25·7 (7·6)	25·6 (8·5)	25·9 (8·1)
Mean height (SD), cm	156·0 (6·1)	160·1 (5·9)	159·5 (8·0)	159·6 (10·2)
Mean mid-upper-arm circumference (SD), cm	26·3 (3·2)	26·5 (3·2)	26·4 (3·6)	26·5 (3·3)
Positive microscopy for *Schistosoma haematobium*	78/824 (9%)	80/820 (10%)	116/878 (13%)	104/958 (11%)
Mean years of schooling completed (SD)	9·6 (2·2)	9·7 (2·8)	9·5 (2·0)	9·6 (2·5)
Median parity (IQR)	2 (1–3)	2 (1–3)	2 (1–3)	2 (1–3)
Married	850/894 (95%)	852/892 (96%)	886/933 (95%)	958/998 (10%)
Employed	53/857 (6%)	81/857 (9%)	91/936 (10%)	86/1006 (9%)
Religion				
Apostolic	459/899 (51%)	395/903 (44%)	440/939 (47%)	469/1004 (47%)
Other Christian	383/899 (43%)	444/903 (49%)	410/939 (44%)	447/1004 (45%)
Other	57/899 (6%)	64/903 (7%)	89/939 (9%)	88/1004 (9%)
Infant characteristics				
Female sex	474/959 (49%)	472/958 (49%)	487/995 (49%)	529/1062 (50%)
Mean birthweight (SD), kg	3·1 (0·6)	3·10 (0·49)	3·09 (0·54)	3·10 (0·50)
Birthweight <2500 g	79/855 (9%)	76/864 (9%)	86/892 (10%)	84/962 (9%)
Institutional delivery	752/855 (88%)	761/861 (88%)	794/892 (89%)	854/948 (90%)
Vaginal delivery	814/874 (93%)	812/870 (93%)	833/904 (92%)	897/967 (93%)

Baseline variables are presented for mothers who had livebirths. Maternal and household data were collected about 2 weeks after consent was recorded (at roughly 14 weeks' gestation). This gap created opportunity for loss to follow-up between consent and baseline; thus, the number of mothers completing baseline visit is less than the number of mothers with livebirths. Baseline for infants was birth. Data are n or n (%), unless otherwise specified. *Improved floor defined as concrete, brick, cement, or tile; unimproved floor defined as mud, earth, sand, or dung.

Table 1: **Maternal, household, and infant baseline characteristics of HIV-negative mothers and their liveborn infants**

Results

5280 pregnant women were enrolled from 211 clusters at a median gestational age of 12 (IQR 9–16) weeks (figure). During the antenatal period, 11 women were excluded and one woman was added to the analysis to correct for enrolment errors (figure). 139 (3%) women left the trial or were lost to follow-up, four (<1%) died, 249 (5%) miscarried, and 101 (2%) had stillbirths. 726 (14%) women tested positive for HIV and 114 (2%) had unknown HIV statuses during pregnancy, and were thus excluded from the analysis (outcomes for these infants will be reported separately). Thus 3989 infants were born alive to 3937 HIV-negative women and were included in our analysis. During the postnatal period, 198 (5%) infants died. 3686 (97%) of the remaining 3791 liveborn infants were assessed at the 18-month endpoint (5 [<1%] left the trial and 100 [3%] were lost to follow-up or moved outside Zimbabwe).

At baseline, nearly half of all household members practised open defecation and about a third of households had an improved latrine (table 1). Very few had electricity from the grid but two-thirds had a solar panel (typically for phone charging; table 1). Water access was poor: nearly 40% of households obtained drinking water from unimproved sources, and about 40% had a return-trip walk to their water source of greater than 30 min (data not shown). The mean volume collected was 9·5 L per person per day (table 1). Walking time to water was not associated with volume collected (data not shown). Mothers had completed a median of more than 9 years of schooling, were mostly married, and roughly 10% were infected with *S haematobium* (table 1). Mean infant birthweight was 3·1 kg (SD 0·5), and nearly 90% were born via institutional deliveries (table 1). The frequency of open defecation was higher—and wealth index scores, rates of employment and latrine ownership, and diet quality were lower—in mothers in the standard-of-care group than in those in the other groups. Other maternal and household baseline characteristics and all infant birth characteristics were similar across treatment groups (table 1).

Fidelity of intervention delivery was high (table 2). Among households in the WASH groups, more than 98% received ventilated improved pit latrines and handwashing stations, more than 92% received play mats and play yards, and nearly 80% received 80% or more of the planned deliveries of soap and chlorine solution (table 2). Among households in the IYCF groups, 79% received ≥80% of planned deliveries of the small-quantity lipid-based nutrient supplement (table 2). Among households in the WASH groups and IYCF groups, mothers received a median of 15 (IQR 13–15) of

For peer review only - http://bmjopen.bmj.com/site/about/guidelines.xhtml

	Data source	Standard of care	IYCF	WASH	IYCF plus WASH	Combined WASH	Non-WASH	p value*	Combined IYCF	Non-IYCF	p value*
**Fidelity of intervention delivery**											
Children with 18-month outcomes (on whom inferences are based), n	Trial logs	884	893	918	991	1909	1777		1884	1802	
WASH supplies											
SHINE-installed ventilated improved pit latrine	Trial logs	NA	NA	901/918 (98%)	974/991 (98%)	1875/1909 (98%)	NA	..	NA	NA	..
Two handwashing stations (Tippy Taps) delivered	Trial logs	NA	NA	912/918 (99%)	986/991 (99%)	1898/1909 (99%)	NA	..	NA	NA	..
Baby mat delivered	Trial logs	NA	NA	859/918 (94%)	942/991 (95%)	1801/1909 (94%)	NA	..	NA	NA	..
Play yard delivered	Trial logs	NA	NA	847/918 (92%)	926/991 (93%)	1773/1909 (93%)	NA	..	NA	NA	..
Median liquid soap deliveries (IQR)†	Trial logs	NA	NA	20 (16–20)	20 (18–20)	20 (17–20)	NA	..	NA	NA	..
Received ≥80% of expected soap deliveries	Trial logs	NA	NA	709/918 (77%)	801/991 (81%)	1510/1909 (79%)	NA	..	NA	NA	..
Median Water Guard deliveries (IQR)‡	Trial logs	NA	NA	15 (13–15)	15 (14–15)	15 (14–15)	NA	..	NA	NA	..
Received ≥80% of expected Water Guard deliveries	Trial logs	NA	NA	724/918 (79%)	799/991 (81%)	47232/52656 (80%)	NA	..	NA	NA	..
IYCF supplies											
Median deliveries of small-quantity lipid-based nutrient supplement (IQR)§	Trial logs	NA	13 (12–13)	NA	13 (12–13)	NA	NA	..	13 (12–13)	NA	..
Received ≥11 (80% of expected) deliveries of small-quantity lipid-based nutrient supplement	Trial logs	NA	695/893 (78%)	NA	790/991 (80%)	NA	NA	..	1485/1884 (79%)	NA	..
Behaviour change modules											
Median intervention modules (IQR)‡	Village health worker report	15 (13–15)	15 (13–15)	14 (13–15)	15 (14–15)	15 (13–15)	15 (13–15)	0·321	15 (13–15)	15 (13–15)	0·0009
Completed intervention modules	Village health worker report	14 664/16 523 (89%)	19 673/22 162 (88·8%)	20 636/23 237 (89%)	26 596/29 419 (90%)	47 232/52 656 (90%)	34 337/38 685 (89%)	0·321	46 269/51 581 (90%%)	35 300/39 760 (89%)	0·347
**Participant uptake of promoted behaviours at the 12-month visit**											
Mothers with outcomes at 12 months and 18 months, n	Trial logs	675	702	652	731	1383	1377	..	1433	1327	..
Children with outcomes at 12 months and 18 months, n	Trial logs	682	706	662	741	1403	1388	..	1447	1344	..

(Table 2 continues on next page)

For peer review only - http://bmjopen.bmj.com/site/about/guidelines.xhtml

	Data source	Standard of care	IYCF	WASH	IYCF plus WASH	Combined WASH	Non-WASH	p value*	Combined IYCF	Non-IYCF	p value*
(Continued from previous page)											
WASH behaviours											
Household members who defecate in the open	Maternal report	1088/2407 (45%)	1057/2789 (38%)	27/2917 (1%)	23/3359 (1%)	50/6276 (1%)	50/6276 (1%)	<0·0001	NA	NA	··
Any latrine at household	Observation	214/665 (32%)	291/683 (43%)	642/648 (99%)	725/727 (>99%)	1367/1375 (99%)	505/1348 (37%)	<0·0001	NA	NA	··
Improved latrine at household	Observation	185/664 (28%)	246/683 (36%)	642/648 (99%)	723/727 (>99%)	1365/1375 (99%)	431/1347 (32%)	<0·0001	NA	NA	··
Improved latrine with well trodden path not shared with other households and not used for storage	Observation and maternal report	144/662 (22%)	182/682 (27%)	557/647 (86%)	624/726 (86%)	1181/1373 (86%)	326/1344 (24%)	<0·0001	NA	NA	··
Handwashing station at household	Observation	38/640 (6%)	41/663 (6%)	641/653 (98%)	721/734 (98%)	1362/1387 (98%)	79/1303 (6%)	<0·0001	NA	NA	··
Handwashing station with water and rubbing agent at household	Observation	6 (3%)	13/654 (2%)	513/615 (83%)	560/669 (84%)	1073/1284 (84%)	31/1286 (2%)	<0·0001	NA	NA	··
Ever treats drinking water to make safer	Maternal report	90/667 (13%)	77/684 (11%)	567/650 (87%)	630/727 (87%)	1197/1377 (87%)	167/1351 (12%)	<0·0001	NA	NA	··
Disposes water from cleaning infant nappies with faeces in a latrine	Maternal report	185/660 (28%	239/671 (36%)	478/634 (75%)	558/711 (78%)	1036/1345 (77%)	424/1331 (32%)	<0·0001	NA	NA	··
Play space is visibly clean	Observation	NA	NA	563/615 (92%)	638/688 (93%)	1201/1303 (92%)	NA	NA	NA	NA	··
Child ever observed to eat soil	Maternal report	518/663 (78%)	470/691 (68%)	166/645 (26%)	199/725 (27%)	365/1370 (27%)	988/1354 (73%)	<0·0001	NA	NA	··
Child ever observed to eat chicken faeces	Maternal report	152/663 (23%)	131/689 (19%)	19/646 (3%)	17/724 (2%)	36/1370 (3%)	283/1352 (21%)	<0·0001	NA	NA	··
IYCF behaviours											
Child still breastfeeding	Maternal report	655/677 (97%)	675/695 (97%)	634/653 (97%)	703/734 (96%)	NA	NA	-	1378/1429 (96%)	1289/1330 (97%)	0·494
Mother reports correct ways to feed child during and after illness	Maternal report	424/673 (63%)	485/690 (70%)	393/647 (61%)	499/729 (68%)	NA	NA	-	984/1419 (69%)	817/1320 (62%)	<0·0001
Infant diet met minimum dietary diversity in past 24 h	Maternal report	343/656 (52%)	460/670 (69%)	338/627 (54%)	496/700 (71%)	NA	NA	-	956/1370 (70%)	681/1283 (53%)	<0·0001
Infant consumed iron-rich food in past 24 h	Maternal report	333/664 (50%)	665/692 (96%)	309/643 (48%)	697/729 (96%)	NA	NA	-	1362/1421 (96%)	642/1307 (49%)	<0·0001
Infant consumed animal source food in past 24 h	Maternal report	419/667 (63%)	483/688 (70%)	394/640 (62%)	508/719 (71%)	NA	NA	-	991/1407 (70%)	813/1307 (62%)	<0·0001
Infant consumed vitamin-A-rich food in past 24 h	Maternal report	469/668 (70%)	540/688 (78%)	434/643 (67%)	571/719 (79%)	NA	NA	-	1111/1407 (79%)	903/1311 (69%)	<0·0001
Nutributter consumed in past 24 h	Maternal report	NA	634/668 (95%)	NA	645/714 (90%)	NA	NA	-	1111/1407 (79%)	NA	··

Data are n/N (%), unless otherwise specified. The denominator for indictors of fidelity of intervention delivery are the number of children who provided 18-month outcomes, because inferences of trial outcomes are based on these children. The denominator for indicators of participant uptake of promoted behaviours at the 12-month visit are the number of women (for household-level indicators) and children (for child-level indicators) who provided 12-month and 18-month outcomes. Village health workers were scheduled to visit households monthly to deliver 30 sachets of a small-quantity lipid-based nutrient supplement (sufficient to provide 20 g per day), 1 L of liquid soap, and 150 mL (one bottle) of Water Guard for families of less than five people (two bottles for families of five or more people). The combined WASH group comprised the two WASH-containing groups, whereas the non-WASH group comprised the two groups not including WASH. The combined IYCF group comprised the two IYCF-containing groups, whereas the non-IYCF group comprised the two groups not including IYCF. IYCF=infant and young child feeding. WASH=water, sanitation, and hygiene. SHINE=Sanitation, Hygiene, Infant Nutrition Efficacy trial. NA=not applicable. *p values were adjusted for clustering effect; depending on the variable type, xtgee, multinomial, ordinal regression models with robust variance estimation, and Somers' D for medians, were used for comparing arms while accounting for within-cluster correlation. †Maximum of 20 deliveries. ‡Maximum of 15 deliveries. §Maximum of 13 deliveries.

Table 2: **Intervention delivery and participant uptake by treatment group**

For peer review only - http://bmjopen.bmj.com/site/about/guidelines.xhtml

	Effects by individual treatment group		Main effects combining groups			Unadjusted difference (95% CI)	p value	Adjusted difference* (95% CI)	p value
	n	Mean (SD)	Treatment group	n	Mean (SD)*				
**Length-for-age Z score**									
SOC	878	−1·57 (1·08)	No IYCF	1792	−1·59 (1·08)	Ref	··	Ref	··
IYCF	891	−1·47 (1·06)	IYCF	1879	−1·44 (1·06)	0·16 (0·08 to 0·23)	<0·0001	0·14 (0·07 to 0·21)	<0·0001
WASH	914	−1·61 (1·07)	No WASH	1769	−1·52 (1·07)	Ref	··	Ref	··
IYCF plus WASH	988	−1·41 (1·06)	WASH	1902	−1·50 (1·07)	0·02 (−0·06 to 0·09)	0·698	0·06 (−0·01 to 0·12)	0·119
**Haemoglobin (g/dL)**									
SOC	866	116·5 (11·3)	No IYCF	1759	116·3 (11·8)	Ref	··	Ref	··
IYCF	882	118·4 (11·2)	IYCF	1845	118·3 (11·5)	2·03 (1·28 to 2·79)	<0·0001	1·94 (1·22 to 2·67)	<0·0001
WASH	893	116·1 (12·4)	No WASH	1748	117·5 (11·3)	Ref	··	Ref	··
IYCF plus WASH	963	118·3 (11·8)	WASH	1856	117·2 (12·1)	−0·28 (−1·04 to 0·48)	0·471	−0·60 (−1·37 to 0·17)	0·128
**Weight-for-age Z score**									
SOC	875	−0·72 (1·02)	No IYCF	1785	−0·75 (1·02)	Ref	··	Ref	··
IYCF	888	−0·66 (1·02)	IYCF	1871	−0·62 (0·99)	0·13 (0·07 to 0·20)	<0·0001	0·13 (0·07 to 0·19)	<0·0001
WASH	910	−0·78 (1·02)	No WASH	1763	−0·69 (1·02)	Ref	··	Ref	··
IYCF plus WASH	983	−0·59 (0·97)	WASH	1893	−0·68 (1·00)	0·00 (−0·06 to 0·07)	0·911	0·00 (−0·06 to 0·06)	0·971
**Weight-for-height Z score**									
SOC	875	0·05 (1·07)	No IYCF	1782	0·02 (1·05)	Ref	··	Ref	··
IYCF	888	0·06 (1·11)	IYCF	1870	0·09 (1·07)	0·08 (0·00 to 0·15)	0·036	0·08 (0·02 to 0·15)	0·016
WASH	907	−0·01 (1·04)	No WASH	1763	0·06 (1·09)	Ref	··	Ref	··
IYCF plus WASH	982	0·11 (1·04)	WASH	1889	0·05 (1·04)	−0·01 (−0·08 to 0·07)	0·875	−0·04 (−0·11 to 0·03)	0·257
**Mid-upper-arm circumference Z score**									
SOC	870	0·03 (0·90)	No IYCF	1779	0·01 (0·92)	Ref	··	Ref	··
IYCF	889	0·05 (0·87)	IYCF	1871	0·07 (0·84)	0·07 (0·01 to 0·13)	0·033	0·07 (0·01 to 0·14)	0·018
WASH	909	−0·01 (0·92)	No WASH	1759	0·04 (0·88)	Ref	··	Ref	··
IYCF plus WASH	982	0·09 (0·82)	WASH	1891	0·04 (0·88)	0·00 (−0·06 to 0·06)	0·999	0·01 (−0·05 to 0·07)	0·745
**Head-circumference-for-age Z score**									
SOC	872	−0·26 (1·08)	No IYCF	1778	−0·26 (1·08)	Ref	··	Ref	··
IYCF	885	−0·23 (1·07)	IYCF	1868	−0·19 (1·06)	0·07 (0·00 to 0·14)	0·043	0·06 (0·00 to 0·13)	0·053
WASH	906	−0·27 (1·09)	No WASH	1757	−0·24 (1·07)	Ref	··	Ref	··
IYCF plus WASH	983	−0·16 (1·06)	WASH	1889	−0·21 (1·08)	0·03 (−0·04 to 0·10)	0·372	0·08 (0·01 to 0·15)	0·018

SOC=standard of care. IYCF=infant and young child feeding. Ref=reference. WASH=water, sanitation, and hygiene. *Prespecified baseline variables considered for inclusion in adjusted analyses were maternal age, mid-upper-arm circumference, years of schooling, marital status, employment, religion, maternal capabilities, haemoglobin concentration, household Coping Strategy Index, proportion of household members practising open defecation, faeces observed in yard, household floor type, time to fetch drinking water, chicken ownership, livestock observed inside house, number of household occupants, wealth index quintile, infant low versus normal birthweight, infant sex, and preterm birth; the study factors data collector and calendar year of enrolment were also considered for inclusion.

Table 3: **Effect of WASH and IYCF interventions on infant growth and haemoglobin concentrations at age 18 months (primary and secondary continuous outcomes)**

the 15 intervention visits scheduled between enrolment and 12 months postnatal. Thus, the intervention dose (ie, frequency of contact) was about once per 5 weeks.

Intervention uptake was assessed at 12 months, when 74% of the women were available for the visit (table 2). Women assessed at 12 months were, on average, older, slightly wealthier, more likely to be married and have a diverse diet, and had higher parity than women not assessed at the 12-month visit (appendix). Baseline indicators of sanitation, water, and hygiene were similar (appendix).

At the 12 months post-partum visit, the frequency of open defecation among household members in the WASH groups was 1% compared with 41% in non-WASH groups (table 2). Nearly all households in WASH groups had an improved latrine, and in 86% of households the latrine had a well trodden path and was not being used for storage (compared with 24% in non-WASH groups). 84% of households in the WASH groups had a handwashing station with observed soap or rubbing agent and water compared with 2% of households in non-WASH groups (table 2). 1197 (87%) of 1372 women in the WASH groups reported that they usually treated their drinking water. However, too few samples of water were tested for free chlorine to objectively validate water chlorination. Of the 752 water

For peer review only - http://bmjopen.bmj.com/site/about/guidelines.xhtml

	Effects by individual treatment group		Main effects combining groups			Unadjusted RR (95% CI)	p value	Adjusted RR* (95% CI)	p value
	n	Prevalence (%)	Treatment group	n	Prevalence (%)				
**Stunting (length-for-age Z score less than –2·0)**									
SOC	878	292 (33%)	No IYCF	1792	620 (35%)	Ref	..	Ref	..
IYCF	891	249 (28·0%)	IYCF	1879	514 (27%)	0·79 (0·72–0·87)	<0·0001	0·80 (0·73–0·88)	<0·0001
WASH	914	328 (36%)	No WASH	1769	541 (31%)	Ref	..	Ref	..
IYCF plus WASH	988	265 (27%)	WASH	1902	593 (31%)	1·03 (0·93–1·13)	0·596	0·99 (0·90–1·09)	0·818
**Severe stunting (length-for-age Z score less than –3·0)**									
SOC	878	74 (8%)	No IYCF	1792	160 (9%)	Ref	..	Ref	..
IYCF	891	72 (8%)	IYCF	1879	139 (7%)	0·83 (0·66–1·04)	0·109	0·85 (0·67–1·07)	0·173
WASH	914	86 (9%)	No WASH	1769	146 (8%)	Ref	..	Ref	..
IYCF plus WASH	988	67 (7%)	WASH	1902	153 (8%)	0·99 (0·78–1·24)	0·908	0·96 (0·75–1·23)	0·769
**Anaemia (haemoglobin <105 g/L)**									
SOC	866	117 (14%)	No IYCF	1759	245 (14%)	Ref	..	Ref	..
IYCF	882	81 (9%)	IYCF	1845	193 (10%)	0·75 (0·62–0·90)	0·003	0·76 (0·63–0·92)	0·004
WASH	893	128 (14%)	No WASH	1748	198 (11%)	Ref	..	Ref	..
IYCF plus WASH	963	112 (12%)	WASH	1856	240 (13%)	1·14 (0·95–1·36)	0·151	1·13 (0·93–1·37)	0·235
**Severe anaemia (haemoglobin <70 g/L)**									
SOC	866	3 (<1%)	No IYCF	1759	5 (<1%)	Ref	..	..†	..
IYCF	882	0 (0%)	IYCF	1845	1 (<1%)	0·19 (0·02–1·62)	0·129	..	..
WASH	893	2 (<1%)	No WASH	1748	3 (<1%)	Ref		..	..
IYCF plus WASH	963	1 (<1%)	WASH	1856	3 (<1%)	0·96 (0·20–4·71)	0·959	..	..
**Underweight (weight-for-age Z score less than –2·0)**									
SOC	875	83 (9%)	No IYCF	1785	189 (11%)	Ref	..	Ref	..
IYCF	888	74 (8%)	IYCF	1871	147 (8%)	0·74 (0·60–0·91)	0·005	0·76 (0·62–0·94)	0·010
WASH	910	106 (12%)	No WASH	1765	157 (9%)	Ref	..	Ref	..
IYCF plus WASH	983	73 (7%)	WASH	1893	179 (9%)	1·07 (0·87–1·31)	0·539	1·08 (0·87–1·34)	0·464
**Wasted (weight-for-height Z score less than –2·0)**									
SOC	875	25 (3%)	No IYCF	1782	48 (3%)	Ref	..	Ref	..
IYCF	888	24 (3%)	IYCF	1870	43 (2%)	0·86 (0·57–1·29)	0·459	0·83 (0·55–1·27)	0·381
WASH	907	23 (3%)	No WASH	1763	49 (3%)	Ref	..	Ref	..
IYCF plus WASH	982	19 (2%)	WASH	1889	42 (2%)	0·80 (0·53–1·21)	0·291	0·88 (0·55–1·39)	0·571

RR=relative risk. SOC=standard of care. IYCF=infant and young child feeding. Ref=reference. WASH=water, sanitation, and hygiene. *Prespecified baseline variables considered for inclusion in adjusted analyses were maternal age, mid-upper-arm circumference, years of schooling, marital status, employment, religion, maternal capabilities, haemoglobin concentration, household Coping Strategy Index, proportion of household members practising open defecation, faeces observed in yard, household floor type, time to fetch drinking water, chicken ownership, livestock observed inside house, number of household occupants, wealth index quintile, infant low versus normal birthweight, infant sex, and preterm birth; the study factors data collector and calendar year of enrolment were also considered for inclusion. †Insufficient data to run a regression model.

Table 4: **Effect of WASH and IYCF interventions on infant growth and haemoglobin concentrations at age 18 months (secondary dichotomous outcomes)**

samples from WASH households that were tested at 12 months, only 438 (58%) had more than 0·1 parts per million of free chlorine. Compared with infants in the non-IYCF groups, a higher proportion of those in the IYCF groups had diets that met minimum dietary diversity and had consumed animal-source, iron-rich, and vitamin-A-rich foods in the previous 24 h (table 2). 93% of children in the IYCF groups consumed the small-quantity lipid-based nutrient supplement in the previous 24 h (table 2).

At the 18-month visit, median child age was 18·0 months (IQR 17·8–18·8) and did not differ significantly across treatment groups (data not shown). There was no statistical interaction between the two treatments for any outcome at 18 months, so the main effects of the IYCF and WASH interventions are presented. All follow-up was completed by July 31, 2017. At 18 months, the mean length-for-age Z score was 0·16 (95% CI 0·08–0·23) higher and mean haemoglobin concentration was 2·0 g/L (1·28–2·79) higher in children in the IYCF groups than in those in the non-IYCF groups (table 3). These differences were slightly attenuated in adjusted analyses (table 3). The IYCF intervention reduced stunting by 7·2 percentage points (95% CI 4·3–10·2)—620 (35%) of 1792 children in the non-IYCF groups *vs* 514 (27%) of 1879 children in the IYCF groups. The IYCF intervention also reduced anaemia by 3·5 percentage points (1·3–5·6)—245 (14%) of 1759 children in the non-IYCF groups *vs* 194 (11%) of

For peer review only - http://bmjopen.bmj.com/site/about/guidelines.xhtml

1845 children in the IYCF groups—and significantly increased mean weight-for-age, weight-for-height, and head-circumference-for-age Z scores (tables 3, 4) compared with the non-IYCF interventions.

The WASH intervention had no effect on the mean infant length-for-age Z score or mean haemoglobin concentration compared with the non-WASH interventions (table 3). The WASH interventions had no significant effects on any other growth measurements except for mean head-circumference-for-age Z scores in adjusted analyses (table 3); however, this effect was driven entirely by the IYCF plus WASH group. Coefficient of variation estimates from random-effects models were 0·012 for stunting and 0·12 for anaemia, and 0·16 and 0, respectively, by method-of-moments estimation.

At the 12-month visit, there was a significant statistical interaction between the IYCF and WASH interventions for 7-day prevalence of diarrhoea and acute respiratory infection, so the IYCF, WASH, and IYCF plus WASH groups were each compared with the standard-of-care group (table 5). The 7-day prevalence of diarrhoea was 9% in the standard-of-care group, 13% in the IYCF group, 12% in the WASH group, and 10% in the IYCF plus WASH group (table 5). The prevalence of diarrhoea was 37% (95% CI 4–80) higher in the IYCF group than in the standard-of-care group (p=0·03). No significant differences were noted between the standard-of-care group and the IYCF group, the WASH group, or the IYCF plus WASH group in the prevalence of acute respiratory infection (table 5). There were only eight cases of dysentery overall (one in the standard-of-care group, three in the WASH group, and four in the IYCF plus WASH group).

At 18 months, there was no interaction between the two treatments, so the main effects of the two interventions are presented (table 6). The prevalence of

	n	Prevalence	Difference vs SOC (95% CI)			
			Unadjusted	p value	Adjusted*	p value
**Diarrhoea**						
SOC	678	62 (9%)	Ref	..	Ref	..
IYCF	696	87 (13%)	1·37 (1·04–1·80)	0·027	1·32 (1·00–1·75)	0·054
WASH	666	77 (12%)	1·26 (0·92–1·71)	0·151	1·18 (0·87–1·61)	0·292
IYCF plus WASH	735	76 (10%)	1·13 (0·84–1·53)	0·422	1·05 (0·79–1·40)	0·716
**Acute respiratory infection**						
SOC	676	6 (1%)	Ref	..	Ref	..
IYCF	694	2 (<1%)	0·32 (0·07–1·52)	0·154	0·34 (0·07–1·70)	0·193
WASH	662	8 (1%)	1·36 (0·51–3·63)	0·539	1·75 (0·62–4·90)	0·289
IYCF plus WASH	735	7 (1%)	1·07 (0·35–3·26)	0·899	1·38 (0·42–4·47)	0·595

SOC=standard of care. Ref=reference. IYCF=infant and young child feeding. WASH=water, sanitation, and hygiene. *Prespecified baseline variables considered for inclusion in adjusted analyses were maternal age, mid-upper-arm circumference, years of schooling, marital status, employment, religion, maternal capabilities, haemoglobin concentration, household Coping Strategy Index, proportion of household members practising open defecation, faeces observed in yard, household floor type, time to fetch drinking water, chicken ownership, livestock observed inside house, number of household occupants, wealth index quintile, infant low versus normal birthweight, infant sex, and preterm birth; the study factors data collector and calendar year of enrolment were also considered for inclusion.

Table 5: **Effect of IYCF and WASH interventions on diarrhoea and acute respiratory infection at age 12 months**

	n	Prevalence	Main effects combining groups			Unadjusted difference (95% CI)	p value	Adjusted* difference (95% CI)	p value
			Treatment group	n	Prevalence				
**Diarrhoea**									
SOC	874	83 (9%)	No IYCF	1784	176 (10%)	Ref	..	Ref	..
IYCF	883	65 (7%)	IYCF	1866	175 (9%)	0·95 (0·77–1·16)	0·585	0·97 (0·79–1·19)	0·750
WASH	910	93 (10%)	No WASH	1757	148 (8%)	Ref	..	Ref	..
IYCF plus WASH	983	110 (11%)	WASH	1893	203 (11%)	1·28 (1·04–1·57)	0·020	1·15 (0·93–1·41)	0·191
**Acute respiratory infection**									
SOC	875	6 (1%)	No IYCF	1785	11 (1%)	Ref	..	Ref	..
IYCF	879	4 (<1%)	IYCF	1856	9 (<1%)	0·77 (0·28–2·13)	0·617	0·76 (0·28–2·03)	0·582
WASH	910	5 (1%)	No WASH	1754	10 (1%)	Ref	..	Ref	..
IYCF plus WASH	977	5 (1%)	WASH	1887	10 (1%)	0·96 (0·36–2·55)	0·930	1·29 (0·48–3·43)	0·611
**Death**									
SOC	959	50 (5%)	No IYCF	1954	99 (5%)	Ref	..	Ref	..
IYCF	958	40 (4%)	IYCF	2020	92 (5%)	0·88 (0·66–1·18)	0·406	0·87 (0·65–1·18)	0·376
WASH	995	49 (5%)	No WASH	1917	90 (5%)	Ref	..	Ref	..
IYCF plus WASH	1062	52 (5%)	WASH	2057	101 (5%)	1·04 (0·78–1·39)	0·790	0·96 (0·72–1·30)	0·808

SOC=standard of care. IYCF=infant and young child feeding. Ref=reference. WASH=water, sanitation, and hygiene. *Prespecified baseline variables considered for inclusion in adjusted analyses were maternal age, mid-upper-arm circumference, years of schooling, marital status, employment, religion, maternal capabilities, haemoglobin concentration, household Coping Strategy Index, proportion of household members practising open defecation, faeces observed in yard, household floor type, time to fetch drinking water, chicken ownership, livestock observed inside house, number of household occupants, wealth index quintile, infant low versus normal birthweight, infant sex, and preterm birth; the study factors data collector and calendar year of enrolment were also considered for inclusion.

Table 6: **Effect of IYCF and WASH interventions on diarrhoea, acute respiratory infection, and mortality at age 18 months**

For peer review only - http://bmjopen.bmj.com/site/about/guidelines.xhtml

diarrhoea did not differ between the IYCF groups and non-IYCF groups, but was 28% higher in the WASH groups than in the non-WASH groups—a difference was significant in unadjusted but not adjusted analyses (table 6). Neither IYCF nor WASH significantly affected the prevalence of acute respiratory infection (table 6), and only ten cases of dysentery (two in the standard-of-care group, three in the IYCF group, two in the WASH group, and three in the IYCF plus WASH group) were recorded.

Cumulative mortality at 18 months was 5·2% in the standard-of-care group, 4·2% in the IYCF group, 4·9% in the WASH group, and 4·9% in the IYCF plus WASH group, and did not differ significantly between groups. Treatment group effects on length-for-age Z scores, haemoglobin concentrations, stunting, anaemia, and diarrhoea prevalence were similar in all prespecified sensitivity analyses compared with those in the total analytic sample (data not shown). However, in the prespecified subgroup analysis by infant sex, boys had poorer linear growth than girls at 18 months, with lower mean length-for-age Z scores (–1·66 [95% CI –1·72 to –1·61] *vs* –1·35 [–1·40 to –1·30]) and a higher proportion of stunting (36·7% [34·4 to 39·0] *vs* 25·0% [23·1 to 27·2]). Child sex modified the effects of the IYCF intervention on length-for-age Z scores ($p_{interaction}$=0·016) but not stunting ($p_{interaction}$=0·420). The IYCF intervention was more efficacious in increasing mean length-for-age Z scores among boys (0·24 [95% CI 0·14 to 0·34]) than among girls (0·07 [95% CI –0·04 to 0·17]). Child sex did not modify the effect of the IYCF intervention on haemoglobin concentration or anaemia, or the effect of the WASH intervention on either primary outcome (data not shown).

Among 653 serious adverse events, none were judged to be related to the trial interventions (appendix). Three adverse events were judged to be related to trial interventions (one possibly related, one probably related, and one definitely related). In the IYCF group, a child with congenital abnormalities complained of abdominal discomfort after ingestion of the small-quantity lipid-based nutrient supplement (possibly related). In the IYCF plus WASH group, one child had diarrhoea after consumption of the small-quantity lipid-based nutrient supplement (probably related). In the WASH group, one child was fed WaterGuard by a sibling (definitely related). The child was reviewed at the clinic and treated with paracetamol for 3 days. All three cases resolved completely with no sequelae.

Discussion

We tested the independent and combined effects of an infant feeding intervention and a household WASH intervention on attained child length and haemoglobin concentration at 18 months of age. Interventions were delivered with high fidelity of intervention and substantial contrast was achieved in WASH and IYCF hardware, commodities, and behaviours. Consistent with decades of complementary feeding research,[3,22] the IYCF interventions increased linear growth and haemoglobin concentrations, reduced stunting by 21%, reduced anaemia by 24%, and increased head circumference and ponderal growth compared with the non-IYCF interventions. Although we could not separate out the effects of complementary feeding education from those of the lipid-based nutrient supplement, our formative work showed that both components are important.[12,13] By contrast, we detected no benefit for the WASH intervention on any reported child health outcomes. Length-for-age Z scores, haemoglobin concentrations, proportions of stunted and anaemic children, and indicators of ponderal growth were similar in WASH and non-WASH groups. Head circumference at the 18-month visit was greater in the WASH than in the non-WASH groups in adjusted analyses, but this difference was driven by the IYCF plus WASH group, and was thus unlikely to be a true WASH effect.

The IYCF intervention did not decrease the 7-day prevalence of diarrhoea at either the 12-month or 18-month visits. We think that the increased diarrhoea prevalence in the IYCF group at 12 months was a chance occurrence. Although diarrhoea risk is moderately increased by oral iron when given as a medicinal supplement, this risk is nearly absent when iron is given as a food fortificant.[23]

The WASH intervention had no effect on diarrhoea prevalence at either the 12-month or 18-month visits. This finding is not consistent with those of 2015 Cochrane reviews on water chlorination[24] and handwashing promotion,[25] in which these interventions were estimated to reduce diarrhoea by about 25%. Most of the studies included in these reviews (and nearly all the studies which showed a significant effect on diarrhoea) had very high intervention doses—ie, daily-to-weekly contact between behaviour-change promoters and study participants—which was greater than the monthly contact delivered in SHINE. Although one water chlorination trial[26] published since the 2015 Cochrane reviews showed a 36% reduction in diarrhoea with monthly behaviour change intervention contacts, at the midpoint between intervention visits staff visited to measure chlorine residuals—ie, participants received a visit about water chlorination every 2 weeks. Furthermore, several studies included in the Cochrane reviews showed no effect on diarrhoea, even with daily-to-weekly intervention doses. Finally, follow-up studies[27,28] suggest that the effect of these interventions on diarrhoea is not sustained once frequent intervention contacts end. Thus, adherence to handwashing and water-chlorination interventions (both highly dependent on sustained behaviour change) might not be sufficient to reduce diarrhoea when intervention dose is less frequent than monthly, even when behaviour-change messages are based on extensive formative research, delivered by highly trained workers, and accompanied by free

For peer review only - http://bmjopen.bmj.com/site/about/guidelines.xhtml

provision of soap and chlorine, as in SHINE. Intervention dose is often not reported in WASH behaviour-change studies and, to our knowledge, has not been extracted in any systematic review of WASH studies,[24,25,29,30] suggesting that importance of very frequent sustained behaviour-change promotion for home-based water chlorination and handwashing promotion might not be widely recognised.

Several other aspects of the trial could be important. First, we intervened at the household rather than community level, because we reasoned that young children spend most of their time within their own household. Increased community sanitation coverage, even in sparsely populated areas, might be required to affect growth. Although open defecation was reduced in the WASH households from around 50% to less than 1%, we estimate that community-level open defecation in the WASH clusters was reduced from around 55% to around 40% (data not shown). Decreased open defecation[31] and higher sanitation coverage[32] at the community level have been associated with reduced stunting. A trial[33] in Mali showed that communities randomised to a community-led sanitation-intervention had improved linear growth compared with control communities. Finally, although the SHINE WASH intervention considerably reduced geophagia and consumption of chicken faeces by maternal history, it did not prevent these behaviours (27% of WASH mothers still reported they had observed geophagia at the 12-month visit). Analyses of structured observation and in-depth interview data are underway, and will provide detailed information about how the play space was used and whether and by what magnitude this intervention interrupted faecal–oral microbial transmission due to child exploratory play.

SHINE is the third trial in which a WASH intervention alone or in combination with an IYCF intervention had no effect on linear growth.[34,35] Although these findings do not unequivocally prove that an integrated WASH–nutrition approach will never improve linear growth in any context, these trials included more than 18 000 children in three diverse settings where stunting is prevalent and environmental hygiene and infant diets are poor. We have three potential explanations for the lack of effects of WASH interventions on linear growth. First, our hypothesis could have been incorrect—perhaps reduction of faecal ingestion does not reduce environmental enteric dysfunction, or perhaps prevention of environmental enteric dysfunction does not improve linear growth. The cause and growth effects of environmental enteric dysfunction remain poorly understood. Laboratory analyses of biomarkers of environmental enteric dysfunction from the SHINE trial are underway.

Second, our hypothesis could have been correct, but the WASH interventions used were not effective enough to facilitate linear growth or, in two of the three trials, to reduce diarrhoea. Throughout history, linear growth and child health have improved after substantial socio-economic development, as occurred after the industrial revolution in Europe and more recently in Latin America. These health benefits have been partly attributed to provision of piped water into homes, sewage systems, and flush toilets. For example, in Brazil, where stunting declined from 37% to 7% between 1996 and 2007, expansion of water supply and sanitation services, particularly for the poorest people (ie, in the lowest wealth quintile), was one of four factors credited for approximately two-thirds of the stunting decline.[36] The absence of effects on linear growth and diarrhoea in SHINE suggests that the household-level interventions we implemented (point-of-use water chlorination, handwashing stations not connected to a water source, and improved pit latrines) might have little effect on child health, even on diarrhoea, unless the behaviour-change intervention is sustained daily or weekly, as implemented by the WASH Benefits Bangladesh trial and other efficacy trials.[34] Moreover, the WASH Benefits Bangladesh trial suggested that even with this level of sustained intervention dose, these interventions might not be efficacious enough to improve growth. One important intervention untested in any of the three trials is provision of an on-plot, sustained, high-quality water supply—the aspirational goal of the Sustainable Development Goals (although most of the households in Bangladesh were less than 5 minutes' walk from an improved water source). In summary, to achieve and sustain diarrhoea prevention at scale and improve linear growth might require new, innovative interventions that are less dependent on behaviour change and more efficacious in reducing faecal exposure—a paradigm shift away from how rural WASH programmes are delivered.

Third, the trials did not address intergenerational prenatal factors. Already at 1 month of age, mean length-for-age Z score for infants in the SHINE trial was −0·85 (SD 1·25) and 16% were stunted (data not shown) despite high rates of early initiation and exclusive breastfeeding[16] and installation of latrines and tippy taps during pregnancy. Preconception dietary supplementation of mothers (NCT01883193) and planned studies by our group to characterise the drivers of poor fetal growth during pregnancy could inform future preconception or prenatal interventions.

There is a large movement to scale up integrated WASH–nutrition interventions for stunting prevention.[37] The SHINE trial provides high-level evidence that elementary WASH interventions delivered at the household level in rural areas of low-income and middle-income countries are unlikely to reduce stunting and might not reduce diarrhoea, and that implementation of these WASH interventions together with IYCF interventions will not reduce stunting more than implementation of IYCF alone. Our findings provide an urgent call

For peer review only - http://bmjopen.bmj.com/site/about/guidelines.xhtml

for greater investment in the WASH sector to identify and deliver more efficacious interventions.

Contributors

JHH and GM were the co-primary investigators of the trial. MNNM led the development of the interventions and managed implementation. RN developed and managed all information technology, data management, and data analysis. LHM was the senior statistician. RJS coauthored the original protocols and contributed to design and implementation of the trial and data analysis and interpretation. NVT managed field operations, KM managed the laboratory, FM supervised the data collection nurses, and BM supervised the field data supervisors. CMC and AC contributed to trial design and served as liaisons to the departments of nursing and nutrition, respectively, in the Ministry of Health and Child Care. BC contributed to data management and data analysis. LES contributed to analysis and interpretation of SHINE. JMT, ADJ, ARM, and JAM contributed to design and interpretation of SHINE. AJP managed data collection and laboratory teams, and directed all clinical and laboratory aspects of the trial. All authors contributed to, reviewed, and approved this Article.

Declaration of interests

We declare no competing interests.

Acknowledgments

The SHINE trial is funded by the Bill & Melinda Gates Foundation (OPP1021542 to Johns Hopkins Bloomberg School of Public Health and OPP1143707 to Zvitambo Institute for Maternal and Child Health Research), the UK Department for International Development, the Wellcome Trust (093768/Z/10/Z and 108065/Z/15/Z), the Swiss Agency for Development and Cooperation (8106727), UNICEF (PCA-2017-0002), and the US National Institutes of Health (R01 HD060338/HD/NICHD). We thank all the mothers, babies, and their families who participated in the trial, the leadership and staff of the Ministry of Health and Child Care in Chirumanzu and Shurugwi districts and Midlands Province (especially environmental health, nursing, and nutrition) for their roles in operationalisation of the study procedures, the Ministry of Local Government officials in each district who supported and facilitated field operations, Phillipa Rambanepasi and her team for proficient management of all the finances, Virginia Sauramba for management of compliance issues, and the programme officers at the Gates Foundation and the Department for International Development, who enthusiastically worked with us over a long period to make SHINE happen.

References

1 Prendergast AJ, Humphrey JH. The stunting syndrome in developing countries. *Paediatr Int Child Health* 2014; **34:** 250–65.
2 SHINE Trial team. The Sanitation Hygiene Infant Nutrition Efficacy (SHINE) trial: rationale, design, and methods. *Clin Infect Dis* 2015; **61** (suppl 7): S685–702.
3 Panjwani A, Heidkamp R. Complementary feeding interventions have a small but significant impact on linear and ponderal growth of children in low-and middle-income countries: a systematic review and meta-analysis. *J Nutr* 2017; **147:** 2169S–78S.
4 Richard SA, Black RE, Gilman RH, et al. Diarrhea in early childhood: short-term association with weight and long-term association with length. *Am J Epidemiol* 2013; **178:** 1129–38.
5 UNICEF. Strategy for improved nutrition of children and women in developing countries. New York: United Nations Children's Fund, 1990.
6 Rosenberg IH, Solomons NW. The potential for antidiarrheal and nutrient-sparing effects of oral antibiotic use in children: a position paper. *AJCN* 1978; **31:** 2202–07.
7 Humphrey JH. Child undernutrition, tropical enteropathy, toilets, and handwashing. *Lancet* 2009; **374:** 1032–35.
8 UNAIDS, Zimbabwe Ministry of Health and Child Care. Global AIDS response progress report 2016. http://www.unaids.org/sites/default/files/country/documents/ZWE_narrative_report_2016.pdf (accessed Nov 24, 2018).
9 Midzi N, Mduluza T, Chimbari MJ, et al. Distribution of schistosomiasis and soil transmitted helminthiasis in Zimbabwe: towards a national plan of action for control and elimination. *PLoS Negl Trop Dis* 2014; **8:** e3014.
10 Ntozini R, Marks S, Mangwandu G, et al. Using geographic information systems and spatial analysis methods to assess household water access and sanitation coverage in the SHINE trial. *Clin Infect Dis* 2015; **61:** S716–25.
11 Moulton LH. Covariate-based constrained randomization of group-randomized trials. *Clin Trials* 2004; **1:** 297–305.
12 Paul KH, Muti M, Chasekwa B, et al. Complementary feeding messages that target cultural barriers enhance both the use of lipid-based nutrient supplements and underlying feeding practices to improve infant diets in rural Zimbabwe. *Matern Child Nutr* 2012; **8:** 225–38.
13 Desai A, Smith L, Mbuya M, et al. The SHINE trial infant feeding intervention: pilot study of effects on maternal learning and infant diet quality in rural Zimbabwe. *Clin Infect Dis* 2015; **61:** S710–15.
14 Ngure FM, Humphrey JH, Mbuya MN, et al. Formative research on hygiene behaviors and geophagy among infants and young children and implications of exposure to fecal bacteria. *Am J Trop Med Hyg* 2013; **89:** 709–16.
15 Mbuya MNN, Tavengwa NV, Stoltzfus RJ, et al. Design of an intervention to minimize ingestion of fecal microbes by young children in rural Zimbabwe. *Clin Infect Dis* 2015; **61** (suppl 7): S703–09.
16 Mbuya M, Matare CR, Tavengwa NV, et al. Early initiation and exclusivity of breastfeeding in rural Zimbabwe: impact of a breastfeeding intervention delivered by village health workers. *Curr Dev Nutr* (in press).
17 Arimond M, Zeilani M, Jungjohann S, et al. Considerations in developing lipid-based nutrient supplements for prevention of undernutrition: experience from the International Lipid-Based Nutrient Supplements (iLiNS) Project. *Matern Child Nutr* 2015; **11** (suppl 4): 31–61.
18 Matare C, Mbuya M, Pelto G, Dickin K, Maluccio J, Stoltzfus R. Assessing maternal capabilities in the SHINE trial: a heretofore overlooked piece in the causal pathways to child health. *Clin Infect Dis* 2015; **61** (suppl 7): S745–51.
19 Chasekwa B, Maluccio JA, Ntozini R, et al. Measuring wealth in rural communities: lessons from the Sanitation, Hygiene, Infant Nutrition Efficacy (SHINE) trial. *PLoS One* 2018; **13:** e0199393.
20 FAO, FHI 360. Minimum dietary diversity for women: a guide for measurement. Rome: FAO, 2016.
21 Maxwell D, Watkins B, Wheeler R, Collins G. The Coping Strategy Index: a tool for rapid measurement of household food security and the impact of food aid programs in humanitarian emergencies. Nairobi: CARE and WFP, 2003.
22 Dewey KG, du-Afarwuah S. Systematic review of the efficacy and effectiveness of complementary feeding interventions in developing countries. *Matern Child Nutr* 2008; **4** (suppl 1): 24–85.
23 Richard SA, Zavaleta N, Caulfield LE, Black RE, Witzig RS, Shankar AH. Zinc and iron supplementation and malaria, diarrhea, and respiratory infections in children in the Peruvian Amazon. *Am J Trop Med Hyg* 2006; **75:** 126–32.
24 Clasen TF, Alexander KT, Sinclair D, et al. Interventions to improve water quality for preventing diarrhoea. *Cochrane Database Syst Rev* 2015; **10:** CD004794.
25 Ejemot-Nwadiaro RI, Ehiri JE, Arikpo D, Meremikwu MM, Critchley JA. Hand washing promotion for preventing diarrhoea. *Cochrane Database Syst Rev* 2015; **9:** CD004265.
26 Ercumen A, Naser AM, Unicomb L, Arnold BF, Colford JM Jr, Luby SP. Effects of source-versus household contamination of tubewell water on child diarrhea in rural Bangladesh: a randomized controlled trial. *PLoS One* 2015; **10:** e0121907.
27 Luby SP, Mendoza C, Keswick BH, Chiller TM, Hoekstra RM. Difficulties in bringing point-of-use water treatment to scale in rural Guatemala. *Am J Trop Med Hyg* 2008; **78:** 382–87.
28 Luby SP, Agboatwalla M, Bowen A, Kenah E, Sharker Y, Hoekstra RM. Difficulties in mantaining improved handwashing behavior, Karachi, Pakistan. *Am J Trop Med Hyg* 2009; **81:** 140–45.
29 Prüss-Ustün A, Bartram J, Clasen T, et al. Burden of disease from inadequate water, sanitation and hygiene in low- and middle-income settings: a retrospective analysis of data from 145 countries. *Trop Med Int Health* 2014; **19:** 894–905.
30 Wolf J, Prüss-Ustün A, Cumming O, et al. Assessing the impact of drinking water and sanitation on diarrhoeal disease in low- and middle-income settings: systematic review and meta-regression. *Trop Med Int Health* 2014; **19:** 928–42.

For peer review only - http://bmjopen.bmj.com/site/about/guidelines.xhtml

31 Spears D, Ghosh A, Cumming O. Open defecation and childhood stunting in India: an ecological analysis of new data from 112 districts. *PloS One* 2013; **8:** e73784.

32 Harris M, Alzua ML, Osbert N, Pickering A. Community-level sanitation coverage more strongly associated with child growth and household drinking water quality than access to a private toilet in rural Mali. *Environ Sci Technol* 2017; **51:** 7219–27.

33 Pickering AJ, Djebbari H, Lopez C, Coulibaly M, Alzua ML. Effect of a community-led sanitation intervention on child diarrhoea and child growth in rural Mali: a cluster-randomised controlled trial. *Lancet Glob Health* 2015; **3:** e701–11.

34 Luby SP, Rahman M, Arnold BF, et al. Effects of water quality, sanitation, handwashing, and nutritional interventions on diarrhoea and child growth in rural Bangladesh: a cluster randomised controlled trial. *Lancet Glob Health* 2018; **6:** e302–15.

35 Null C, Stewart CP, Pickering AJ, et al. Effects of water quality, sanitation, handwashing, and nutritional interventions on diarrhoea and child growth in rural Kenya: a cluster-randomised controlled trial. *Lancet Glob Health* 2018; **6:** e316–29.

36 Monteiro CA, Benicio MHDB, Konno SC, Silva ACFd, Lima ALLd, Conde WL. Causes for the decline in child under-nutrition in Brazil, 1996–2007. *Revista de Saude Publica* 2009; **43:** 35–43.

37 WHO, UNICEF. Improving nutrition outcomes with better water, sanitation and hygiene: practical solutions for policies and programmes. Geneva: World Health Organization, 2015.

For peer review only

BMJ Open

Child Health, Agriculture and Integrated Nutrition (CHAIN): protocol for a randomized controlled trial of improved infant and young child feeding in rural Zimbabwe

Journal:	*BMJ Open*
Manuscript ID	bmjopen-2021-056435.R1
Article Type:	Protocol
Date Submitted by the Author:	10-Jun-2022
Complete List of Authors:	Smith, Laura ; Cornell University,<br>Chagwena, Dexter; Zvitambo Institute for Maternal and Child Health Research, Nutrition ; Ministry of Health and Child Care, Nutrition<br>Bourke, Claire ; Blizard Institute, Queen Mary University of London, UK<br>Robertson, Ruairi; Blizard Institute, Queen Mary University of London, UK,<br>Fernando, Shamiso; Zvitambo Institute for Maternal and Child Health Research, Nutrition<br>Tavengwa, Naume; Zvitambo Institute for Maternal and Child Health Research, Nutrition<br>Cairns, Jill; CIMMYT<br>Ndhlela, Thokozile; CIMMYT<br>Matumbu, Exhibit; Zvitambo Institute for Maternal and Child Health Research, Nutrition<br>Brown, Tim; Queen Mary University of London<br>Datta, Kavita; Queen Mary University of London<br>Mutasa, Batsirai; Zvitambo Institute for Maternal and Child Health Research, Nutrition<br>Tengende, Alice; Zvitambo Institute for Maternal and Child Health Research, Nutrition<br>Chidhanguro, Dzivaidzo; Zvitambo Institute for Maternal and Child Health Research, Nutrition<br>Langhaug, Lisa; Zvitambo Institute for Maternal and Child Health Research, Nutrition<br>Makanza, Maggie; Zvitambo Institute for Maternal and Child Health Research, Nutrition<br>Chasekwa, Bernard; Zvitambo Institute for Maternal and Child Health Research, Nutrition<br>Mutasa, Kuda; Zvitambo Institute for Maternal and Child Health Research, Harare, Zimbabwe<br>Swann, Jonathan; University of Southampton Faculty of Medicine<br>Kelly, Paul; Barts and The London School of Medicine<br>Ntozini, Robert; Zvitambo Institute, Biostatistics & IT<br>Prendergast, Andrew ; Queen Mary University of London,
**Primary Subject Heading**:	Nutrition and metabolism
Secondary Subject Heading:	Global health, Epidemiology, Paediatrics, Immunology (including allergy)

Keywords:	Nutrition < TROPICAL MEDICINE, Immunology < THORACIC MEDICINE, QUALITATIVE RESEARCH, Community child health < PAEDIATRICS, Nutritional support < GASTROENTEROLOGY

SCHOLARONE™
Manuscripts

1.1.1 Child Health, Agriculture and Integrated Nutrition (CHAIN): protocol for a randomized controlled trial of improved infant and young child feeding in rural Zimbabwe

Laura E Smith[1,2], Dexter T. Chagwena[1, 3], Claire D. Bourke[4], Ruairi C. Robertson[4], Shamiso Fernando[1], Naume V. Tavengwa[1], Jill Cairns[5], Thokozile Ndhlela[5], Exhibit Matumbu[1], Tim Brown[7], Kavita Datta[7], Batsirai Mutasa[1], Alice Tengende[1], Dzivaidzo Chidhanguro[1], Lisa Langhaug[1], Maggie Makanza[1], Bernard Chasekwa[1], Kuda Mutasa[1], Jonathan Swann[6], Paul Kelly[4], Robert Ntozini[1], Andrew J. Prendergast[1,4]

[1]Zvitambo Institute for Maternal and Child Health Research, Harare, Zimbabwe

[2]Department of Public and Ecosystem Health, Cornell University, Ithaca, NY, USA

[3]Ministry of Health and Child Care, Harare, Zimbabwe

[4]Blizard Institute, Queen Mary University of London, UK

[5]CIMMYT, Harare, Zimbabwe

[6]University of Southampton, UK

[7]School of Geography, Queen Mary University of London, UK

Short title: CHAIN Trial Protocol

Word count: 5835

Abstract: 395

Corresponding author

Dexter Chagwena, Zvitambo Institute for Maternal and Child Health Research, 16 Lauchlan Ave, Meyrick Park, Mabelreign, Harare.

Tel +263 242 306 028. Email tungadex@gmail.com or d.chagwena@zvitambo.com

For peer review only - http://bmjopen.bmj.com/site/about/guidelines.xhtml

For peer review only

ABSTRACT

Introduction

Over one-quarter of children in sub-Saharan Africa are stunted; however, commercial supplements only partially meet child nutrient requirements, cannot be sustainably produced, and do not resolve physiological barriers to adequate nutrition (e.g. inflammation, microbiome dysbiosis and metabolic dysfunction). Redesigning current infant and young child feeding (IYCF) interventions using locally available foods to improve intake, uptake and utilization of nutrients could ameliorate underlying pathogenic pathways and improve infant growth during the critical period of complementary feeding, to reduce the global burden of stunting.

Methods and Analysis

Child Health Agriculture Integrated Nutrition (CHAIN) is an open-label, individual household randomized trial comparing the effects of IYCF versus "IYCF-plus" on nutrient intake during infancy. The IYCF intervention comprises behaviour-change modules to promote infant nutrition delivered by community health workers, plus small-quantity lipid-based nutrient supplements (SQ-LNS) from 6-12 months of age which previously reduced stunting at 18 months of age by ~20% in rural Zimbabwe. The "IYCF-plus" intervention provides these components plus powdered NUA-45 bio-fortified sugar beans, whole egg powder, moringa leaf powder and pro-vitamin A maize. The trial will enrol 192 infants between 5-6 months of age in Shurugwi district, Zimbabwe. Research nurses will collect data plus blood, urine and stool samples at baseline (5-6 months of age) and endline (9-11 months of age). The primary outcome is energy intake, measured by multi-pass 24-hour dietary recall at 9-11 months of age. Secondary outcomes include nutrient intake, anthropometry and haemoglobin concentration. Nested laboratory sub-studies will evaluate the gut microbiome,

For peer review only - http://bmjopen.bmj.com/site/about/guidelines.xhtml

environmental enteric dysfunction, metabolic phenotypes and innate immune function. Qualitative sub-studies will explore the acceptability and feasibility of the IYCF-plus intervention among participants and community stakeholders, and the effects of migration on food production and consumption.

Ethics and Dissemination

This trial is registered at clinicaltrials.gov (NCT04874688) and was approved by the Medical Research Council of Zimbabwe (MRCZ/A/2679) with the final version 1.4 approved on August 20, 2021. Dissemination of trial results will be conducted through the Community Engagement Advisory Board in the study district and through national-level platforms.

Strengths and Limitations of this study:

- Efficient trial design building upon previous results from rural Zimbabwe.
- Community-based study utilising foods that could provide a sustainable solution to child growth in rural Africa.
- Improved infant and young child feeding may close nutrient gaps, ameliorate underlying pathogenic pathways and improve infant growth during the critical period of complementary feeding.
- Measurement of a broad range of biomarkers.
- Limitation of a short follow-up period to measure outcomes.

Trial registration: NCT04874688. Registered 6 May 2021- Retrospectively registered, https://clinicaltrials.gov/ct2/show/NCT04874688

For peer review only - http://bmjopen.bmj.com/site/about/guidelines.xhtml

INTRODUCTION

Undernutrition underlies 45% of child deaths among children <5 years[1]. Linear growth failure in childhood is the most prevalent form of undernutrition globally. An estimated 149 million children under 5 years of age are stunted, with a length-for-age Z-score (LAZ) more than two standard deviations below the population median[2]. Stunting affects almost one-third of children in sub-Saharan Africa, leading to reduced human capacity and increased long-term risk of chronic disease; it is therefore a surrogate marker of child health inequalities[1].

The period from 6-24 months of age is one of the most critical phases of linear growth[3], when stunting prevalence peaks due to high demand for nutrients coupled with limited quality and quantity of complementary foods[2]. Infant diets in rural sub-Saharan Africa often have low dietary diversity and a heavy reliance on white maize, which is high in starch and low in other nutrients. Interventions to improve infant and young child feeding (IYCF) typically include nutrition counselling to caregivers, plus a combination of commercial and locally available food products with or without micronutrients. However, a meta-analysis[4] of 42 studies showed only a modest impact of complementary feeding interventions on linear growth. Small-quantity lipid-based nutrient supplements (SQ-LNS), which are micronutrient-fortified ready-to-use products, show a small but measurable impact on LAZ.

We recently conducted the Sanitation Hygiene Infant Nutrition Efficacy (SHINE) trial[5], a 2×2 factorial cluster-randomized trial of improved water, sanitation and hygiene (WASH) and improved IYCF in rural Zimbabwe. A combination of IYCF messages and provision of SQ-LNS between 6-18 months of age improved LAZ of children at age 18 months by +0.16 (95%CI 0.08, 0.23) and reduced stunting by 20%[5]. The intervention also increased haemoglobin by 0.20 g/dL (95%CI 0.13, 0.28), and reduced anaemia by almost 25%[5].

For peer review only - http://bmjopen.bmj.com/site/about/guidelines.xhtml

However, despite this intensive IYCF intervention, 32%, 73%, and 23% of infants did not meet energy, folate and zinc/iron dietary intake requirements, respectively, as determined by 24-hour recall in a subgroup at 12 months, and over one-quarter remained stunted. We also found evidence for barriers to infant nutrition, including caregiver capabilities[6], household characteristics[7], infant enteropathogen carriage[8], and systemic and intestinal inflammation[9,10], which were not resolved by the IYCF intervention[11]. Thus, we believe that persistent barriers to nutrient intake, uptake and utilization limited the impact of the IYCF intervention.

Nutrient intake is influenced by food insecurity, household purchasing power, and women's disempowerment to make decisions about land use, crop choice, and distribution of food within the household; and inequitable gender beliefs[7,12]. Nutrient uptake and utilization are influenced by intestinal pathologies which are highly prevalent among children in low-resource settings[3]. First, environmental enteric dysfunction (EED), a subclinical pathology of the small intestine characterized by intestinal inflammation and blunted villi, may impair efficient intestinal uptake of nutrients. Second, disturbance of the normal assembly of the gut microbiota may impair its roles in immune maturation, intestinal development, and nutrient metabolism, thereby impairing growth[13]. Third, systemic inflammation arising from gut pathology increases energy requirements, reduces circulating micronutrients, and inhibits the growth hormone axis[14]. Previously, barriers to intake, uptake and utilization of nutrients have largely been addressed in isolation; however, addressing these in parallel could ultimately improve growth and development in young children. Here, we present methodology for the Child Health Agriculture Integrated Nutrition (CHAIN) trial which aims to address each of these barriers together through a randomized IYCF intervention.

For peer review only - http://bmjopen.bmj.com/site/about/guidelines.xhtml

STUDY OVERVIEW

CHAIN is an open-label, individually randomized household trial comparing the effects of IYCF versus an enhanced IYCF intervention ("IYCF-plus") on energy and nutrient intake, growth, and haemoglobin in infants at high risk of stunting. The trial was completed in February 2022 while the trial design paper was under review. The overarching goal of this trial is to fill key nutrient gaps among infants in rural sub-Saharan Africa through an improved IYCF intervention using locally available foods that could ultimately be sustainable through agriculture. 192 rural Zimbabwean children were enrolled in the trial between 5-6 months of age; interventions were delivered from 6-12 months of age and the primary endline outcome of energy intake was assessed at 9 months of age (window 9-11 months). Interventions continued to be delivered until 12 months of age regardless of whether the endline visit was already completed, and all children were followed for endline visits to obtain the primary outcome. Our approach builds on the SHINE IYCF package, which reduced stunting but did not close all nutrient gaps[5]. We expect the CHAIN population will be similar to the SHINE population described above, as they are from the same rural community. CHAIN will test the impact of additional foods (powdered NUA-45 bio-fortified sugar beans, whole egg powder, moringa leaf powder and pro-vitamin A maize) that are nutrient-rich, culturally acceptable, locally sustainable and may have functional properties to ameliorate underlying pathogenic pathways, thereby tackling the identified barriers to nutrient intake, uptake and utilization. For the duration of the trial, these foods were provided by community health workers as dried powders, which can be added to infant porridge as point-of-use fortificants. However, if shown to be efficacious, this trial would provide strong proof-of-principle that a comprehensive improvement to complementary feeding using locally available foods can substantially improve child nutrition. The chosen intervention foods have potential for local communities ultimately to become self-sufficient through

For peer review only - http://bmjopen.bmj.com/site/about/guidelines.xhtml

modifications and adaptations to local agricultural systems that include local production and processing of these foods.

STUDY OBJECTIVES

Objective 1. Evaluate the effect of an enhanced infant feeding intervention ("IYCF-plus") on energy intake at 9 months of age (window 9-11 months) in a randomized, community-based trial in rural Zimbabwe. *We hypothesize that provision of powdered fortificants (pro-vitamin A maize, NUA45 sugar beans, moringa and egg) for infants from 6 months of age will provide more energy at 9 months of age than the current standard-of-care IYCF intervention (trial primary outcome).*

Objective 2. Evaluate the impact of IYCF-plus on nutrient intake, growth, and haemoglobin at 9 months of age (window 9-11 months) in a randomized, community-based trial in rural Zimbabwe. *We hypothesize that IYCF-plus will improve the intake of key nutrients (protein, iron, zinc and folate) in 9-month-old infants compared to the standard-of-care IYCF intervention, and that IYCF-plus will increase length-for-age, weight-for-age, weight-for-length and haemoglobin more than IYCF (all secondary outcomes).*

Objective 3. Evaluate the impact of IYCF-plus on biological barriers to nutrient uptake and utilization at 9 months of age (window 9-11 months) in a randomized, community-based trial in rural Zimbabwe. *We hypothesize that the IYCF-plus intervention will increase microbiota maturity, ameliorate EED, reduce systemic inflammation and improve innate immune function in children aged 9 months, compared to the standard-of-care IYCF intervention.*

For peer review only - http://bmjopen.bmj.com/site/about/guidelines.xhtml

Objective 4. Identify metabolic signatures of the IYCF-plus intervention in young children at 9 months of age (window 9-11 months) in a randomized, community-based trial in rural Zimbabwe. *We hypothesize that the IYCF-plus intervention will increase the concentrations of essential amino acids and choline at 9 months of age more than the standard-of-care IYCF intervention.*

Objective 5. Explore the acceptability and feasibility of the IYCF-plus intervention among participants and community stakeholders utilizing qualitative methodology. *Information from this assessment will be shared with policymakers to help design a larger roll-out of this intervention at district, provincial, or national level.*

Objective 6. Explore the extent to which women's empowerment influences IYCF practices and nutrition outcomes in rural smallholder agricultural households.

We hypothesize that infants of women scoring in the highest tertile of the Women's Empowerment Agriculture Index (WEAI) will have improved macro- and micronutrient intake compared to infants of women in the lowest tertile of the WEAI index.

Objective 7. Identify the extent of regional and international migration and movement (both rural-rural and rural-urban) at the household level, explore the type, frequency and impact of any associated remittance flows on food consumption and production, and consider the importance of migration to any changes in established food cultures. *Information from this assessment will be shared with policymakers to help design a larger roll-out of this intervention at district, provincial, or national level.*

For peer review only - http://bmjopen.bmj.com/site/about/guidelines.xhtml

RATIONALE FOR INTERVENTIONS

The CHAIN trial will compare IYCF as tested in the SHINE trial[7] versus an enhanced IYCF intervention ("IYCF-plus"). IYCF comprises a set of sequential behaviour-change modules focusing on improved IYCF practices (e.g., nutrient density, feeding during illness, and dietary diversity), together with provision of daily SQ-LNS from 6-12 months of age, and powdered maize to make infant porridge. IYCF-plus comprises all the components of the IYCF intervention, plus four additional food supplements: pro-vitamin A (PVA) maize, NUA-45 sugar beans, moringa leaf powder, and whole egg powder. We have chosen this combination of 'functional' food supplements to close the remaining nutrient gaps for young children identified during SHINE (Table 1) and to ameliorate pathogenic pathways that impede uptake and utilization of nutrients.

Pro-Vitamin A (PVA) Maize is a bio-fortified maize rich in beta-carotenes, which is grown in Zimbabwe. Studies in neighbouring countries have shown that daily intake of PVA maize can improve the vitamin A status of children[15]. PVA maize appears less prone to contamination with aflatoxin, which is a fungal toxin affecting agricultural crops during growth, storage and processing that may impair child growth[16].

NUA 45 sugar beans are a high-nutrient bean variety providing bio-fortified zinc and iron, a high protein efficiency ratio, plus folate and resistant starch. Bio-fortified beans significantly increased haemoglobin, serum ferritin, and body iron in Rwandan women[17].

Moringa oleifera is a widespread crop in Zimbabwe. Dried moringa leaves can be ground into a powder providing a rich source of protein, fibre, mineral and micronutrients, including vitamin A, calcium, folate, vitamin C and vitamin E, and antioxidant polyphenols. Moringa

For peer review only - http://bmjopen.bmj.com/site/about/guidelines.xhtml

leaf powder is available in shops in Zimbabwe as a food supplement. Pilot studies show that moringa leaf powder is safe and widely accepted as a dietary supplement by children and caregivers in sub-Saharan Africa[18,19], but there have been no randomized trials.

Whole egg powder is commercially available, easily reconstituted and retains the nutrient content of whole eggs. Egg production is common in rural Zimbabwe. One egg per day for 6 months to children between 6-15 months of age increased LAZ by 0.63 in Ecuador[20]. This large effect is likely attributable to high-quality protein and choline, which are critical nutrients for linear growth[21 22]. Eggs contain all nine essential amino acids in proportions that closely match infant requirements for organ and muscle mass accretion. Choline is an essential nutrient that promotes growth in animal models[23]. Children with EED have reduced circulating concentrations of phosphatidylcholines[24], which are required for chondrogenesis at the growth plate, and reliant on adequate dietary intake of choline. One egg meets the majority of an infant's daily requirement. Eggs are also high in fat, energy-dense, and make modest but important contributions to vitamin A, iron, and zinc intake.

Together, these food supplements have the added plausible benefit of improving the microbiota and gut barrier function and reducing intestinal and systemic inflammation (Figure 1). The IYCF-plus intervention will increase dietary diversity and micronutrient content, which has been shown to promote healthy gut microbiota composition[25]. The resistant starch present in legumes is readily fermented by the gut microbiota to produce short-chain fatty acids, which act as a primary energy source for enterocytes, and other metabolites that maintain the integrity of the intestinal barrier[26]. Recent trials suggest that legumes modestly improve linear growth and intestinal permeability[27,28]; these effects may be enhanced through integration with other micronutrients such as vitamin A and zinc, which

For peer review only - http://bmjopen.bmj.com/site/about/guidelines.xhtml

can improve gut barrier function[29,30]. Legume intake and high dietary quality scores have been associated with reduced systemic inflammation[31]. Finally, pre-clinical studies have reported a role for moringa in reducing oxidative stress and improving immune function[32]. We hypothesize that children receiving the IYCF-plus intervention will have a more mature microbiota, reduced EED, less systemic inflammation and an associated improvement in anti-pathogen immune cell function compared to the IYCF intervention.

Ultimately, through improved agriculture and animal husbandry practices families could become self-sufficient in producing the beans, biofortified maize, moringa and eggs used for the IYCF-plus intervention for their household. As these foods are already grown locally on a small scale, there is also potential for local commercial production and processing of these products that would allow public distribution or purchasing instead of direct household-level production. Furthermore, these inputs have useful synergies: for example, moringa pods and maize bran provide food for chickens that improves their feed efficiency and increases egg production.

FORMATIVE RESEARCH AND COMMUNITY ENGAGEMENT

Formative work explored delivery and acceptability of the new food supplements proposed in CHAIN[33]. Briefly, this qualitative study purposively sampled nine community health workers (CHW) from the Shurugwi rural community where SHINE was conducted, in addition to 27 caregivers of children between 6-18 months of age. The aims of this formative work were to assess feasibility of delivering bean, egg and moringa powder to families; the acceptability of recipes devised to incorporate the new supplements into usual foods; and to inform IYCF-plus behavioural modules. The formative work also tested feasibility and uptake of this

For peer review only

multicomponent complementary feeding and behaviour change strategy among similar rural households to the study setting.

Activities included focus group discussions with mothers, group-based recipe formulation, testing and review as well as home visits to assess ingredient uptake, usage, storage and recipe adherence and innovations. Household observations and views from extended family members indicated high acceptability of the new ingredients. Sensory evaluation by mothers who formulated and standardised the recipes indicated high acceptability of the complementary food recipes. All formative study participants participated in developing the behaviour-change messages and finalization of the recipes in a recipe book developed for use in the CHAIN trial IYCF-plus intervention[33].

STUDY SETTING AND RECRUITMENT

The CHAIN trial was conducted in Shurugwi district, Zimbabwe. This is a predominantly rural, subsistence farming area, with 15% antenatal HIV prevalence and 35% stunting prevalence[7]. CHWs are a community-level cadre of healthcare workers within the Ministry of Health and Child Care. All CHWs in the study area underwent a two-week training on the interventions and have monthly supportive supervision meetings with study intervention nurses. Additionally spot checks on performance are conducted. They sensitized community stakeholders and individual families in their catchment area between birth and 5 months of age about the CHAIN study and refer those who are interested to the trial team. 282 infants turning 5 months of age from April 2021-August 2021 were identified through CHW registers. Consent visits were scheduled as close as possible to children turning 5 months old, and continued until the required sample size was reached. All children who fulfilled the inclusion and exclusion criteria during enrolment were eligible for the trial, including

For peer review only

households who had previously participated in the SHINE trial or the formative research with another child.

A research nurse visited the family's homestead to screen the child for eligibility, provide information on the trial, and undertake written informed consent with the parent/legal guardian in Shona or Ndebele. If the caregiver was not available, visits were rescheduled. All household members were encouraged to be present for consent and subsequent intervention and research visits. Interventions, delivered by CHWs, started as soon as possible after randomization, and the primary outcome is measured by research nurses at endline. Recruitment of study participants started on 26 April 2021 and study participants were followed until the end of February 2022.

TRIAL OUTCOMES

The primary outcome is energy intake in kcal at 9 months of age (visit window 9-11 months), as measured by multi-pass 24-hour dietary recall. Secondary and tertiary outcomes are defined in Table 2.

INCLUSION CRITERIA

Inclusion criteria were assessed by a screening questionnaire delivered to the primary caregiver by a research nurse.

- Individual level: Child age between 5-6 months
- Household level: Planning to live in the study area for the duration of the trial

EXCLUSION CRITERIA

- Severe infant disability that interferes with feeding
- Known allergy to peanuts or eggs

For peer review only - http://bmjopen.bmj.com/site/about/guidelines.xhtml

BASELINE DATA COLLECTION

Baseline data were collected via questionnaire on maternal, infant and household characteristics (Table 3). Maternal and infant height (ShorrBoard®), weight (Seca 874DR Mother-Baby scale), head circumference, and mid-upper arm circumference ShoreTape®) were measured. Maternal HIV status was collected by self-report and review of handheld records. Infant samples of stool, urine and blood were collected for laboratory analyses, including immunology, microbiome, and metabolic assays (full details below). Infant blood was collected by venepuncture into heparinised tubes (total 5.4 mL; maximum volume 1mL/kg), for centrifugation in the field laboratory to obtain plasma and peripheral blood cells for storage. One drop of blood was used to measure point-of-care haemoglobin, using a HemoCue 301 machine. Infant urine was collected by applying an adhesive urine bag to the infant's nappy area and waiting for the infant to pass urine during the visit. Urine was poured from the bag into a plain storage tube for transport to the field laboratory in a cool box. Infant stool was collected from the nappy into a plain tube and stored in a cool box for transport to the laboratory. If the infant did not pass stool during the visit, the mother was provided with a collection pack and instructions for how to collect the specimen the next morning, or as soon as possible thereafter, and keep the sample in a cool part of the house. The mother was asked to contact the study team once the sample has been collected and the research nurse visited the home to collect the sample. The research nurse checked the sample on arrival, labelled it with a barcode and placed it into a cool box for transport to the field laboratory. Children with symptomatic mild to moderate anaemia (<11 g/dL) or with severe anaemia (<7 g/dL) were referred to local clinics. Children with moderate or severe acute malnutrition (MUAC<125mm, or weight-for-length Z-score <-2) were also referred to local clinics.

For peer review only

RANDOMIZATION

The randomization schema was pre-prepared by the trial statistician using the RALLOC command in STATA 14, using random permuted blocks, with a 1:1 allocation to IYCF or IYCF-plus. Randomization codes are securely embedded in the trial database so that the next number is accessible to the data officer, but not the entire randomization list. Participant IDs were pre-generated and allocated to treatment arms prior to recruitment into the study. Participant IDs were assigned to a specific participant within a household after consent. Twins or eligible infants within the same household were allocated to the same trial arm. The CHW, supervised by an intervention nurse, visited the mother to tell her the trial allocation and to begin the interventions. It was not possible to blind households or fieldworkers to the interventions, but data and laboratory analysts are blinded to the allocated arm. All laboratory and data analyses will be identified by participant ID number, which does not contain details of the trial arm, and then merged by the trial statistician before reporting. Monthly reports of adverse events were reported to an independent trial safety monitor and to the Medical Research Council of Zimbabwe.

INTERVENTION DELIVERY

Behavioural modules: A total of nine interpersonal face-to-face counseling modules were delivered to caregivers in each arm by CHWs during 10 home visits, which coincide with key infant ages, so that sequential age-appropriate messages about complementary feeding are introduced and reinforced (Table 4). The CHW introduces the food supplements in both arms, demonstrates how to add them to food, monitors for any adverse reactions and provides monthly re-supplies. Modules are interactive and are delivered to all household members present. The last module was delivered at 11 months of age, with infant food supplements provided until 12 months of age, when all trial interventions end. Using this design, we

For peer review only

ensured that all infants are still receiving the IYCF or IYCF-plus interventions when endline data collection occurred at 9 months (window 9-11 months) of age.

If a module was missed, the CHW attempted to catch up by scheduling a new date, or else summarized the missed module at the next scheduled visit. If a caregiver moved within the study area, the CHW covering that area delivered modules to the caregiver, where possible; if the caregiver moved out of the study area, she did not receive study modules or food supplements. Endline data collection visits are conducted regardless of where the caregiver moves to. The estimated length of time exposed to the intervention is between eight and eleven months, from five to fifteen months. Median estimated length of time exposed to the intervention is eight months.

IYCF arm: Core IYCF counselling modules were complemented by provision of one sachet of 20g SQ-LNS daily between 6-12 months of age. SQ-LNS is a peanut-based supplement rich in calories, protein and micronutrients, which can be consumed directly from the sachet or mixed with porridge. Families also received a daily infant ration of white maize to feed the baby as porridge.

IYCF-plus arm: As in the IYCF arm, core counselling modules focusing on complementary feeding were delivered by the CHW, with provision of one sachet per day of 20g SQ-LNS between 6-12 months of infant age. In addition, families received NUA-45 biofortified bean powder, whole egg powder and moringa leaf powder for provision to the study child. The quantity of food supplements provided was based on the child’s age to ensure the daily recommended nutrient intake was met if the food supplement was consumed (Table 5). Families also received a daily infant ration of PVA biofortified maize to feed the baby as

For peer review only

porridge. Supplements were delivered in sealed containers, which the mother is asked to keep in a cool part of the house. Six recipes promoting high-quality staple foods, which were developed and standardized in the formative studies[33], are outlined in a recipe book, with cooking demonstrations given by the CHW.

INTERVENTION DELIVERY AND UPTAKE

Each CHW delivered the intervention to 1-3 enrolled households during the study. Eight intervention nurses (separate from the research nurses) were responsible for monitoring delivery of modules and food supplements and evaluating intervention uptake and compliance to recommended behaviours by caregivers. Intervention nurses did not provide counseling to mothers but did provide supportive supervision to CHWs by scheduled attendance at some household visits to provide feedback and by conducting unscheduled spot checks. Intervention nurses attended visits each time a CHW was delivering a module for the first time and additionally if needed. Intervention nurses held monthly meetings with the CHWs they supervise, to share learning, capture data on module delivery, and provide re-training as needed. A module delivery and intervention uptake checklist was completed by CHWs at each module delivery visit, and submitted to intervention nurses during monthly meetings. The checklist recorded modules that have been successfully delivered and dates of delivery. Data on uptake of interventions and compliance to recommended behaviours will include utilization of food supplements, any sharing of food supplements observed and involvement of other family members in child feeding assessed by a caregiver questionnaire.

FOLLOW-UP DATA COLLECTION

Households were visited by a research nurse at 9 months of infant age (window 9-11 months) for endline data collection. If the child was not present, the visit was rescheduled. Infant

For peer review only - http://bmjopen.bmj.com/site/about/guidelines.xhtml

weight, length, MUAC, and head circumference were measured, and samples of blood, urine and stool collected, using the same methods as at baseline. Children with illness were referred to local clinics, using the same criteria as at baseline.

The trial primary outcome was measured by research nurses via 24-hour multi-pass dietary recall**.** A subgroup of 50% of randomly selected household had a second 24-hour dietary recall visit approximately one week later. This method provides a robust and validated measure of nutrient intake based on a comprehensive and standardized assessment[34]. The dietary recall method assesses all food and beverages consumed in the previous 24 hours (including supplements provided by the trial) and comprises five passes. In the first pass, the research nurse asks the caregiver to list all foods consumed by the child during the last day, and to list any night feeds. In the second pass, the caregiver is asked to list all activities they undertook, and whether they fed the child food between activities; this helps the caregiver remember all feeding episodes. In the third pass, more details about foods and beverages are collected, including the time and place of preparation, ingredients, and brand of foods given. In the fourth pass, the caregiver estimates the portion size fed to the child. Research nurses carry samples of the most commonly consumed foods and ask the caregiver to estimate the amount fed to the child. The fieldworker then transfers the estimated portion to a standard cup, spoon or digital scale for recording. In the final pass, the caregiver recalls if there were any foods or meals that have not already been mentioned. Caregivers are also asked about the general health of the child on the previous day, whether the child's intake was less or more than usual, and how many times the child was breastfed.

Data from the 24-hour recall will be converted to observed energy and nutrient intakes by the following steps:

For peer review only - http://bmjopen.bmj.com/site/about/guidelines.xhtml

1. Ingredients and portion sizes were measured and weighed in grams where possible. For ingredients that could not be weighed, they will be converted to grams using locally collected data on food densities, supplemented with food density data from the FAO, USDA and NDSR [35-37].

2. Mixed dishes were disaggregated into ingredients and entered into Nutrisurvey to calculate nutrients in 100 grams of food.

3. Energy from each individual food/ingredient will be estimated using food composition data from regional food databases and USDA databases that have been collated for use in Zimbabwe over several studies[38].

Estimated energy and nutrient intakes will be compared with WHO-estimated energy requirements, the Institute of Medicine, Food and Nutrition Board recommended daily allowances for protein and choline, and the WHO-recommended nutrient intakes (RNIs) for other vitamins and minerals. For breastfeeding children, we will calculate the required nutrient intake from complementary foods by subtracting the amount of each nutrient in 550 g breast milk from the total requirement which is the estimated intake of breast milk for 9-11 month old children [39]. Energy requirements are calculated as kilojoules required per kilogram of body weight for breastfed children[40]; however, we will apply the slightly higher energy requirement estimated by Butte for children in low-income settings, which reflect increased needs due to greater infection burden[41]. Protein requirements will be defined as the WHO-recommended safe level of protein intake for 9-11 month old children[42]. Fat requirement will be defined as 35% energy requirement, which is the mid-point of several recommendations[43]. Micronutrient requirements will be defined as WHO-recommended nutrient intakes, except for calcium which is defined as the mean of the WHO RNI and US RNI. For zinc and iron,

For peer review only - http://bmjopen.bmj.com/site/about/guidelines.xhtml

we will assume 30% and 10% bioavailability, respectively[23,40,44,45]. For breastfed children, we will estimate the required nutrient intake from complementary foods by subtracting the amount of each nutrient in breast milk from the total requirement[45]. Using this comprehensive approach, we will determine the impact of the IYCF-plus versus IYCF intervention on energy intake (primary outcome) and the relative contributions of supplements (including SQ-LNS) and other complementary foods in closing infant nutrient gaps across trial arms. In addition to assessing total protein intake, we will explore essential amino acid intake, digestibility-adjusted protein intake and inflammation-adjusted protein intake[46].

TRAINING OF COMMUNITY HEALTH WORKERS, INTERVENTION AND RESEARCH NURSES

Eight intervention nurses (INs) underwent a two-week training on delivery of the trial interventions and provide supportive supervision to CHWs. All CHWs in the study area underwent a two-week training on the interventions. A training cascading approach was utilized where intervention nurses took part in training of CHWs, supporting other research staff. Monthly supportive-supervision cluster meetings with CHWs were conducted by INs. Four research nurses (Data Collectors- DCs) underwent a four-week training on conducting consenting, baseline, endline and 24-hour dietary recall interviews. Research nurses were trained separately from INs and all activities they carried out were conducted separately to avoid research bias.

DATA COLLECTION AND MANAGEMENT

Research data were collected onto electronic case report forms (CRFs) using pre-programmed tablets, with Open Data Kit (ODK) software. Full data validation procedures were programmed into the tablets including embedded skip patterns, data completeness and

For peer review only - http://bmjopen.bmj.com/site/about/guidelines.xhtml

plausibility checks. All data were checked daily by the field data officer, with implausible values verified or recollected. Back-up paper CRFs are carried by research nurses in the event of tablet failure. Data are uploaded from tablets onto a secure trial database daily and backed up onto a secure cloud database hosted on Microsoft Azure. Data will be stored for 20 years.

Each participant is allocated a participant identifier which is used on all forms to identify the child. Personal information and data are kept confidential and managed in accordance with the requirements of the Medical Research Council of Zimbabwe. Paper records (e.g., CRF, clinical / laboratory information and test results) will be entered into the electronic database; source documents will be stored in a secure, locked cupboard at each study site, and kept fully confidential. Data will be kept securely on a password-protected customised MS-SQL Server trial database and hosted by Microsoft Azure.

SAMPLE MANAGEMENT

Preprinted barcodes identifying the participant ID and sample type were adhered to collection tubes in the field, which were transported to the laboratory at room temperature (for blood) or in a cooler bag (for stool and urine samples). When samples arrived at the laboratory, they were processed and aliquoted into cryovials which were labeled with barcodes identifying the participant ID, sample type and aliquot number. Samples were stored in the field laboratory at -80° Celsius. At regular intervals, samples were transported to the main laboratory in Harare, where they were stored at -80° Celsius until analysis or shipment. All samples will be shipped to external laboratories on dry ice. Sample lists will be maintained in the main trial database. If participants consented to long-term storage, samples will be stored for up to 20 years.

For peer review only - http://bmjopen.bmj.com/site/about/guidelines.xhtml

SAMPLE SIZE

The sample size of 192 infants assumes 10% loss to follow-up due to withdrawal and infant deaths, meaning there will be an estimated 86 evaluable infants per group at endline. This sample size provides 86% power at 5% significance to detect a 20% increase in the proportion of infants achieving their recommended energy intake (by 24-hour dietary recall) in the IYCF-plus arm, assuming that only 65% of infants are meeting requirements in the IYCF (standard-of-care) arm based on SHINE data. If loss-to-follow is as high as 15%, we will still have 80% power to detect a 20% increase in the proportion reaching their daily energy intake (study primary outcome).

STATISTICAL ANALYSIS

Analysis of trial outcomes will be by intention-to-treat. P values will be 2-sided and interpreted as significant if $p<0.05$. Binary outcomes will be compared between groups using the Chi-square test and logistic regression to compute odds ratios and corresponding 95% confidence intervals. Other categorical outcomes with more than two levels will be compared between groups using Chi-squared tests and multinomial regression. Continuous outcomes will be compared using simple t-tests and linear regression. Non-normal continuous outcomes will be transformed appropriately before analysis. Robust standard error estimates will be used to estimate confidence intervals.

The primary outcome, percentage of infants meeting energy intake, will be calculated in two ways. First, we will use measured intake data from one dietary recall from all participants. Second, we will use the NCI method for calculated usual overall average intake. We will estimate the mean and percentiles of usual energy intake distributions

For peer review only - http://bmjopen.bmj.com/site/about/guidelines.xhtml

using the National Cancer Institute (NCI) method [47,48]. This method adjusts for measurement error – primarily due to day-to-day variability in intakes – in observed, single-day estimates of nutrient intakes. We will use the NCI macros, DISTRIB and MIXTRAN, plus bootstrapped standard errors for hypothesis testing [25, 27]. The MIXTRAN macro fits a mixed effects model of usual energy intake, and the DISTRIB macro uses a Monte Carlo procedure to estimate percentiles of the usual intake distribution. We will use the MIXTRAN macro to fit a model of energy intake with a fixed effect for intervention group and the DISTRIB macro to estimate usual energy intake distributions by treatment group. We will bootstrap the parameters to estimate the standard error of the mean usual intakes. We will use the point estimates and standard error estimates to construct 95% confidence intervals and calculate p-values for difference in mean by intervention group based on Welch's t-test. Bootstrapped standard errors will be calculated using bootstrap samples. When intake or the number of repeat recalls is low, it is common for the NCI method to fail to converge. In cases where convergence prohibits bootstrapping we will test hypotheses using the Wilcoxon testing for clustered method.

We will also interpret the mean intake of each nutrient for the primary and secondary analysis in each arm and will present mean differences with the 95% confidence interval for interpretation.

All analyses will be pre-specified in a Statistical Analysis Plan and posted online at Open Science Framework before analyses begin (https://osf.io/njy2a/). A per protocol analysis will be conducted using adherence data to pre-define the per protocol population. Adherence will be assessed by self-report in a 7-day recall. We will undertake two subgroup analyses: i) by

For peer review only - http://bmjopen.bmj.com/site/about/guidelines.xhtml

infant sex; and ii) by maternal HIV status, if we find some evidence of interactions with the intervention ($p<0.10$).

LABORATORY ANALYSES

Biological samples will be used to evaluate the nutrient profiles, EED, systemic inflammation, innate immune function, metabolic phenotype and the gut microbiota, as shown in Table 6.

EED and systemic inflammation: We will use a combination of plasma and stool ELISA assays to compare the impact of IYCF and IYCF-plus on EED, by characterising the hypothesised causal pathway from the gut to growth, measuring markers of intestinal inflammation (stool myeloperoxidase, neopterin) small intestinal damage (plasma I-FABP), intestinal permeability (alpha-1 antitrypsin), microbial translocation from the gut (plasma LBP, sCD14), systemic inflammation (AGP, CRP, TNFα) and growth hormone activity (plasma IGF-1). We will also measure aflatoxin M1 in urine, to assess recent exposure to dietary aflatoxin, which is a plausible cause of EED and may be reduced in the IYCF-plus arm since PVA maize appears less prone to fungal contamination.

Immune function: Immune cell activation is metabolically costly and chronic activation by recurrent infections/EED may create a barrier to children meeting their nutrient requirements by: i) driving inflammation and oxidative stress, ii) contributing to enteropathy, iii) compromising the capacity of innate immune cells to defend against new infections, which further deplete dietary nutrients. Multiple innate and adaptive immune mediators are dysregulated in undernourished children[49], but little is known about if/how nutritional interventions affect immune defences[50]. We will use whole blood samples to compare innate

For peer review only - http://bmjopen.bmj.com/site/about/guidelines.xhtml

immune cell phenotype and function between randomised groups. We will quantify surface expression of activation markers HLA-DR, CD64 and CD16 on blood monocytes, and CD64 and CD62L on blood monocytes and neutrophils via flow cytometry. To characterise the functional capacity of innate immune cells to respond to pathogen challenge we will quantify pro-inflammatory cytokine secretion in supernatants derived from whole blood cultures with and without bacterial lipopolysaccharide (LPS). Whole blood culture with and without fluorescent-labelled *Escherichia coli*-coated bioparticles will be used to quantify uptake of bacteria via flow cytometry.

Metabolic phenotyping: A targeted ultra-performance liquid chromatography-mass spectrometry (UPLC-MS)-based assay will be used to measure tryptophan-related metabolites in plasma (funding permitting)[51]. This includes metabolites involved in the kynurenine, serotonin, and indole pathways. In addition, downstream NAD+ related metabolites, such as nicotinic acid, nicotinamide, and nicotinamide-riboside will be measured together with markers of systemic inflammation (neopterin), enterocyte mass (citrulline) and the neurotransmitter dopamine. ^{1}H nuclear magnetic resonance (NMR) spectroscopy will be used to characterize the metabolic profiles of urine, plasma and faecal water samples. This approach measures H-containing metabolites present above the limit of detection in the samples in an untargeted manner. This captures information on amino acids, gut microbial metabolites, and metabolites involved in choline and energy metabolism. It may also be used to study dietary components and assess variation in their digestion.

Microbiome sequencing: Whole metagenome shotgun sequencing will be employed on stool samples to examine the effect of the trial interventions on the gut microbiome and its association with growth. DNA will be extracted from stool aliquots (200mg) using the

For peer review only - http://bmjopen.bmj.com/site/about/guidelines.xhtml

Qiagen PowerFecal Pro DNA kit, followed by metagenomic sequencing library preparation. Following qualitative and quantitative assessment of sequencing libraries, sequencing will be performed via the HiSeq 2500 platform producing 6-10 million sequencing reads per sample. Following quality control and trimming of human reads, sequencing reads will be processed through validated pipelines to generate compositional (MetaPhlAn v.3) and functional (HUMAnN v.3) readouts of the gut microbiome. Microbiome maturity will be assessed as previously described using a control dataset generated from the SHINE trial[52]. Aliquots of stool stored in glycerol will be used to isolate microorganisms of interest for downstream experiments assessing the influence of the gut microbiome on EED and growth.

QUALITATIVE STUDIES

The first qualitative substudy will develop a more in-depth understanding of how the CHAIN interventions were integrated into household nutrition practices, and the cultural, economic, and social processes that shape this. Up to 20 families were purposively sampled based on type of household, caregiver characteristics and trial arm. Semi-structured interviews were conducted to explore a range of themes, including any changes in the participants' receptiveness to the food supplements or children's response to them, the ability to maintain compliance with the food preparation guidelines, and challenges they may have experienced (e.g., accessing or storing the food supplements). The interviews also focus on relevant cultural, economic, and social processes operating at the household level that may influence food preparation and consumption practices and explore how these shape the sustainability of the intervention. Additionally, emphasis was placed on exploring the role that household and area-level gender dynamics may play in decision-making practices.

For peer review only - http://bmjopen.bmj.com/site/about/guidelines.xhtml

A second qualitative substudy will identify the ways in which household migration (defined here as people who have moved away from a household for 3 months or more) influences food security. 30 participating households were identified from the baseline survey as having at least one family member who has migrated across a range of geographical scales (local, regional, national, and international) and where possible representing different household types (including, male- or female-headed households, orphan-headed households, elder-headed households). In-depth interviews were conducted with study participants to explore the interactions that exist between household migration and remittance practices (receiving and sending) and their potential to influence household food consumption and production practices. In addition, the in-depth interviews aim to consider the importance of geographical scale to remitting practices and their influence on food consumption and production and investigate possible interactions between migration and household participation in the CHAIN intervention.

Interviews and focus groups were audio recorded, for subsequent transcription and translation; transcripts will be entered into NVivo for coding, analysis, and interpretation. Analysis will proceed using both deductive and inductive approaches and will utilise the framework method often employed in multidisciplinary health-related research. All audio recordings will be destroyed, although transcriptions will be stored securely and made available for future analysis as required.

TRIAL RISKS AND ADVERSE EVENTS

All interventions are commercially available foods (beans, egg powder, moringa, maize) or food supplements that are widely used globally (SQ-LNS). Peanuts and eggs are both staple foods in rural Zimbabwe; although both are potentially allergenic among infants in high-

For peer review only - http://bmjopen.bmj.com/site/about/guidelines.xhtml

income settings, the prevalence of food allergies in sub-Saharan Africa is extremely low[5,53,54]. SQ-LNS, which contains peanuts, was used in the same community in the SHINE trial among more than 2000 infants. From over 365,000 doses of SQ-LNS that were given to infants in SHINE, there were no allergic reactions, and no serious adverse events[5]. Only two adverse events were possibly or probably related to SQ-LNS; both resolved without sequelae[5]. Children with a known allergy to peanuts or eggs will be excluded from the study to further minimise the risk of adverse events. All adverse events will be reported and reviewed by the study physician, and a tabulated monthly summary will be sent to an independent safety monitor. All serious adverse events and trial-related adverse events will be reported to the Medical Research Council of Zimbabwe according to established timeframes.

The COVID-19 pandemic poses a potential risk to research activities in the community**.** We ensured that research staff wear personal protective equipment and practice physical distancing to keep themselves and research participants safe. All staff were trained in COVID protocols. Interviews were conducted in a confidential outdoor part of the homestead wherever possible. CHWs conduct their activities with guidance for safe working from the Ministry of Health and Child Care. Procedures were reviewed as the pandemic progresses, and any changes discussed with the District Health Executive and with the Medical Research Council of Zimbabwe. The food supplements are stopped at 12-15 months and not continued after the trial.

ETHICS

Ethical approval was granted by the Medical Research Council of Zimbabwe (MRCZ/A/2679), and Sponsorship provided by Queen Mary University of London (Joint Management Research Office, http://www.jrmo.org.uk/). All caregivers/legal guardians

For peer review only - http://bmjopen.bmj.com/site/about/guidelines.xhtml

provided written informed consent on behalf of their child. Information and consent procedures were administered in each individual's language of choice (Shona, Ndebele and/or English). Because mothers often need to consult with other family members before deciding about their child's participation in a trial, we include other family members in consent discussions if the mother wishes. If the caregiver wanted to think about participation or consult, the consenting visit was rescheduled to give her time. Illiterate mothers who understand a verbal explanation of the study can provide a thumb imprint on the consent form in the presence of an independent witness. Mothers aged between 15-18 years are considered emancipated minors under Zimbabwean law and could consent on behalf of their child. Families were provided with a small gift (soap and Vaseline) for each research data collection visit. Consent forms are available online (https://osf.io/njy2a/).

Since the IYCF intervention in the SHINE trial led to 20% reduction in stunting[5], we are providing IYCF as the enhanced standard-of-care for the control arm, since we believe it is unethical not to provide this intervention in the exact same population. Provision of food supplements may confer benefits for infant nutrition and growth, and for household wellbeing, but we believe it is ethically justifiable to randomize the supplements of powdered egg, NUA45 sugar beans, moringa and PVA maize, since none are routinely provided to households in the community and there is scientific equipoise as to whether these food supplements will bring additional benefit to what is already being provided in the IYCF arm.

DISSEMINATION

Trial results will be presented at international conferences and published in open-access journals. Data will be available on request, after publication of the primary trial findings, by contacting the Trial Management Group, with details of data access requests available on the

For peer review only - http://bmjopen.bmj.com/site/about/guidelines.xhtml

Zvitambo website. Results will be presented to the Ministry of Health and Child Care in Zimbabwe and will be disseminated to the study district through the Community Engagement Advisory Board which comprises peers selected by the community to review ongoing research studies in the community. Results will also be presented to UNICEF to inform the design and scale-up of IYCF programmes in Zimbabwe.

PATIENT AND PUBLIC INVOLVEMENT

Research questions and outcome measures were informed from gaps identified in the SHINE trial, incorporating the priorities, experiences, and preferences of community members. Families who participated in the formative study informed the design of the CHAIN trial and the burden of the intervention. Trial findings will be disseminated to participants through community engagement meetings.

DISCUSSION

Stunting remains a global health challenge which hinders human capital and perpetuates poverty. There is an urgent need for more efficacious nutrition-specific interventions to enhance child linear growth during complementary feeding. Currently, multiple barriers constrain nutrient intake, uptake and utilization, including marginal diets, EED, perturbations of the microbiome, metabolic dysregulation and chronic inflammation. Current IYCF approaches partly close nutrient gaps but require optimisation to fully restore healthy child growth. Utilising locally available foods with functional properties to supplement current IYCF approaches could have the dual goal of closing nutrient gaps in infancy and ameliorating pathogenic barriers to nutrient uptake and utilisation. We will test these ideas using a rigorous proof-of-concept trial design in a rural, subsistence farming community with a high burden of stunting, and evaluate acceptability, feasibility and sustainability of the

For peer review only

approach for longer-term and larger-scale deployment. Identifying new, sustainable ways to improve dietary quality and reduce stunting would help to accelerate progress towards 2030 global targets, and could have major benefits for long-term health, development and human capital.

DECLARATIONS

Ethics approval and consent to participate: Ethical permission has been granted by the Medical Research Council of Zimbabwe (MRCZ/A/2679). All mothers/caregivers will provide written informed consent on behalf of their child to participate.

Authors' contributions: Trial design: LES, CDB, RCR, NVT, DTC, JC, TN, TB, KD, KM, JS, PK, RN, AJP. Secured funding: LES, CDB, RCR, NVT, JC, TN, TB, KD, KM, JS, PK, RN, AJP. Agriculture design and expertise: JC, TN. Laboratory design and methods development: CDB, RCR, KM, JS, PK, AJP. Data management and analysis plan: BM, BC, RN. Formative work: LES, DTC, SF, NVT, LFL, TB, KD, BM, DC, AJP. Qualitative design and expertise: LES, TB, KD, DTC, SF, EM, NVT, LFL, MM. Training and implementation: AT, BM, DTC, KM. Study oversight: LES, JC, LFL, DTC, RN, AJP. All authors read and approved the final manuscript.

For peer review only

Acknowledgements: We appreciate the families in the formative study and the Shurugwi Community Engagement Advisory Board who helped in the design and conduct of the trial.

Funding: UK Research and Innovation Biotechnology and Biological Sciences Research Council (UKRI-BBSRC), from the Global Challenges Research Fund (GCRF) under Food & Nutrition Research for Health in the Developing World: Bioavailability and Nutrient Content.

RCR and AJP are funded by Wellcome (grants 206455/Z/17/Z and 108065/Z/15/Z). CDB is funded by a joint award from Wellcome and the Royal Society (206225/Z/17/Z). The second qualitative sub-study is funded by the Arts and Humanities Research Council (AHRC), UK (AH/T004428/1). Funding bodies had no role in the study design, implementation, analysis and interpretation of the data. The principal investigators had no financial and other competing interests.

Competing interests: The authors declare that they have no competing interests.

For peer review only

REFERENCES

1. Black RE, Victora CG, Walker SP, et al. Maternal and child undernutrition and overweight in low-income and middle-income countries. *Lancet*. Aug 3 2013;382(9890):427-451. doi:10.1016/S0140-6736(13)60937-X

2. UNICEF: Levels and trends in child malnutrition, United Nations Children's fund (UNICEF). World Health Organization, International Bank for Reconstruction and Development/The World Bank Levels and trends in child malnutrition: key findings of the 2019 edition of the joint child malnutrition estimates Geneva: World Health Organization 2019.

3. Prendergast AJ, Humphrey JH. The stunting syndrome in developing countries. *Paediatr Int Child Health*. Nov 2014;34(4):250-65. doi:10.1179/2046905514Y.0000000158

4. Dewey KG, Adu-Afarwuah S. Systematic review of the efficacy and effectiveness of complementary feeding interventions in developing countries. *Maternal & child nutrition*. Apr 2008;4 Suppl 1:24-85. doi:10.1111/j.1740-8709.2007.00124.x

5. Humphrey JH, Mbuya MNN, Ntozini R, et al. Independent and combined effects of improved water, sanitation, and hygiene, and improved complementary feeding, on child stunting and anaemia in rural Zimbabwe: a cluster-randomised trial. *Lancet Glob Health*. Jan 2019;7(1):e132-e147. doi:10.1016/S2214-109X(18)30374-7

6. Matare CR, Mbuya MN, Dickin KL, et al. Maternal Capabilities are associated with child caregiving behaviors among women in rural Zimbabwe. *The Journal of Nutrition*. 2020;

7. Humphrey J, Mbuya M, Ntozini R. Sanitation Hygiene Infant Nutrition Efficacy (SHINE) Trial Team. Independent and combined effects of improved water, sanitation, and hygiene, and improved complementary feeding, on child stunting and anaemia in rural Zimbabwe: a cluster-randomised trial. *Lancet Glob Health*. 2019;7:e132-47.

8. Rogawski McQuade ET, Platts-Mills JA, Gratz J, et al. Impact of water quality, sanitation, handwashing, and nutritional interventions on enteric infections in rural Zimbabwe: the Sanitation Hygiene Infant Nutrition Efficacy (SHINE) Trial. *The Journal of infectious diseases*. 2020;221(8):1379-1386.

9. Gough EK, Moulton LH, Mutasa K, et al. Effects of improved water, sanitation, and hygiene and improved complementary feeding on environmental enteric dysfunction in children in rural Zimbabwe: A cluster-randomized controlled trial. *PLoS Negl Trop Dis*. Feb 2020;14(2):e0007963. doi:10.1371/journal.pntd.0007963

10. Mutasa K, Ntozini R, Mbuya MNN, et al. Biomarkers of environmental enteric dysfunction are not consistently associated with linear growth velocity in rural Zimbabwean infants. *Am J Clin Nutr*. Mar 19 2021;doi:10.1093/ajcn/nqaa416

11. Gough EK, Moulton LH, Mutasa K, et al. Effects of improved water, sanitation, and hygiene and improved complementary feeding on environmental enteric dysfunction in children in rural Zimbabwe: A cluster-randomized controlled trial. *PLoS neglected tropical diseases*. 2020;14(2):e0007963.

12. Desai A, Smith LE, Mbuya MN, et al. The SHINE trial infant feeding intervention: pilot study of effects on maternal learning and infant diet quality in rural Zimbabwe. *Clinical Infectious Diseases*. 2015;61(suppl_7):S710-S715.

13. Robertson RC, Manges AR, Finlay BB, Prendergast AJ. The human microbiome and child growth–first 1000 days and beyond. *Trends in microbiology*. 2019;27(2):131-147.

For peer review only - http://bmjopen.bmj.com/site/about/guidelines.xhtml

14. Prendergast AJ, Rukobo S, Chasekwa B, et al. Stunting is characterized by chronic inflammation in Zimbabwean infants. *PLoS One*. 2014;9(2):e86928. doi:10.1371/journal.pone.0086928
15. Palmer AC, Craft NE, Schulze KJ, et al. Impact of biofortified maize consumption on serum carotenoid concentrations in Zambian children. *Eur J Clin Nutr*. Feb 2018;72(2):301-303. doi:10.1038/s41430-017-0054-1
16. Smith L, Stoltzfus R, Prendergast A. Food-Chain Mycotoxin Exposure, Gut Health and Impaired Growth: A conceptual framework *Advances in Nutrition*. 2012;3:1-6.
17. Haas JD, Luna SV, Lung'aho MG, et al. Consuming Iron Biofortified Beans Increases Iron Status in Rwandan Women after 128 Days in a Randomized Controlled Feeding Trial. *J Nutr*. Aug 2016;146(8):1586-92. doi:10.3945/jn.115.224741
18. Barichella M, Pezzoli G, Faierman SA, et al. Nutritional characterisation of Zambian Moringa oleifera: acceptability and safety of short-term daily supplementation in a group of malnourished girls. *Int J Food Sci Nutr*. Feb 2019;70(1):107-115. doi:10.1080/09637486.2018.1475550
19. Boateng L, Nyarko R, Asante M, Steiner-Asiedu M. Acceptability of Complementary Foods That Incorporate Moringa oleifera Leaf Powder Among Infants and Their Caregivers. *Food and nutrition bulletin*. Mar 2018;39(1):137-148. doi:10.1177/0379572117708656
20. Iannotti LL, Lutter CK, Stewart CP, et al. Eggs in Early Complementary Feeding and Child Growth: A Randomized Controlled Trial. *Pediatrics*. Jul 2017;140(1)doi:10.1542/peds.2016-3459
21. Semba RD, Shardell M, Sakr Ashour FA, et al. Child Stunting is Associated with Low Circulating Essential Amino Acids. *EBioMedicine*. Apr 2016;6:246-252. doi:10.1016/j.ebiom.2016.02.030
22. Ghosh S, Suri D, Uauy R. Assessment of protein adequacy in developing countries: quality matters. *Br J Nutr*. Aug 2012;108 Suppl 2:S77-87. doi:10.1017/S0007114512002577
23. Zeisel SH, Blusztajn JK. Choline and human nutrition. *Annu Rev Nutr*. 1994;14:269-96. doi:10.1146/annurev.nu.14.070194.001413
24. Baron J, Savendahl L, De Luca F, et al. Short and tall stature: a new paradigm emerges. *Nat Rev Endocrinol*. Dec 2015;11(12):735-46. doi:10.1038/nrendo.2015.165
25. Heiman ML, Greenway FL. A healthy gastrointestinal microbiome is dependent on dietary diversity. *Mol Metab*. May 2016;5(5):317-320. doi:10.1016/j.molmet.2016.02.005
26. Kelly CJ, Zheng L, Campbell EL, et al. Crosstalk between Microbiota-Derived Short-Chain Fatty Acids and Intestinal Epithelial HIF Augments Tissue Barrier Function. May 13 2015;17(5):662-71. doi:10.1016/j.chom.2015.03.005
27. Agapova SE, Stephenson KB, Divala O, et al. Additional Common Bean in the Diet of Malawian Children Does Not Affect Linear Growth, but Reduces Intestinal Permeability. *J Nutr*. Feb 1 2018;148(2):267-274. doi:10.1093/jn/nxx013
28. Stephenson KB, Agapova SE, Divala O, et al. Complementary feeding with cowpea reduces growth faltering in rural Malawian infants: a blind, randomized controlled clinical trial. *The American journal of clinical nutrition*. Dec 2017;106(6):1500-1507. doi:10.3945/ajcn.117.160986
29. de Medeiros P, Pinto DV, de Almeida JZ, et al. Modulation of Intestinal Immune and Barrier Functions by Vitamin A: Implications for Current Understanding of Malnutrition and Enteric Infections in Children. *Nutrients*. Aug 21 2018;10(9)doi:10.3390/nu10091128
30. Wapnir RA. Zinc deficiency, malnutrition and the gastrointestinal tract. *J Nutr*. May 2000;130(5S Suppl):1388S-92S. doi:10.1093/jn/130.5.1388S

For peer review only - http://bmjopen.bmj.com/site/about/guidelines.xhtml

31. Esmaillzadeh A, Azadbakht L. Legume consumption is inversely associated with serum concentrations of adhesion molecules and inflammatory biomarkers among Iranian women. *J Nutr*. Feb 2012;142(2):334-9. doi:10.3945/jn.111.146167
32. Abdel-Latif M, El-Shahawi G, Aboelhadid SM, Abdel-Tawab H. Modulation of murine intestinal immunity by Moringa oleifera extract in experimental hymenolepiasis nana. *J Helminthol*. Mar 2018;92(2):142-153. doi:10.1017/S0022149X17000293
33. Chagwena DT, Fernando S, Tavengwa NV, et al. Formulation and acceptability of local nutrient-dense foods for young children: A formative study for the Child Health Agriculture Integrated Nutrition (CHAIN) Trial in rural Zimbabwe. *Under Press*.
34. Conway JM, Ingwersen LA, Moshfegh AJ. Accuracy of dietary recall using the USDA five-step multiple-pass method in men: an observational validation study. *Journal of the American Dietetic Association*. Apr 2004;104(4):595-603. doi:10.1016/j.jada.2004.01.007
35. Schakel SF. Maintaining a nutrient database in a changing marketplace: keeping pace with changing food products—a research perspective. Elsevier; 2001. p. 315-322.
36. Charrondiere U, Haytowitz D, Stadlmayr B. FAO/INFOODS density database, version 2.0. 2012:
37. US Department of Agriculture ARS, Nutrient Data Laboratory. USDA National Nutrient Database for Standard Reference, Release 27 (revised). http://www.ars.usda.gov/ba/bhncr/ndl
38. Merchant AT, Dehghan M, Chifamba J, Terera G, Yusuf S. Nutrient estimation from an FFQ developed for a black Zimbabwean population. *Nutrition Journal*. 2005/12/13 2005;4(1):37. doi:10.1186/1475-2891-4-37
39. Brown K, Dewey K, Allen L. Complementary feeding of young children in developing countries: a review of current scientific knowledge. 1998;
40. University UN, Organization WH. *Human Energy Requirements: Report of a Joint FAO/WHO/UNU Expert Consultation: Rome, 17-24 October 2001*. Food & Agriculture Org.; 2004.
41. Butte NF. Energy requirement of infants. *Public Health Nutrition*. 2009 2009;8(7A):953-967. In File.
42. WHO J. Protein and amino acid requirements in human nutrition. *World Health Organization technical report series*. 2007;(935):1.
43. Uauy R, Castillo C. Lipid requirements of infants: implications for nutrient composition of fortified complementary foods. *The Journal of nutrition*. 2003;133(9):2962S-2972S.
44. Micronutrients IoMPo. *Dietary Reference Intakes for Energy, Carbohydrate, Fiber, Fat, Fatty Acids, Cholesterol, Protein and Amino Acids. Panel on Macronutrients Panel on the Definition of Dietary Fiber, Subcommittee on Upper Reference Levels of Nutrients, Subcommittee on Interpretation and Uses of Dietary Reference Intakes, and the Standing Committee on the Scientific Evaluation of Dietary Reference Intakes, Food and Nutrition Board*. National Academies Press; 2005.
45. Dewey KG, Brown KH. Update on technical issues concerning complementary feeding of young children in developing countries and implications for intervention programs. *FOOD AND NUTRITION BULLETIN-UNITED NATIONS UNIVERSITY-*. 2003;24(1):5-28.
46. Callaghan M, Oyama M, Manary M. Sufficient protein quality of food aid varies with the physiologic status of recipients. *The Journal of nutrition*. 2017;147(3):277-280.
47. Intakes UD. SAS Macros for the NCI Method. 2012.

For peer review only - http://bmjopen.bmj.com/site/about/guidelines.xhtml

48. Tooze JA, Kipnis V, Buckman DW, et al. A mixed-effects model approach for estimating the distribution of usual intake of nutrients: the NCI method. *Statistics in medicine*. 2010;29(27):2857-2868.
49. Rytter MJH, Kolte L, Briend A, Friis H, Christensen VB. The immune system in children with malnutrition—a systematic review. *PloS one*. 2014;9(8):e105017.
50. Bourke CD, Jones KD, Prendergast AJ. Current understanding of innate immune cell dysfunction in childhood undernutrition. *Frontiers in immunology*. 2019;10:1728.
51. Whiley L, Nye LC, Grant I, et al. Ultrahigh-Performance Liquid Chromatography Tandem Mass Spectrometry with Electrospray Ionization Quantification of Tryptophan Metabolites and Markers of Gut Health in Serum and Plasma-Application to Clinical and Epidemiology Cohorts. *Anal Chem*. Apr 16 2019;91(8):5207-5216. doi:10.1021/acs.analchem.8b05884
52. Subramanian S, Huq S, Yatsunenko T, et al. Persistent gut microbiota immaturity in malnourished Bangladeshi children. *Nature*. 2014;510(7505):417-421.
53. Amoah AS, Obeng BB, Larbi IA, et al. Peanut-specific IgE antibodies in asymptomatic Ghanaian children possibly caused by carbohydrate determinant cross-reactivity. *J Allergy Clin Immunol*. Sep 2013;132(3):639-647. doi:10.1016/j.jaci.2013.04.023
54. Wollmann E, Hamsten C, Sibanda E, et al. Natural clinical tolerance to peanut in African patients is caused by poor allergenic activity of peanut IgE. *Allergy*. Jun 2015;70(6):638-52. doi:10.1111/all.12592

For peer review only

Table 1. Nutrient provision in IYCF and IYCF-plus trial arms

Figure 1. Nutrient requirements from complementary food and provided by IYCF and IYCF-PLUS intervention diets with and without estimated mealie meal consumption for three age groups of children.
Red (fails to meet requirement), orange (meets >88% requirement), green (exceeds requirement).[1]

	6-8 mo			9-11 mo			12-24 mo		
Nutrient	Required from Complementary Food	IYCF	IYCF-PLUS	Required from Complementary Food	IYCF	IYCF-PLUS	Required from Complementary Food	IYCF	IYCF-PLUS
Energy (kcal) from supplement alone	270	118	222	451	118	247	746	118	272
Energy (kcal) from supplement + estimated mealie meal intake		243	347		368	497		618	772
Protein (g)	7.7	2.6	10.8	8.1	2.6	12.6	8.1	2.6	14.4
Fat (g)	0.8	9.6	15.7	7.6	9.6	15.8	18.0	9.6	16.0
Vitamin A (μg RE)	207	400	519	222	400	540	238	400	560
Folic Acid (μg)	48	80	132	50	80	160	133	80	188
Calcium (mg)	232	280	375	246	280	438	360	280	502
Iron (mg)	9.2	6.0	9.0	9.2	6.0	10.4	5.7	6.0	11.9
Zinc (mg)	3.8	8.0	9.1	3.9	8.0	9.5	3.8	8.0	9.8
Choline (mg)	47	13	145	55	13	152	114	13	158

[1]Nutrient requirements from complementary food calculated as total requirement less provided in breastmilk. Estimated mealie meal consumption is 125 kcal, 250 kcal, and 500 kcal based on observed intakes during SHINE trial. IYCF diet includes SHINE IYCF behavior change modules + 20 g Nutributter per day. IYCF-PLUS diet includes IYCF behaviour change modules + 20 g Nutributter per day + whole egg powder, moringa powder, and iron and zinc-fortified sugar bean powder.

For peer review only - http://bmjopen.bmj.com/site/about/guidelines.xhtml

Table 2 – Trial outcomes at 9 months of age (window 9-11 months)

Endpoint	Definition
Energy intake	Percentage of infants meeting daily energy requirements at 9 months of age (window 9-11 months), measured by multi-pass 24-hour dietary recall.
Protein, iron, zinc and folate intake	Percentage of infants meeting daily protein, iron, zinc and folate requirements at 9 months of age, measured by multi-pass 24-hour dietary recall.
Length-for-age Z-score	Length-for-age expressed as a Z-score compared to the WHO 2006 reference median
Weight-for-age Z score	Weight-for-age expressed as a Z-score compared to the WHO 2006 reference median
Weight-for-length Z score	Weight-for-length expressed as a Z-score compared to the WHO 2006 reference median
Haemoglobin	Concentration of haemoglobin (in g/dL) in a whole blood sample, measured by HemoCue point-of-care assay and adjusted for altitude
Microbiome maturity	Microbiota-for-age Z-score

For peer review only

Environmental enteric dysfunction (EED)	Biomarkers of intestinal inflammation (faecal neopterin and myeloperoxidase), small intestinal damage (plasma I-FABP and citrulline), intestinal permeability (faecal A1AT), microbial translocation (plasma sCD14, LBP), systemic inflammation (plasma CRP, AGP, TNF-alpha and K:T ratio) and growth hormone axis (IGF-1)
Innate immune cell phenotype	Surface marker expression by peripheral blood monocytes and neutrophils
Innate immune cell function	Surface marker expression and cytokine secretion from innate immune cells challenged with lipopolysaccharide *in vitro* relative to unstimulated controls<br><br>Capacity of innate immune cells to internalise bacteria *in vitro*
Plasma essential amino acids	Plasma concentrations of phenylalanine, valine, threonine, tryptophan, methionine, leucine, isoleucine, lysine and histidine, as measured by LC-MS-MS

For peer review only - http://bmjopen.bmj.com/site/about/guidelines.xhtml

Plasma choline	Plasma concentration of choline, as measured by LC-MS-MS
Urinary metabolic signature	Global untargeted metabolomic phenotyping undertaken by ^{1}H nuclear magnetic resonance spectroscopy

For peer review only

For peer review only - http://bmjopen.bmj.com/site/about/guidelines.xhtml

Table 3 – CHAIN trial schedule[a]

**Procedure**	**Visit 1[b] Screening and enrolment**		**Visit 2 Baseline**	**Visit 3[c] Endline**
Screening and eligibility check *Form 2*	x	**Randomisation and start of monthly IYCF or IYCF-plus interventions**		
Informed consent *Form 3 and 4*	x			
Locator and contact information *Form 5*	x		x	x
Baseline interview *Form 6*			x	
Maternal weight, height and MUAC			x	
Infant weight, height, MUAC			x	x

For peer review only

and head circumference				
Infant blood collection[d]			x	x
Infant haemoglobin			x	x
Infant stool collection[c]			x	x
Infant urine collection[d]			x	x
Endline interview *Form 9*				x
24-hour dietary recall[e] *Form 10*				x
Informed consent for qualitative sub-studies [f] *Form 11*				
Qualitative sub-study 1 guide *Form 12a*				x

For peer review only

Informed consent for qualitative sub studies *Form 11* [g]				
Qualitative sub-study 2 guide *Form 12b*				x

[a]Trial enrolment began April, 2021 and concluded in August, 2021.

[b]For mothers who wish to provide informed consent on a subsequent day to screening, these visits will be separated.

[c]Target date 9 months of infant age (visit window 9-11 months or 274-334 days)

[d]If any specimens cannot be collected during the visit (e.g. if the infant fails to pass stool), the specimen collection will be rescheduled for the next day, or as soon as possible after the visit. Rarely it may be necessary to repeat a specimen collection if the sample was insufficient, or fails quality control checks during processing in the laboratory. All blood draws will be kept to a safe limit, defined as maximum of 1ml/kg body weight.

[e]The primary outcome, measured by multi-pass 24-hour dietary recall, will be repeated 1 week later in a sub-sample of 50% infants for methods validation.

[f]Sub-group of up to 20 purposively selected households. The social scientist will visit the family during a separate visit at 7-9 months (214-273 days) of infant age, and obtain separate written informed consent for the qualitative interviews.

For peer review only

[g]Sub-group of up to 30 purposively selected households. The social scientist will obtain written informed consent for the qualitative interviews regarding migration, which will be conducted throughout the course of the study.

For peer review only

Table 4 – Module delivery

**Infant age (window period)[a]**	**Home Visit**	**Infant age (window period)[a]**	**CONTROL ARM**<br>**IYCF modules and supplies**	**INTERVENTION ARM**<br>**IYCF-plus modules and supplies**
5 months (5 months-<6 months)	1	5 months (5 months-<6 months) (153 – 182 days)	MODULE 1<br>• Nutrition for your baby<br>• Composition and functions of SQ-LNS [Feeding as a chore or actual work; stomach capacity and graph on nutrient gap of breastmilk and mealie meal]	MODULE 1<br>• Nutrition for your baby + introduction of the 3 powders<br>• Composition and functions of SQ-LNS and the 3 powders [Feeding as chore or actual work; stomach capacity and graph on nutrient gap of breastmilk and mealie meal)
6 months (6 months-<7 months)	2	6 months (6 months-<7 months) (183 – 213 days)	MODULE 2<br>• Introducing solid foods<br>• Additional information on SQ-LNS<br>• Breastmilk + porridge + SQ-LNS	MODULE 2<br>• Introducing solid foods<br>• Additional information on SQ-LNS and the 3 foods *(Bean powder, Egg powder and Moringa powder)*<br>• Breastmilk + porridge + SQ-LNS
6 months 1 week (6	3	6 months 1 week (6 months 1	MODULE 2.1	MODULE 2.1<br>• Introduce Bean Powder

months 1 week- <7 months)		week- <7 months) (190 - 213 days)	• Frequency of complementary foods<br>• Reinforce messages<br>• Breastmilk + porridge + SQ-LNS	• Frequency of complementary foods<br>• Reinforce messages<br>• Breastmilk + porridge + SQ-LNS + bean powder
6 months 2 weeks (6 months 2 weeks- <7 months)	4	6 months 2 weeks (6 months 2 weeks- <7 months) (197 – 213 days)	MODULE 2.2<br>• Nutrient-dense complementary meals<br>• Reinforce messages<br>• Breastmilk + porridge+ SQ-LNS + nutrient density	MODULE 2.2<br>• Introduce Egg Powder<br>• Nutrient-dense complementary meals<br>• Reinforce messages<br>• Breastmilk +porridge+ SQ-LNS + bean powder + egg powder
6 months 3 weeks (6 months 3 weeks- <7 months)	5	6 months 3 weeks (6 months 3 weeks- <7 months) (204 – 213 days)	MODULE 2.3<br>• Complementary feeding schedule and family support<br>• Reinforce messages<br>• Breastmilk + porridge + SQ-LNS	MODULE 2.3<br>• Introduce Moringa<br>• Complementary feeding schedule and family support<br>• Reinforce messages<br>• Breastmilk + porridge + SQ-LNS + bean powder + egg powder + moringa powder

7 months (7 months- <8 months)	6	7 months (7 months- <8 months) (214 – 243 days)	MODULE 3<br>• Introducing more foods<br>• Reinforce messages<br>• Breastmilk + SQ-LNS + porridge (sadza/soup/potatoes/maheu+ other locally available foods e.g. vegetables etc.)	MODULE 3<br>• Introducing more foods<br>• Reinforce messages<br>• Breastmilk + SQ-LNS + bean powder + egg powder + moringa powder + porridge (sadza /soup/potatoes/maheu + other locally available foods e.g. vegetables etc.)
8 months (8 months- <9 months)	7	8 months (8 months- <9 months) (244 – 273 days)	MODULE 4<br>• Feeding during illness<br>• Reinforce messages<br>• Breastmilk + SQ-LNS + porridge (sadza/soup/potatoes/maheu+ other locally available foods e.g. vegetables etc.)	MODULE 4<br>• Feeding during illness<br>• Reinforce messages<br>• Breastmilk + SQ-LNS + bean powder + egg powder + moringa powder + porridge (sadza /soup/potatoes/maheu + other locally available foods e.g. vegetables etc.)
9 months (9 months- <10 months)	8	9 months (9 months- <10 months) (274 – 304 days)	MODULE 5<br>• Dietary Diversity<br>• Reinforce messages<br>• Dietary diversity<br>• Breastmilk + SQ-LNS + porridge	MODULE 5<br>• Dietary Diversity<br>• Reinforce messages<br>• Dietary diversity<br>• Breastmilk + SQ-LNS + bean powder + egg powder +

For peer review only - http://bmjopen.bmj.com/site/about/guidelines.xhtml

			(sadza/soup/potatoes/maheu+ other locally available foods e.g. vegetables etc.)	moringa powder + porridge (sadza /soup/potatoes/maheu + other locally available foods e.g. vegetables etc.)
10 months (10 months- <11 months)	9	10 months (10 months- <11 months) (305 – 334 days)	MODULE 6<br>• Reinforcing all Modules<br>• Breastmilk + SQ-LNS + porridge (sadza/soup/potatoes/maheu+ other locally available foods e.g. vegetables etc.)	MODULE 6<br>• Reinforcing all Modules<br>• Breastmilk + SQ-LNS + bean powder + egg powder + moringa powder + porridge (sadza /soup/potatoes/maheu + other locally available foods e.g. vegetables etc.)
11 months (11 months- <12 months)	10	11 months (11 months- <12 months) (345 – 365 days)	MODULE 6<br>• Reinforcing all Modules<br>• Breastmilk + SQ-LNS + porridge (sadza/soup/potatoes/maheu+ other locally available foods e.g. vegetables etc.)	MODULE 6<br>• Reinforcing all Modules<br>• Breastmilk + SQ-LNS + bean powder + egg powder + moringa powder + porridge (sadza /soup/potatoes/maheu + other locally available foods e.g. vegetables etc.)

[a] Each module session will be delivered for approximately 60 minutes. If a module is not delivered within the intervention window (i.e. appropriate infant age), the CHW will try to catch up by scheduling a new date as soon as possible. Each module will be delivered to the mother and her family. If the rescheduled module for modules 1.0 and 2.0 overlap, these two

modules will be delivered at the same time. If the rescheduled module overlaps with the next visit for other modules (2.0, 2.1, 2.2, 2.3 etc) the visits will be scheduled at least 3 days apart so that families have time to absorb the new material. Delivery of IYCF-plus modules has therefore been designed to be flexible following complementary feeding guidance. Experience from formative work showed that it is feasible to deliver the combined modules at once.

For peer review only

Table 5: Quantities of food supplements in the IYCF-plus arm

Infant Age Group (months)	Mealie Meal (PVA maize)[1]	SQ-LNS[2]	Whole egg powder[3]	Moringa leaf, dried and ground[4]	Sugar bean legume, finely ground[5]
**6-8**	≥ 42 g (3 Tbsp)	20 g	14 g (3 tsp)	5 g (1 tsp)	5 g (1 tsp)
**9-11**	≥ 71 g (4.5 Tbsp)	20 g	14 g (3 tsp)	10 g (2 tsp)	10 g (2 tsp)

Food supplements will be delivered monthly by CHWs. [1]Mealie Meal (PVA maize) will be provided in 500g bags, with 3 bags/month (1500g) between 6-8 months of age and 5 bags/month (2500g) between 9-11 months of age. Households in the IYCF arm will receive the same amount of mealie meal per month [2]SQ-LNS will be supplied monthly to ensure 1 x 20g sachet per day can be provided (30 or 31 sachets per month). [3]Whole egg powder will be delivered as a 500g bag per month. [4]Moringa leaf powder will be supplied in 175g bags, with 1 bag/month (175g) between 6-8 months of age and 2 bags/month (350g) between 9-11 months of age. [5]NUA 45 sugar bean powder will be supplied in 175g bags, with 1 bag/month (175g) between 6-8 months of age and 2 bags/month (375g) between 9-11 months of age. These quantities allow for 15% extra in case of spillage or sharing

For peer review only

For peer review only - http://bmjopen.bmj.com/site/about/guidelines.xhtml

Table 6 – Laboratory analyses

Sample type	Assay	Method	Location of work	Study subjects	Time-points
Plasma	I-FABP, CRP, AGP, TNFα, LBP, sCD14, IGF-1	ELISA	Zvitambo	All	Baseline, endline
Peripheral blood leukocytes	Innate immune cell phenotype[a]	Flow cytometry	Zvitambo	All	Baseline, endline
Whole blood	Whole Blood Culture with and without LPS	Cell culture, flow cytometry and ELISA	Zvitambo	All	Baseline, endline
Whole blood	Bacterial binding Assay	Cell culture, flow cytometry	Zvitambo	All	Baseline, endline
Stool	Myeloperoxidase, neopterin, alpha-1 antitrypsin	ELISA	Zvitambo	All	Baseline, endline
Urine	Global untargeted metabolomic phenotyping	^{1}H NMR spectroscopy	Southampton, UK	All	Baseline, endline
Plasma	Global untargeted metabolomic phenotyping	^{1}H NMR spectroscopy	Southampton, UK	All	Baseline, endline
Plasma	Kynurenine:tryptophan ratio, citrulline, essential	Ultrahigh-performance	Southampton, UK	All	Baseline, endline

For peer review only - http://bmjopen.bmj.com/site/about/guidelines.xhtml

	amino acids, choline	liquid chromatography tandem mass spectrometry with electrospray ionization			
Stool	Whole metagenome shotgun sequencing	Illumina HiSeq	Blizard Institute, UK	All	Baseline, endline
Stool	Metabolic phenotyping of fecal water	^{1}H NMR spectroscopy	Southampton, UK	All	Baseline, endline
Urine	Aflatoxin M1 and creatinine	ELISA	Zvitambo	All	Baseline, endline

[a] Expression of surface activation markers HLA-DR, CD64 and CD16 on monocytes and HLA-DR, CD64, CD16 and CD62L on neutrophils

I-FABP: Intestinal fatty acid binding protein; AGP: Alpha-1 acid glycoprotein; CRP: C-reactive protein; LBP: lipopolysaccharide binding protein; IGF-1: insulin like growth factor 1

For peer review only

List of Figures

Figure 1 – Hypothesized impact of IYCF-plus intervention on barriers to nutrient intake, uptake and utilization for healthy child growth

For peer review only

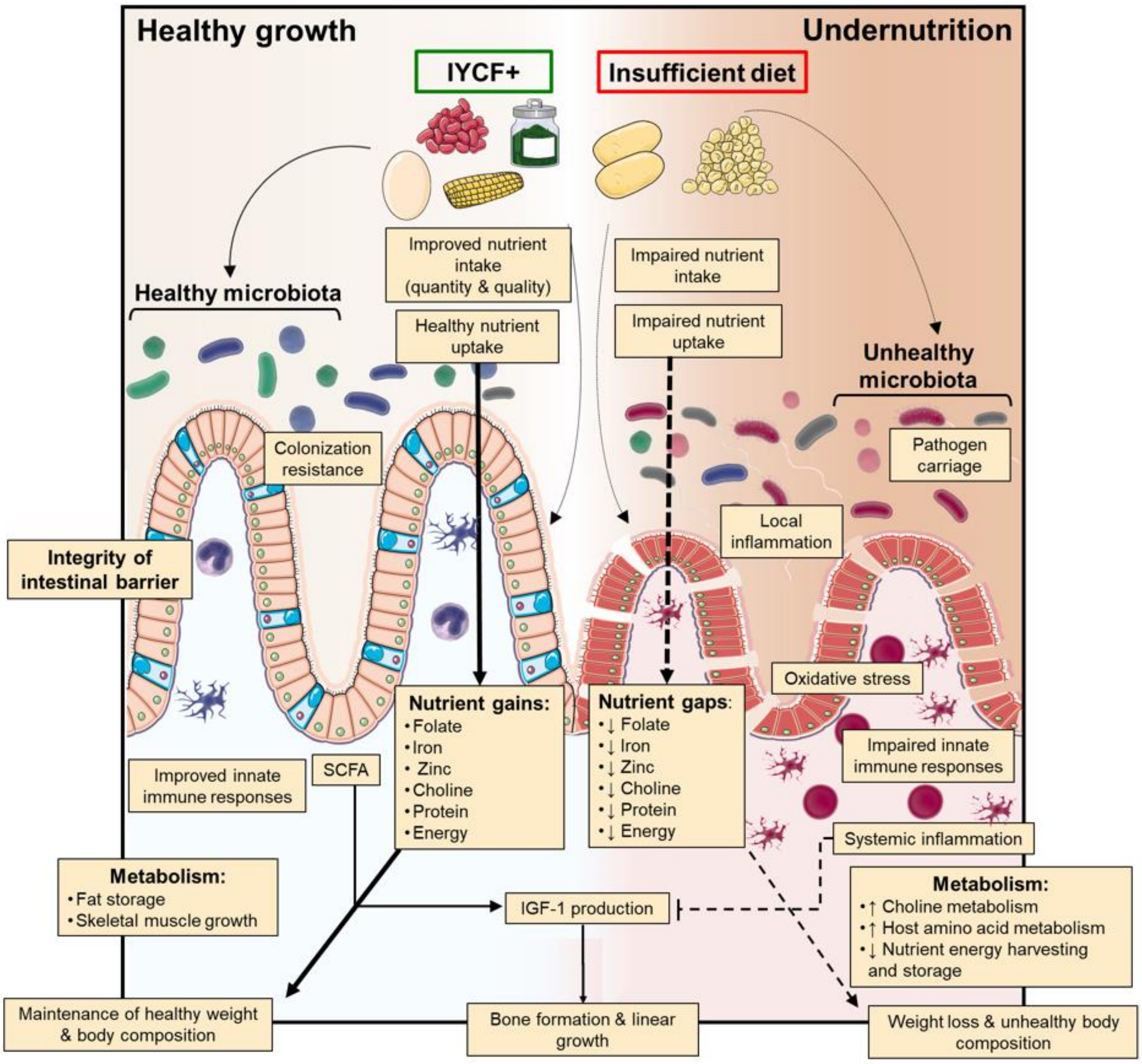
Healthy growth
Undernutrition
IYCF+
Insufficient diet
Improved nutrient intake (quantity & quality)
Healthy nutrient uptake
Impaired nutrient intake
Impaired nutrient uptake
Healthy microbiota
Unhealthy microbiota
Colonization resistance
Pathogen carriage
Local inflammation
Integrity of intestinal barrier
Oxidative stress
Nutrient gains:
• Folate
• Iron
• Zinc
• Choline
• Protein
• Energy
Nutrient gaps:
• ↓ Folate
• ↓ Iron
• ↓ Zinc
• ↓ Choline
• ↓ Protein
• ↓ Energy
Improved innate immune responses
SCFA
Impaired innate immune responses
Systemic inflammation
Metabolism:
• Fat storage
• Skeletal muscle growth
IGF-1 production
Metabolism:
• ↑ Choline metabolism
• ↑ Host amino acid metabolism
• ↓ Nutrient energy harvesting and storage
Maintenance of healthy weight & body composition
Bone formation & linear growth
Weight loss & unhealthy body composition

SPIRIT

STANDARD PROTOCOL ITEMS: RECOMMENDATIONS FOR INTERVENTIONAL TRIALS

SPIRIT 2013 Checklist: Recommended items to address in a clinical trial protocol and related documents*

**Section/item**	**Item No**	**Description**	**CHAIN**
**Administrative information**			
Title	1	Descriptive title identifying the study design, population, interventions, and, if applicable, trial acronym	1
Trial registration	2a	Trial identifier and registry name. If not yet registered, name of intended registry	3
	2b	All items from the World Health Organization Trial Registration Data Set	3
Protocol version	3	Date and version identifier	3
Funding	4	Sources and types of financial, material, and other support	x
Roles and responsibilities	5a	Names, affiliations, and roles of protocol contributors	1
	5b	Name and contact information for the trial sponsor	31
	5c	Role of study sponsor and funders, if any, in study design; collection, management, analysis, and interpretation of data; writing of the report; and the decision to submit the report for publication, including whether they will have ultimate authority over any of these activities	31

For peer review only - http://bmjopen.bmj.com/site/about/guidelines.xhtml

	5d	Composition, roles, and responsibilities of the coordinating centre, steering committee, endpoint adjudication committee, data management team, and other individuals or groups overseeing the trial, if applicable (see Item 21a for data monitoring committee)	31
**Introduction**			
Background and rationale	6a	Description of research question and justification for undertaking the trial, including summary of relevant studies (published and unpublished) examining benefits and harms for each intervention	4
	6b	Explanation for choice of comparators	5
Objectives	7	Specific objectives or hypotheses	6
Trial design	8	Description of trial design including type of trial (eg, parallel group, crossover, factorial, single group), allocation ratio, and framework (eg, superiority, equivalence, noninferiority, exploratory)	6
**Methods: Participants, interventions, and outcomes**			
Study setting	9	Description of study settings (eg, community clinic, academic hospital) and list of countries where data will be collected. Reference to where list of study sites can be obtained	12
Eligibility criteria	10	Inclusion and exclusion criteria for participants. If applicable, eligibility criteria for study centres and individuals who will perform the interventions (eg, surgeons, psychotherapists)	13

SAMPLE
For peer review only

Interventions	11a	Interventions for each group with sufficient detail to allow replication, including how and when they will be administered	15
	11b	Criteria for discontinuing or modifying allocated interventions for a given trial participant (eg, drug dose change in response to harms, participant request, or improving/worsening disease)	27
	11c	Strategies to improve adherence to intervention protocols, and any procedures for monitoring adherence (eg, drug tablet return, laboratory tests)	26
	11d	Relevant concomitant care and interventions that are permitted or prohibited during the trial	27
Outcomes	12	Primary, secondary, and other outcomes, including the specific measurement variable (eg, systolic blood pressure), analysis metric (eg, change from baseline, final value, time to event), method of aggregation (eg, median, proportion), and time point for each outcome. Explanation of the clinical relevance of chosen efficacy and harm outcomes is strongly recommended	22, 38
Participant timeline	13	Time schedule of enrolment, interventions (including any run-ins and washouts), assessments, and visits for participants. A schematic diagram is highly recommended (see Figure)	41

For peer review only - http://bmjopen.bmj.com/site/about/guidelines.xhtml

Sample size	14	Estimated number of participants needed to achieve study objectives and how it was determined, including clinical and statistical assumptions supporting any sample size calculations	22
Recruitment	15	Strategies for achieving adequate participant enrolment to reach target sample size	12
**Methods: Assignment of interventions (for controlled trials)**			
Allocation:			
Sequence generation	16a	Method of generating the allocation sequence (eg, computer-generated random numbers), and list of any factors for stratification. To reduce predictability of a random sequence, details of any planned restriction (eg, blocking) should be provided in a separate document that is unavailable to those who enrol participants or assign interventions	15
Allocation concealment mechanism	16b	Mechanism of implementing the allocation sequence (eg, central telephone; sequentially numbered, opaque, sealed envelopes), describing any steps to conceal the sequence until interventions are assigned	15
Implementatio n	16c	Who will generate the allocation sequence, who will enrol participants, and who will assign participants to interventions	15
Blinding (masking)	17a	Who will be blinded after assignment to interventions (eg, trial participants, care providers, outcome assessors, data analysts), and how	15

For peer review only - http://bmjopen.bmj.com/site/about/guidelines.xhtml

	17b	If blinded, circumstances under which unblinding is permissible, and procedure for revealing a participant's allocated intervention during the trial	15
**Methods: Data collection, management, and analysis**			
Data collection methods	18a	Plans for assessment and collection of outcome, baseline, and other trial data, including any related processes to promote data quality (eg, duplicate measurements, training of assessors) and a description of study instruments (eg, questionnaires, laboratory tests) along with their reliability and validity, if known. Reference to where data collection forms can be found, if not in the protocol	14
	18b	Plans to promote participant retention and complete follow-up, including list of any outcome data to be collected for participants who discontinue or deviate from intervention protocols	17
Data management	19	Plans for data entry, coding, security, and storage, including any related processes to promote data quality (eg, double data entry; range checks for data values). Reference to where details of data management procedures can be found, if not in the protocol	20
Statistical methods	20a	Statistical methods for analysing primary and secondary outcomes. Reference to where other details of the statistical analysis plan can be found, if not in the protocol	22
	20b	Methods for any additional analyses (eg, subgroup and adjusted analyses)	22

	20c	Definition of analysis population relating to protocol non-adherence (eg, as randomised analysis), and any statistical methods to handle missing data (eg, multiple imputation)	22
**Methods: Monitoring**			
Data monitoring	21a	Composition of data monitoring committee (DMC); summary of its role and reporting structure; statement of whether it is independent from the sponsor and competing interests; and reference to where further details about its charter can be found, if not in the protocol. Alternatively, an explanation of why a DMC is not needed	28
	21b	Description of any interim analyses and stopping guidelines, including who will have access to these interim results and make the final decision to terminate the trial	28
Harms	22	Plans for collecting, assessing, reporting, and managing solicited and spontaneously reported adverse events and other unintended effects of trial interventions or trial conduct	27
Auditing	23	Frequency and procedures for auditing trial conduct, if any, and whether the process will be independent from investigators and the sponsor	28
**Ethics and dissemination**			
Research ethics approval	24	Plans for seeking research ethics committee/institutional review board (REC/IRB) approval	28

Protocol amendments	25	Plans for communicating important protocol modifications (eg, changes to eligibility criteria, outcomes, analyses) to relevant parties (eg, investigators, REC/IRBs, trial participants, trial registries, journals, regulators)	28
Consent or assent	26a	Who will obtain informed consent or assent from potential trial participants or authorised surrogates, and how (see Item 32)	29
	26b	Additional consent provisions for collection and use of participant data and biological specimens in ancillary studies, if applicable	29
Confidentiality	27	How personal information about potential and enrolled participants will be collected, shared, and maintained in order to protect confidentiality before, during, and after the trial	29
Declaration of interests	28	Financial and other competing interests for principal investigators for the overall trial and each study site	32
Access to data	29	Statement of who will have access to the final trial dataset, and disclosure of contractual agreements that limit such access for investigators	21
Ancillary and post-trial care	30	Provisions, if any, for ancillary and post-trial care, and for compensation to those who suffer harm from trial participation	28

For peer review only - http://bmjopen.bmj.com/site/about/guidelines.xhtml

Dissemination policy	31a	Plans for investigators and sponsor to communicate trial results to participants, healthcare professionals, the public, and other relevant groups (eg, via publication, reporting in results databases, or other data sharing arrangements), including any publication restrictions	29
	31b	Authorship eligibility guidelines and any intended use of professional writers	Not included in this manuscript
	31c	Plans, if any, for granting public access to the full protocol, participant-level dataset, and statistical code	29
**Appendices**			
Informed consent materials	32	Model consent form and other related documentation given to participants and authorised surrogates	Available online at Open Science Framework (https://osf.io/njy2a/).
Biological specimens	33	Plans for collection, laboratory evaluation, and storage of biological specimens for genetic or molecular analysis in the current trial and for future use in ancillary studies, if applicable	23

*It is strongly recommended that this checklist be read in conjunction with the SPIRIT 2013 Explanation & Elaboration for important clarification on the items. Amendments to the protocol should be tracked and dated. The SPIRIT checklist is copyrighted by the SPIRIT Group under the Creative Commons "Attribution-NonCommercial-NoDerivs 3.0 Unported" license.

SAMPLE For peer review only

BMJ Open

Child Health, Agriculture and Integrated Nutrition (CHAIN): protocol for a randomized controlled trial of improved infant and young child feeding in rural Zimbabwe

Journal:	*BMJ Open*
Manuscript ID	bmjopen-2021-056435.R2
Article Type:	Protocol
Date Submitted by the Author:	02-Nov-2022
Complete List of Authors:	Smith, Laura ; Cornell University,<br>Chagwena, Dexter; Zvitambo Institute for Maternal and Child Health Research, Nutrition ; Ministry of Health and Child Care, Nutrition<br>Bourke, Claire ; Blizard Institute, Queen Mary University of London, UK<br>Robertson, Ruairi; Blizard Institute, Queen Mary University of London, UK,<br>Fernando, Shamiso; Zvitambo Institute for Maternal and Child Health Research, Nutrition<br>Tavengwa, Naume; Zvitambo Institute for Maternal and Child Health Research, Nutrition<br>Cairns, Jill; CIMMYT<br>Ndhlela, Thokozile; CIMMYT<br>Matumbu, Exhibit; Zvitambo Institute for Maternal and Child Health Research, Nutrition<br>Brown, Tim; Queen Mary University of London<br>Datta, Kavita; Queen Mary University of London<br>Mutasa, Batsirai; Zvitambo Institute for Maternal and Child Health Research, Nutrition<br>Tengende, Alice; Zvitambo Institute for Maternal and Child Health Research, Nutrition<br>Chidhanguro, Dzivaidzo; Zvitambo Institute for Maternal and Child Health Research, Nutrition<br>Langhaug, Lisa; Zvitambo Institute for Maternal and Child Health Research, Nutrition<br>Makanza, Maggie; Zvitambo Institute for Maternal and Child Health Research, Nutrition<br>Chasekwa, Bernard; Zvitambo Institute for Maternal and Child Health Research, Nutrition<br>Mutasa, Kuda; Zvitambo Institute for Maternal and Child Health Research, Harare, Zimbabwe<br>Swann, Jonathan; University of Southampton Faculty of Medicine<br>Kelly, Paul; Barts and The London School of Medicine<br>Ntozini, Robert; Zvitambo Institute, Biostatistics & IT<br>Prendergast, Andrew ; Queen Mary University of London,
**Primary Subject Heading**:	Nutrition and metabolism
Secondary Subject Heading:	Global health, Epidemiology, Paediatrics, Immunology (including allergy)

Keywords:	Nutrition < TROPICAL MEDICINE, Immunology < THORACIC MEDICINE, QUALITATIVE RESEARCH, Community child health < PAEDIATRICS, Nutritional support < GASTROENTEROLOGY

SCHOLARONE™ Manuscripts

Child Health, Agriculture and Integrated Nutrition (CHAIN): protocol for a randomized controlled trial of improved infant and young child feeding in rural Zimbabwe

Laura E Smith[1,2], Dexter T. Chagwena[1, 3], Claire D. Bourke[4], Ruairi C. Robertson[4], Shamiso Fernando[1], Naume V. Tavengwa[1], Jill Cairns[5], Thokozile Ndhlela[5], Exhibit Matumbu[1], Tim Brown[7], Kavita Datta[7], Batsirai Mutasa[1], Alice Tengende[1], Dzivaidzo Chidhanguro[1], Lisa Langhaug[1], Maggie Makanza[1], Bernard Chasekwa[1], Kuda Mutasa[1], Jonathan Swann[6], Paul Kelly[4], Robert Ntozini[1], Andrew J. Prendergast[1,4]

[1]Zvitambo Institute for Maternal and Child Health Research, Harare, Zimbabwe

[2]Department of Public and Ecosystem Health, Cornell University, Ithaca, NY, USA

[3]Ministry of Health and Child Care, Harare, Zimbabwe

[4]Blizard Institute, Queen Mary University of London, UK

[5]CIMMYT, Harare, Zimbabwe

[6]University of Southampton, UK

[7]School of Geography, Queen Mary University of London, UK

Short title: CHAIN Trial Protocol

Word count: 5835

Abstract: 395

Corresponding author

Dexter Chagwena, Zvitambo Institute for Maternal and Child Health Research, 16 Lauchlan Ave, Meyrick Park, Mabelreign, Harare.

Tel +263 242 306 028. Email tungadex@gmail.com or d.chagwena@zvitambo.com

ABSTRACT

For peer review only

Introduction

Over one-quarter of children in sub-Saharan Africa are stunted; however, commercial supplements only partially meet child nutrient requirements, cannot be sustainably produced, and do not resolve physiological barriers to adequate nutrition (e.g. inflammation, microbiome dysbiosis and metabolic dysfunction). Redesigning current infant and young child feeding (IYCF) interventions using locally available foods to improve intake, uptake and utilization of nutrients could ameliorate underlying pathogenic pathways and improve infant growth during the critical period of complementary feeding, to reduce the global burden of stunting.

Methods and Analysis

Child Health Agriculture Integrated Nutrition (CHAIN) is an open-label, individual household randomized trial comparing the effects of IYCF versus "IYCF-plus" on nutrient intake during infancy. The IYCF intervention comprises behaviour-change modules to promote infant nutrition delivered by community health workers, plus small-quantity lipid-based nutrient supplements (SQ-LNS) from 6-12 months of age which previously reduced stunting at 18 months of age by ~20% in rural Zimbabwe. The "IYCF-plus" intervention provides these components plus powdered NUA-45 bio-fortified sugar beans, whole egg powder, moringa leaf powder and pro-vitamin A maize. The trial will enrol 192 infants between 5-6 months of age in Shurugwi district, Zimbabwe. Research nurses will collect data plus blood, urine and stool samples at baseline (5-6 months of age) and endline (9-11 months of age). The primary outcome is energy intake, measured by multi-pass 24-hour dietary recall at 9-11 months of age. Secondary outcomes include nutrient intake, anthropometry and haemoglobin concentration. Nested laboratory sub-studies will evaluate the gut microbiome, environmental enteric dysfunction, metabolic phenotypes and innate immune function. Qualitative sub-studies will

For peer review only

For peer review only - http://bmjopen.bmj.com/site/about/guidelines.xhtml

explore the acceptability and feasibility of the IYCF-plus intervention among participants and community stakeholders, and the effects of migration on food production and consumption.

Ethics and Dissemination

This trial is registered at clinicaltrials.gov (NCT04874688) and was approved by the Medical Research Council of Zimbabwe (MRCZ/A/2679) with the final version 1.4 approved on August 20, 2021, following additional amendments. Dissemination of trial results will be conducted through the Community Engagement Advisory Board in the study district and through national-level platforms.

Strengths and Limitations of this study:

- Individually-randomised trial to demonstrate impact of a new proposed IYCF and behaviour change intervention
- Community-based study utilising local nutrient-dense foods conducted in a rural Zimbabwean community
- Improved infant and young child feeding practices promoted through a behaviour change strategy at the onset of complementary feeding period between five to six months
- Measurement of a broad range of biomarkers including dietary intake, anthropometry and laboratory assays.
- Limitation of a short follow-up period to measure outcomes.

Trial registration: NCT04874688. Registered 21 April 2021- Prospective registration, https://clinicaltrials.gov/ct2/show/NCT04874688

INTRODUCTION

Undernutrition underlies 45% of child deaths among children <5 years[1]. Linear growth failure

For peer review only - http://bmjopen.bmj.com/site/about/guidelines.xhtml

in childhood is the most prevalent form of undernutrition globally. An estimated 149 million children under 5 years of age are stunted, with a length-for-age Z-score (LAZ) more than two standard deviations below the population median[2]. Stunting affects almost one-third of children in sub-Saharan Africa, leading to reduced human capacity and increased long-term risk of chronic disease; it is therefore a surrogate marker of child health inequalities[1].

The period from 6-24 months of age is one of the most critical phases of linear growth[3], when stunting prevalence peaks due to high demand for nutrients coupled with limited quality and quantity of complementary foods[2]. Infant diets in rural sub-Saharan Africa often have low dietary diversity and a heavy reliance on white maize, which is high in starch and low in other nutrients. Interventions to improve infant and young child feeding (IYCF) typically include nutrition counselling to caregivers, plus a combination of commercial and locally available food products with or without micronutrients. However, a meta-analysis[4] of 42 studies showed only a modest impact of complementary feeding interventions on linear growth. Small-quantity lipid-based nutrient supplements (SQ-LNS), which are micronutrient-fortified ready-to-use products, show a small but measurable impact on LAZ.

We recently conducted the Sanitation Hygiene Infant Nutrition Efficacy (SHINE) trial[5], a 2×2 factorial cluster-randomized trial of improved water, sanitation and hygiene (WASH) and improved IYCF in rural Zimbabwe. A combination of IYCF messages and provision of SQ-LNS between 6-18 months of age improved LAZ of children at age 18 months by +0.16 (95%CI 0.08, 0.23) and reduced stunting by 20%[5]. The intervention also increased haemoglobin by 0.20 g/dL (95%CI 0.13, 0.28), and reduced anaemia by almost 25%[5]. However, despite this intensive IYCF intervention, 32%, 73%, and 23% of infants did not meet energy, folate and zinc/iron dietary intake requirements, respectively, as determined by 24-

For peer review only - http://bmjopen.bmj.com/site/about/guidelines.xhtml

hour recall in a subgroup at 12 months, and over one-quarter remained stunted. We also found evidence for barriers to infant nutrition, including caregiver capabilities[6], household characteristics[7], infant enteropathogen carriage[8], and systemic and intestinal inflammation[9,10], which were not resolved by the IYCF intervention[11]. Thus, we believe that persistent barriers to nutrient intake, uptake and utilization limited the impact of the IYCF intervention.

Nutrient intake is influenced by food insecurity, household purchasing power, and women's disempowerment to make decisions about land use, crop choice, and distribution of food within the household; and inequitable gender beliefs[7,12]. Nutrient uptake and utilization are influenced by intestinal pathologies which are highly prevalent among children in low-resource settings[3]. First, environmental enteric dysfunction (EED), a subclinical pathology of the small intestine characterized by intestinal inflammation and blunted villi, may impair efficient intestinal uptake of nutrients. Second, disturbance of the normal assembly of the gut microbiota may impair its roles in immune maturation, intestinal development, and nutrient metabolism, thereby impairing growth[13]. Third, systemic inflammation arising from gut pathology increases energy requirements, reduces circulating micronutrients, and inhibits the growth hormone axis[14]. Previously, barriers to intake, uptake and utilization of nutrients have largely been addressed in isolation; however, addressing these in parallel could ultimately improve growth and development in young children. Here, we present methodology for the Child Health Agriculture Integrated Nutrition (CHAIN) trial which aims to address each of these barriers together through a randomized IYCF intervention.

STUDY OVERVIEW

For peer review only - http://bmjopen.bmj.com/site/about/guidelines.xhtml

CHAIN is an open-label, individually randomized household trial comparing the effects of IYCF versus an enhanced IYCF intervention ("IYCF-plus") on energy and nutrient intake, growth, and haemoglobin in infants at high risk of stunting. The trial was completed in February 2022 while the trial design paper was under review. The overarching goal of this trial is to fill key nutrient gaps among infants in rural sub-Saharan Africa through an improved IYCF intervention using locally available foods that could ultimately be sustainable through agriculture. 192 rural Zimbabwean children were enrolled in the trial between 5-6 months of age; interventions were delivered from 6-12 months of age and the primary endline outcome of energy intake was assessed at 9 months of age (window 9-11 months). Interventions continued to be delivered until 12 months of age regardless of whether the endline visit was already completed, and all children were followed for endline visits to obtain the primary outcome. Our approach builds on the SHINE IYCF package, which reduced stunting but did not close all nutrient gaps[5]. We expect the CHAIN population will be similar to the SHINE population described above, as they are from the same rural community. CHAIN will test the impact of additional foods (powdered NUA-45 bio-fortified sugar beans, whole egg powder, moringa leaf powder and pro-vitamin A maize) that are nutrient-rich, culturally acceptable, locally sustainable and may have functional properties to ameliorate underlying pathogenic pathways, thereby tackling the identified barriers to nutrient intake, uptake and utilization. For the duration of the trial, these foods were provided by community health workers as dried powders, which can be added to infant porridge as point-of-use fortificants. However, if shown to be efficacious, this trial would provide strong proof-of-principle that a comprehensive improvement to complementary feeding using locally available foods can substantially improve child nutrition. The chosen intervention foods have potential for local communities ultimately to become self-sufficient through modifications and adaptations to local agricultural systems that include local production and processing of these foods.

For peer review only - http://bmjopen.bmj.com/site/about/guidelines.xhtml

STUDY OBJECTIVES

Objective 1. Evaluate the effect of an enhanced infant feeding intervention ("IYCF-plus") on energy intake at 9 months of age (window 9-11 months) in a randomized, community-based trial in rural Zimbabwe. *We hypothesize that provision of powdered fortificants (pro-vitamin A maize, NUA45 sugar beans, moringa and egg) for infants from 6 months of age will provide more energy at 9 months of age than the current standard-of-care IYCF intervention (trial primary outcome).*

Objective 2. Evaluate the impact of IYCF-plus on nutrient intake, growth, and haemoglobin at 9 months of age (window 9-11 months) in a randomized, community-based trial in rural Zimbabwe. *We hypothesize that IYCF-plus will improve the intake of key nutrients (protein, iron, zinc and folate) in 9-month-old infants compared to the standard-of-care IYCF intervention, and that IYCF-plus will increase length-for-age, weight-for-age, weight-for-length and haemoglobin more than IYCF (all secondary outcomes).*

Objective 3. Evaluate the impact of IYCF-plus on biological barriers to nutrient uptake and utilization at 9 months of age (window 9-11 months) in a randomized, community-based trial in rural Zimbabwe. *We hypothesize that the IYCF-plus intervention will increase microbiota maturity, ameliorate EED, reduce systemic inflammation and improve innate immune function in children aged 9 months, compared to the standard-of-care IYCF intervention.*

Objective 4. Identify metabolic signatures of the IYCF-plus intervention in young children at 9 months of age (window 9-11 months) in a randomized, community-based trial in rural Zimbabwe. *We hypothesize that the IYCF-plus intervention will increase the concentrations of*

For peer review only - http://bmjopen.bmj.com/site/about/guidelines.xhtml

essential amino acids and choline at 9 months of age more than the standard-of-care IYCF intervention.

Objective 5. Explore the acceptability and feasibility of the IYCF-plus intervention among participants and community stakeholders utilizing qualitative methodology. *Information from this assessment will be shared with policymakers to help design a larger roll-out of this intervention at district, provincial, or national level.*

Objective 6. Explore the extent to which women's empowerment influences IYCF practices and nutrition outcomes in rural smallholder agricultural households.

We hypothesize that infants of women scoring in the highest tertile of the Women's Empowerment Agriculture Index (WEAI) will have improved macro- and micronutrient intake compared to infants of women in the lowest tertile of the WEAI index.

Objective 7. Identify the extent of regional and international migration and movement (both rural-rural and rural-urban) at the household level, explore the type, frequency and impact of any associated remittance flows on food consumption and production, and consider the importance of migration to any changes in established food cultures. *Information from this assessment will be shared with policymakers to help design a larger roll-out of this intervention at district, provincial, or national level.*

RATIONALE FOR INTERVENTIONS

The CHAIN trial will compare IYCF as tested in the SHINE trial[7] versus an enhanced IYCF intervention ("IYCF-plus"). IYCF comprises a set of sequential behaviour-change modules

For peer review only - http://bmjopen.bmj.com/site/about/guidelines.xhtml

focusing on improved IYCF practices (e.g., nutrient density, feeding during illness, and dietary diversity), together with provision of daily SQ-LNS from 6-12 months of age, and powdered maize to make infant porridge. IYCF-plus comprises all the components of the IYCF intervention, plus four additional food supplements: pro-vitamin A (PVA) maize, NUA-45 sugar beans, moringa leaf powder, and whole egg powder. We have chosen this combination of 'functional' food supplements to close the remaining nutrient gaps for young children identified during SHINE (Table 1) and to ameliorate pathogenic pathways that impede uptake and utilization of nutrients.

Pro-Vitamin A (PVA) Maize is a bio-fortified maize rich in beta-carotenes, which is grown in Zimbabwe. Studies in neighbouring countries have shown that daily intake of PVA maize can improve the vitamin A status of children[15-19]. PVA maize appears less prone to contamination with aflatoxin, which is a fungal toxin affecting agricultural crops during growth, storage and processing that may impair child growth[20].

NUA45 sugar beans are a high-nutrient bean variety providing bio-fortified zinc and iron, a high protein efficiency ratio, plus folate and resistant starch. Bio-fortified beans significantly increased haemoglobin, serum ferritin, and body iron in Rwandan women[21-24].

Moringa oleifera is a widespread crop in Zimbabwe. Dried moringa leaves can be ground into a powder providing a rich source of protein, fibre, mineral and micronutrients, including vitamin A, calcium, folate, vitamin C and vitamin E, and antioxidant polyphenols. Moringa leaf powder is available in shops in Zimbabwe as a food supplement. Pilot studies show that moringa leaf powder is safe and widely accepted as a dietary supplement by children and caregivers in sub-Saharan Africa[25,26], but there have been no randomized trials.

For peer review only - http://bmjopen.bmj.com/site/about/guidelines.xhtml

Whole egg powder is commercially available, easily reconstituted and retains the nutrient content of whole eggs. Egg production is common in rural Zimbabwe. One egg per day for 6 months to children between 6-15 months of age increased LAZ by 0.63 in Ecuador[27]. This large effect is likely attributable to high-quality protein and choline, which are critical nutrients for linear growth[28 29]. Eggs contain all nine essential amino acids in proportions that closely match infant requirements for organ and muscle mass accretion. Choline is an essential nutrient that promotes growth in animal models[30]. Children with EED have reduced circulating concentrations of phosphatidylcholines[31], which are required for chondrogenesis at the growth plate, and reliant on adequate dietary intake of choline. One egg meets the majority of an infant's daily requirement. Eggs are also high in fat, energy-dense, and make modest but important contributions to vitamin A, iron, and zinc intake.

Together, these food supplements have the added plausible benefit of improving the microbiota and gut barrier function and reducing intestinal and systemic inflammation (Figure 1). The IYCF-plus intervention will increase dietary diversity and micronutrient content, which has been shown to promote healthy gut microbiota composition[32]. The resistant starch present in legumes is readily fermented by the gut microbiota to produce short-chain fatty acids, which act as a primary energy source for enterocytes, and other metabolites that maintain the integrity of the intestinal barrier[33]. Recent trials suggest that legumes modestly improve linear growth and intestinal permeability[34,35]; these effects may be enhanced through integration with other micronutrients such as vitamin A and zinc, which can improve gut barrier function[36,37]. Legume intake and high dietary quality scores have been associated with reduced systemic inflammation[38]. Finally, pre-clinical studies have reported a role for moringa in reducing oxidative stress and improving immune function[39]. We hypothesize that children receiving the

For peer review only - http://bmjopen.bmj.com/site/about/guidelines.xhtml

IYCF-plus intervention will have a more mature microbiota, reduced EED, less systemic inflammation and an associated improvement in anti-pathogen immune cell function compared to the IYCF intervention.

Ultimately, through improved agriculture and animal husbandry practices families could become self-sufficient in producing the beans, biofortified maize, moringa and eggs used for the IYCF-plus intervention for their household. As these foods are already grown locally on a small scale, there is also potential for local commercial production and processing of these products that would allow public distribution or purchasing instead of direct household-level production. Furthermore, these inputs have useful synergies: for example, moringa pods and maize bran provide food for chickens that improves their feed efficiency and increases egg production.

FORMATIVE RESEARCH AND COMMUNITY ENGAGEMENT

Formative work explored delivery and acceptability of the new food supplements proposed in CHAIN[40]. Briefly, this qualitative study purposively sampled nine community health workers (CHW) from the Shurugwi rural community where SHINE was conducted, in addition to 27 caregivers of children between 6-18 months of age. The aims of this formative work were to assess feasibility of delivering bean, egg and moringa powder to families; the acceptability of recipes devised to incorporate the new supplements into usual foods; and to inform IYCF-plus behavioural modules. The formative work also tested feasibility and uptake of this multicomponent complementary feeding and behaviour change strategy among similar rural households to the study setting.

For peer review only - http://bmjopen.bmj.com/site/about/guidelines.xhtml

Activities included focus group discussions with mothers, group-based recipe formulation, testing and review as well as home visits to assess ingredient uptake, usage, storage and recipe adherence and innovations. Household observations and views from extended family members indicated high acceptability of the new ingredients. Sensory evaluation by mothers who formulated and standardised the recipes indicated high acceptability of the complementary food recipes. All formative study participants participated in developing the behaviour-change messages and finalization of the recipes in a recipe book developed for use in the CHAIN trial IYCF-plus intervention. The formative research demonstrated feasibility of implementing this multicomponent IYCF intervention comprising behaviour change counseling to promote optimal complementary feeding practices, building self-efficacy of caregivers through cooking demonstrations at home and provision and promoting use of nutrient-dense biofortified maize, moringa, NUA45 and egg powders to feed young children. Uptake of the multicomponent IYCF intervention was high. Household observations and sensory evaluation indicated high acceptability of the new food ingredients and multicomponent IYCF intervention[40].

STUDY SETTING AND RECRUITMENT

The CHAIN trial was conducted in Shurugwi district, Zimbabwe. This is a predominantly rural, subsistence farming area, with 15% antenatal HIV prevalence and 35% stunting prevalence[7]. CHWs are a community-level cadre of healthcare workers within the Ministry of Health and Child Care. All CHWs in the study area underwent a two-week training on the interventions and have monthly supportive supervision meetings with study intervention nurses. Additionally spot checks on performance are conducted. They sensitized community stakeholders and individual families in their catchment area between birth and 5 months of age about the CHAIN study and refer those who are interested to the trial team. 282 infants turning 5 months of age from April 2021-August 2021 were identified through CHW registers. Consent

For peer review only - http://bmjopen.bmj.com/site/about/guidelines.xhtml

visits were scheduled as close as possible to children turning 5 months old, and continued until the required sample size was reached. All children who fulfilled the inclusion and exclusion criteria during enrolment were eligible for the trial, including households who had previously participated in the SHINE trial or the formative research with another child.

A research nurse visited the family's homestead to screen the child for eligibility, provide information on the trial, and undertake written informed consent with the parent/legal guardian in Shona or Ndebele. If the caregiver was not available, visits were rescheduled. All household members were encouraged to be present for consent and subsequent intervention and research visits. Interventions, delivered by CHWs, started as soon as possible after randomization, and the primary outcome is measured by research nurses at endline. Recruitment of study participants started on 26 April 2021 and study participants were followed until the end of February 2022.

For peer review only

TRIAL OUTCOMES

The primary outcome is energy intake in kcal at 9 months of age (visit window 9-11 months), as measured by multi-pass 24-hour dietary recall. Secondary and tertiary outcomes are defined in Table 2.

INCLUSION CRITERIA

Inclusion criteria were assessed by a screening questionnaire delivered to the primary caregiver by a research nurse.

- Individual level: Child age between 5-6 months
- Household level: Planning to live in the study area for the duration of the trial

EXCLUSION CRITERIA

- Severe infant disability that interferes with feeding
- Known allergy to peanuts or eggs

BASELINE DATA COLLECTION

Baseline data were collected via questionnaire on maternal, infant and household characteristics (Table 3). Maternal and infant height (ShorrBoard®), weight (Seca 874DR Mother-Baby scale), head circumference, and mid-upper arm circumference ShoreTape®) were measured. Maternal HIV status was collected by self-report and review of handheld records. Infant samples of stool, urine and blood were collected for laboratory analyses, including immunology, microbiome, and metabolic assays (full details below). Infant blood was collected by venepuncture into heparinised tubes (total 5.4 mL; maximum volume 1mL/kg), for centrifugation in the field laboratory to obtain plasma and peripheral blood cells for storage. One drop of blood was used to measure point-of-care haemoglobin, using a HemoCue 301 machine. Infant urine was collected by applying an adhesive urine bag to the infant's nappy area and waiting for the infant to pass urine during the visit. Urine was poured from the bag into a plain storage tube for transport to the field laboratory in a cool box. Infant stool was collected from the nappy into a plain tube and stored in a cool box for transport to the laboratory. If the infant did not pass stool during the visit, the mother was provided with a collection pack and instructions for how to collect the specimen the next morning, or as soon as possible thereafter, and keep the sample in a cool part of the house. The mother was asked to contact the study team once the sample has been collected and the research nurse visited the home to collect the sample. The research nurse checked the sample on arrival, labelled it with a barcode and placed it into a cool box for transport to the field laboratory. Children with

For peer review only - http://bmjopen.bmj.com/site/about/guidelines.xhtml

symptomatic mild to moderate anaemia (<11 g/dL) or with severe anaemia (<7 g/dL) were referred to local clinics. Children with moderate or severe acute malnutrition (MUAC<125mm, or weight-for-length Z-score <-2) were also referred to local clinics.

RANDOMIZATION

The randomization schema was pre-prepared by the trial statistician using the RALLOC command in STATA 14, using random permuted blocks using varying block sizes, with a 1:1 allocation to IYCF or IYCF-plus. Randomization codes are securely embedded in the trial database so that the next number is accessible to the data officer, but not the entire randomization list. Participant IDs were pre-generated and allocated to treatment arms prior to recruitment into the study. Participant IDs were assigned to a specific participant within a household after consent. Twins or eligible infants within the same household were allocated to the same trial arm. One of each twin were randomised to a specific arm. The CHW, supervised by an intervention nurse, visited the mother to tell her the trial allocation and to begin the interventions. It was not possible to blind households or fieldworkers to the interventions, but data and laboratory analysts are blinded to the allocated arm. All laboratory and data analyses will be identified by participant ID number, which does not contain details of the trial arm, and then merged by the trial statistician before reporting. Monthly reports of adverse events were reported to an independent trial safety monitor and to the Medical Research Council of Zimbabwe.

INTERVENTION DELIVERY

Behavioural modules: A total of nine interpersonal face-to-face counseling modules were delivered to caregivers in each arm by CHWs during 10 home visits, which coincide with key infant ages, so that sequential age-appropriate messages about complementary feeding are introduced and reinforced (Table 4). The CHW introduces the food supplements in both arms,

For peer review only - http://bmjopen.bmj.com/site/about/guidelines.xhtml

demonstrates how to add them to food, monitors for any adverse reactions and provides monthly re-supplies. Modules are interactive and are delivered to all household members present. The last module was delivered at 11 months of age, with infant food supplements provided until 12 months of age, when all trial interventions end. Using this design, we ensured that all infants are still receiving the IYCF or IYCF-plus interventions when endline data collection occurred at 9 months (window 9-11 months) of age.

If a module was missed, the CHW attempted to catch up by scheduling a new date, or else summarized the missed module at the next scheduled visit. If a caregiver moved within the study area, the CHW covering that area delivered modules to the caregiver, where possible; if the caregiver moved out of the study area, she did not receive study modules or food supplements. Endline data collection visits are conducted regardless of where the caregiver moves to. The estimated length of time exposed to the intervention is between eight and eleven months, from five to fifteen months. Median estimated length of time exposed to the intervention is eight months.

IYCF arm: Core IYCF counselling modules were complemented by provision of one sachet of 20g SQ-LNS daily between 6-12 months of age. SQ-LNS is a peanut-based supplement rich in calories, protein and micronutrients, which can be consumed directly from the sachet or mixed with porridge. Families also received a daily infant ration of white maize to feed the baby as porridge.

IYCF-plus arm: As in the IYCF arm, core counselling modules focusing on complementary feeding were delivered by the CHW, with provision of one sachet per day of 20g SQ-LNS between 6-12 months of infant age. In addition, families received NUA-45 biofortified bean

For peer review only - http://bmjopen.bmj.com/site/about/guidelines.xhtml

powder, whole egg powder and moringa leaf powder for provision to the study child. The quantity of food supplements provided was based on the child's age to ensure the daily recommended nutrient intake was met if the food supplement was consumed (Table 5). Families also received a daily infant ration of PVA biofortified maize to feed the baby as porridge. Supplements were delivered in sealed containers, which the mother is asked to keep in a cool part of the house. Six recipes promoting high-quality staple foods, which were developed and standardized in the formative studies[40], are outlined in a recipe book, with cooking demonstrations given by the CHW.

INTERVENTION DELIVERY AND UPTAKE

Each CHW delivered the intervention to 1-3 enrolled households during the study. Eight intervention nurses (separate from the research nurses) were responsible for monitoring delivery of modules and food supplements and evaluating intervention uptake and compliance to recommended behaviours by caregivers. Intervention nurses did not provide counseling to mothers but did provide supportive supervision to CHWs by scheduled attendance at some household visits to provide feedback and by conducting unscheduled spot checks. Intervention nurses attended visits each time a CHW was delivering a module for the first time and additionally if needed. Intervention nurses held monthly meetings with the CHWs they supervise, to share learning, capture data on module delivery, and provide re-training as needed. A module delivery and intervention uptake checklist was completed by CHWs at each module delivery visit, and submitted to intervention nurses during monthly meetings. The checklist recorded modules that have been successfully delivered and dates of delivery. Data on uptake of interventions and compliance to recommended behaviours will include utilization of food supplements, any sharing of food supplements observed and involvement of other family members in child feeding assessed by a caregiver questionnaire.

For peer review only - http://bmjopen.bmj.com/site/about/guidelines.xhtml

FOLLOW-UP DATA COLLECTION

Households were visited by a research nurse at 9 months of infant age (window 9-11 months) for endline data collection. If the child was not present, the visit was rescheduled. Infant weight, length, MUAC, and head circumference were measured, and samples of blood, urine and stool collected, using the same methods as at baseline. Children with illness were referred to local clinics, using the same criteria as at baseline.

The trial primary outcome was measured by research nurses via 24-hour multi-pass dietary recall. A subgroup of 50% of randomly selected household had a second 24-hour dietary recall visit approximately one week later. This method provides a robust and validated measure of nutrient intake based on a comprehensive and standardized assessment[41]. The dietary recall method assesses all food and beverages consumed in the previous 24 hours (including supplements provided by the trial) and comprises five passes. In the first pass, the research nurse asks the caregiver to list all foods consumed by the child during the last day, and to list any night feeds. In the second pass, the caregiver is asked to list all activities they undertook, and whether they fed the child food between activities; this helps the caregiver remember all feeding episodes. In the third pass, more details about foods and beverages are collected, including the time and place of preparation, ingredients, and brand of foods given. In the fourth pass, the caregiver estimates the portion size fed to the child. Research nurses carry samples of the most consumed foods and ask the caregiver to estimate the amount fed to the child. The fieldworker then transfers the estimated portion to a standard cup, spoon or digital scale for recording. In the final pass, the caregiver recalls if there were any foods or meals that have not already been mentioned. Caregivers are also asked about the general health of the child on the

For peer review only

previous day, whether the child's intake was less or more than usual, and how many times the child was breastfed.

Data from the 24-hour recall will be converted to observed energy and nutrient intakes by the following steps:

1. Ingredients and portion sizes were measured and weighed in grams where possible. For ingredients that could not be weighed, they will be converted to grams using locally collected data on food densities, supplemented with food density data from the FAO, USDA and NDSR [42-44].

2. Mixed dishes were disaggregated into ingredients and entered into Nutrisurvey to calculate nutrients in 100 grams of food.

3. Energy from each individual food/ingredient will be estimated using food composition data from regional food databases and USDA databases that have been collated for use in Zimbabwe over several studies[45].

Estimated energy and nutrient intakes will be compared with WHO-estimated energy requirements, the Institute of Medicine, Food and Nutrition Board recommended daily allowances for protein and choline, and the WHO-recommended nutrient intakes (RNIs) for other vitamins and minerals. For breastfeeding children, we will calculate the required nutrient intake from complementary foods by subtracting the amount of each nutrient in 550 g breast milk from the total requirement which is the estimated intake of breast milk for 9-11 month old children [46]. Energy requirements are calculated as kilojoules required per kilogram of body weight for breastfed children[47]; however, we will apply the slightly higher energy requirement estimated by Butte for children in low-income settings, which reflect increased needs due to

For peer review only - http://bmjopen.bmj.com/site/about/guidelines.xhtml

greater infection burden[48]. Protein requirements will be defined as the WHO-recommended safe level of protein intake for 9-11 month old children[49]. Fat requirement will be defined as 35% energy requirement, which is the mid-point of several recommendations[50]. Micronutrient requirements will be defined as WHO-recommended nutrient intakes, except for calcium which is defined as the mean of the WHO RNI and US RNI. For zinc and iron, we will assume 30% and 10% bioavailability, respectively[30,47,51,52]. For breastfed children, we will estimate the required nutrient intake from complementary foods by subtracting the amount of each nutrient in breast milk from the total requirement[52]. Using this comprehensive approach, we will determine the impact of the IYCF-plus versus IYCF intervention on energy intake (primary outcome) and the relative contributions of supplements (including SQ-LNS) and other complementary foods in closing infant nutrient gaps across trial arms. In addition to assessing total protein intake, we will explore essential amino acid intake, digestibility-adjusted protein intake and inflammation-adjusted protein intake[53].

TRAINING OF COMMUNITY HEALTH WORKERS, INTERVENTION AND RESEARCH NURSES

Eight intervention nurses (INs) underwent a two-week training on delivery of the trial interventions and provide supportive supervision to CHWs. All CHWs in the study area underwent a two-week training on the interventions. A training cascading approach was utilized where intervention nurses took part in training of CHWs, supporting other research staff. Monthly supportive-supervision cluster meetings with CHWs were conducted by INs. Four research nurses (Data Collectors- DCs) underwent a four-week training on conducting consenting, baseline, endline and 24-hour dietary recall interviews. Research nurses were trained separately from INs and all activities they carried out were conducted separately to avoid research bias.

For peer review only - http://bmjopen.bmj.com/site/about/guidelines.xhtml

DATA COLLECTION AND MANAGEMENT

Research data were collected onto electronic case report forms (eCRFs) using pre-programmed tablets, with Open Data Kit (ODK) software. Full data validation procedures were programmed into the tablets including embedded skip patterns, data completeness and plausibility checks. All data were checked daily by the field data officer, with implausible values verified or recollected. Back-up paper CRFs are carried by research nurses in the event of tablet failure. Data are uploaded from tablets onto a secure trial database daily and backed up onto a secure cloud database hosted on Microsoft Azure. Data will be stored for 20 years.

Each participant is allocated a participant identifier which is used on all forms to identify the child. Personal information and data are kept confidential and managed in accordance with the requirements of the Medical Research Council of Zimbabwe. Paper records (e.g., CRF, clinical / laboratory information and test results) will be entered into the electronic database; source documents will be stored in a secure, locked cupboard at each study site, and kept fully confidential. Data will be kept securely on a password-protected customised MS-SQL Server trial database and hosted by Microsoft Azure.

SAMPLE MANAGEMENT

Preprinted barcodes identifying the participant ID and sample type were adhered to collection tubes in the field, which were transported to the laboratory at room temperature (for blood) or in a cooler bag (for stool and urine samples). When samples arrived at the laboratory, they were processed and aliquoted into cryovials which were labeled with barcodes identifying the

For peer review only

participant ID, sample type and aliquot number. Samples were stored in the field laboratory at -80° Celsius. At regular intervals, samples were transported to the main laboratory in Harare, where they were stored at -80° Celsius until analysis or shipment. All samples will be shipped to external laboratories on dry ice. Sample lists will be maintained in the main trial database. If participants consented to long-term storage, samples will be stored for up to 20 years.

SAMPLE SIZE

The sample size of 192 infants assumes 10% loss to follow-up due to withdrawal and infant deaths, meaning there will be an estimated 86 evaluable infants per group at endline. This sample size provides 86% power at 5% significance to detect a 20% increase in the proportion of infants achieving their recommended energy intake (by 24-hour dietary recall) in the IYCF-plus arm, assuming that only 65% of infants are meeting requirements in the IYCF (standard-of-care) arm based on SHINE data. If loss-to-follow is as high as 15%, we will still have 80% power to detect a 20% increase in the proportion reaching their daily energy intake (study primary outcome).

STATISTICAL ANALYSIS

Analysis of trial outcomes will be by intention-to-treat. P values will be 2-sided and interpreted as significant if $p<0.05$. Binary outcomes will be compared between groups using the Chi-square test and logistic regression to compute odds ratios and corresponding 95% confidence intervals. Other categorical outcomes with more than two levels will be compared between groups using Chi-squared tests and multinomial regression. Continuous outcomes will be compared using simple t-tests and linear regression. Non-normal continuous outcomes will be transformed appropriately before analysis. Robust standard error estimates will be used to estimate confidence intervals.

For peer review only - http://bmjopen.bmj.com/site/about/guidelines.xhtml

The primary outcome, percentage of infants meeting energy intake (kcal/day), will be calculated using measured intake data from dietary recalls from all participants. Dietary energy intake (DEI) was collected through an interactive, multiple-pass, 24-hour recall at the 9-month endpoint among all children, and in 50% of children through an additional dietary recall seven days later. The dietary energy intake estimate will be used with a logistic regression model clustering for the repeat measure using the multilevel mixed-effects logistic regression method. This method will be implemented within STATA. It is well-recognized the dietary intake data contains a lot of uncertainty and thus we will conduct a sensitivity analysis using the NCI method calculated for usual overall average intake. We will estimate the mean and percentiles of usual energy intake distributions using the National Cancer Institute (NCI) method [54,55]. This method adjusts for measurement error – primarily due to day-to-day variability in intakes – in observed, single-day estimates of nutrient intakes. We will use the NCI macros, DISTRIB and MIXTRAN, plus bootstrapped standard errors for hypothesis testing [25, 27]. The MIXTRAN macro fits a mixed effects model of usual energy intake, and the DISTRIB macro uses a Monte Carlo procedure to estimate percentiles of the usual intake distribution. We will use the MIXTRAN macro to fit a model of energy intake with a fixed effect for intervention group and the DISTRIB macro to estimate usual energy intake distributions by treatment group. We will bootstrap the parameters to estimate the standard error of the mean usual intakes. We will use the point estimates and standard error estimates to construct 95% confidence intervals and calculate p-values for difference in mean by intervention group based on Welch's t-test. Bootstrapped standard errors will be calculated using bootstrap samples. When intake or the number of repeat recalls is low, it is common for the NCI method to fail to converge. In cases where

For peer review only - http://bmjopen.bmj.com/site/about/guidelines.xhtml

convergence prohibits bootstrapping we will test hypotheses using the Wilcoxon testing for clustered method.

We will also interpret the mean intake of each nutrient for the primary and secondary analysis in each arm and will present mean differences with the 95% confidence interval for interpretation.

All analyses will be pre-specified in a Statistical Analysis Plan and posted online at Open Science Framework before analyses begin (https://osf.io/njy2a/). A per protocol analysis will be conducted using adherence data to pre-define the per protocol population. Adherence will be assessed by self-report in a 7-day recall. We will undertake two subgroup analyses: i) by infant sex; and ii) by maternal HIV status, if we find some evidence of interactions with the intervention ($p<0.10$).

LABORATORY ANALYSES

Biological samples will be used to evaluate the nutrient profiles, EED, systemic inflammation, innate immune function, metabolic phenotype and the gut microbiota, as shown in Table 6. This is a tertiary outcome being measured on everyone with samples available for baseline and endline. The study is not powered for this outcome and is exploratory.

EED and systemic inflammation: We will use a combination of plasma and stool ELISA assays to compare the impact of IYCF and IYCF-plus on EED, by characterising the hypothesised causal pathway from the gut to growth, measuring markers of intestinal inflammation (stool myeloperoxidase, neopterin) small intestinal damage (plasma I-FABP), intestinal permeability (alpha-1 antitrypsin), microbial translocation from the gut (plasma LBP, sCD14), systemic

For peer review only - http://bmjopen.bmj.com/site/about/guidelines.xhtml

inflammation (AGP, CRP, TNFα) and growth hormone activity (plasma IGF-1). We will also measure aflatoxin M1 in urine, to assess recent exposure to dietary aflatoxin, which is a plausible cause of EED and may be reduced in the IYCF-plus arm since PVA maize appears less prone to fungal contamination.

Immune function: Immune cell activation is metabolically costly and chronic activation by recurrent infections/EED may create a barrier to children meeting their nutrient requirements by: i) driving inflammation and oxidative stress, ii) contributing to enteropathy, iii) compromising the capacity of innate immune cells to defend against new infections, which further deplete dietary nutrients. Multiple innate and adaptive immune mediators are dysregulated in undernourished children[56], but little is known about if/how nutritional interventions affect immune defences[57]. We will use whole blood samples to compare innate immune cell phenotype and function between randomised groups. We will quantify surface expression of activation markers HLA-DR, CD64 and CD16 on blood monocytes, and CD64 and CD62L on blood monocytes and neutrophils via flow cytometry. To characterise the functional capacity of innate immune cells to respond to pathogen challenge we will quantify pro-inflammatory cytokine secretion in supernatants derived from whole blood cultures with and without bacterial lipopolysaccharide (LPS). Whole blood culture with and without fluorescent-labelled *Escherichia coli*-coated bioparticles will be used to quantify uptake of bacteria via flow cytometry.

Metabolic phenotyping: A targeted ultra-performance liquid chromatography-mass spectrometry (UPLC-MS)-based assay will be used to measure tryptophan-related metabolites in plasma (funding permitting)[58]. This includes metabolites involved in the kynurenine, serotonin, and indole pathways. In addition, downstream NAD+ related metabolites, such as

For peer review only - http://bmjopen.bmj.com/site/about/guidelines.xhtml

nicotinic acid, nicotinamide, and nicotinamide-riboside will be measured together with markers of systemic inflammation (neopterin), enterocyte mass (citrulline) and the neurotransmitter dopamine. ^{1}H nuclear magnetic resonance (NMR) spectroscopy will be used to characterize the metabolic profiles of urine, plasma and faecal water samples. This approach measures H-containing metabolites present above the limit of detection in the samples in an untargeted manner. This captures information on amino acids, gut microbial metabolites, and metabolites involved in choline and energy metabolism. It may also be used to study dietary components and assess variation in their digestion.

Microbiome sequencing: Whole metagenome shotgun sequencing will be employed on stool samples to examine the effect of the trial interventions on the gut microbiome and its association with growth. DNA will be extracted from stool aliquots (200mg) using the Qiagen PowerFecal Pro DNA kit, followed by metagenomic sequencing library preparation. Following qualitative and quantitative assessment of sequencing libraries, sequencing will be performed via the HiSeq 2500 platform producing 6-10 million sequencing reads per sample. Following quality control and trimming of human reads, sequencing reads will be processed through validated pipelines to generate compositional (MetaPhlAn v.3) and functional (HUMAnN v.3) readouts of the gut microbiome. Microbiome maturity will be assessed as previously described using a control dataset generated from the SHINE trial[59]. Aliquots of stool stored in glycerol will be used to isolate microorganisms of interest for downstream experiments assessing the influence of the gut microbiome on EED and growth.

QUALITATIVE STUDIES

The first qualitative substudy will develop a more in-depth understanding of how the CHAIN interventions were integrated into household nutrition practices, and the cultural, economic,

For peer review only

and social processes that shape this. Up to 20 families were purposively sampled based on type of household, caregiver characteristics and trial arm. Semi-structured interviews were conducted to explore a range of themes, including any changes in the participants' receptiveness to the food supplements or children's response to them, the ability to maintain compliance with the food preparation guidelines, and challenges they may have experienced (e.g., accessing or storing the food supplements). The interviews also focus on relevant cultural, economic, and social processes operating at the household level that may influence food preparation and consumption practices and explore how these shape the sustainability of the intervention. Additionally, emphasis was placed on exploring the role that household and area-level gender dynamics may play in decision-making practices.

A second qualitative substudy will identify the ways in which household migration (defined here as people who have moved away from a household for 3 months or more) influences food security. 30 participating households were identified from the baseline survey as having at least one family member who has migrated across a range of geographical scales (local, regional, national, and international) and where possible representing different household types (including, male- or female-headed households, orphan-headed households, elder-headed households). In-depth interviews were conducted with study participants to explore the interactions that exist between household migration and remittance practices (receiving and sending) and their potential to influence household food consumption and production practices. In addition, the in-depth interviews aim to consider the importance of geographical scale to remitting practices and their influence on food consumption and production and investigate possible interactions between migration and household participation in the CHAIN intervention.

For peer review only - http://bmjopen.bmj.com/site/about/guidelines.xhtml

Interviews and focus groups were audio recorded, for subsequent transcription and translation; transcripts will be entered into NVivo for coding, analysis, and interpretation. Analysis will proceed using both deductive and inductive approaches and will utilise the framework method often employed in multidisciplinary health-related research. All audio recordings will be destroyed, although transcriptions will be stored securely and made available for future analysis as required.

TRIAL RISKS AND ADVERSE EVENTS

All interventions are commercially available foods (beans, egg powder, moringa, maize) or food supplements that are widely used globally (SQ-LNS). Peanuts and eggs are both staple foods in rural Zimbabwe; although both are potentially allergenic among infants in high-income settings, the prevalence of food allergies in sub-Saharan Africa is extremely low[5,60,61]. SQ-LNS, which contains peanuts, was used in the same community in the SHINE trial among more than 2000 infants. From over 365,000 doses of SQ-LNS that were given to infants in SHINE, there were no allergic reactions, and no serious adverse events[5]. Only two adverse events were possibly or probably related to SQ-LNS; both resolved without sequelae[5]. Children with a known allergy to peanuts or eggs will be excluded from the study to further minimise the risk of adverse events. All adverse events will be reported and reviewed by the study physician, and a tabulated monthly summary will be sent to an independent safety monitor. All serious adverse events and trial-related adverse events will be reported to the Medical Research Council of Zimbabwe according to established timeframes.

The COVID-19 pandemic poses a potential risk to research activities in the community**.** We ensured that research staff wear personal protective equipment and practice physical distancing to keep themselves and research participants safe. All staff were trained in COVID protocols.

For peer review only - http://bmjopen.bmj.com/site/about/guidelines.xhtml

Interviews were conducted in a confidential outdoor part of the homestead wherever possible. CHWs conduct their activities with guidance for safe working from the Ministry of Health and Child Care. Procedures were reviewed as the pandemic progresses, and any changes discussed with the District Health Executive and with the Medical Research Council of Zimbabwe. The food supplements are stopped at 12-15 months and not continued after the trial.

ETHICS

Ethical approval was granted by the Medical Research Council of Zimbabwe (MRCZ/A/2679), and Sponsorship provided by Queen Mary University of London (Joint Management Research Office, http://www.jrmo.org.uk/). All caregivers/legal guardians provided written informed consent on behalf of their child. Information and consent procedures were administered in each individual's language of choice (Shona, Ndebele and/or English). Because mothers often need to consult with other family members before deciding about their child's participation in a trial, we include other family members in consent discussions if the mother wishes. If the caregiver wanted to think about participation or consult, the consenting visit was rescheduled to give her time. Illiterate mothers who understand a verbal explanation of the study can provide a thumb imprint on the consent form in the presence of an independent witness. Mothers aged between 15-18 years are considered emancipated minors under Zimbabwean law and could consent on behalf of their child. Families were provided with a small gift (soap and Vaseline) for each research data collection visit. Consent forms are available online (https://osf.io/njy2a/).

Since the IYCF intervention in the SHINE trial led to 20% reduction in stunting[5], we are providing IYCF as the enhanced standard-of-care for the control arm, since we believe it is unethical not to provide this intervention in the exact same population. Provision of food supplements may confer benefits for infant nutrition and growth, and for household wellbeing,

For peer review only - http://bmjopen.bmj.com/site/about/guidelines.xhtml

but we believe it is ethically justifiable to randomize the supplements of powdered egg, NUA45 sugar beans, moringa and PVA maize, since none are routinely provided to households in the community and there is scientific equipoise as to whether these food supplements will bring additional benefit to what is already being provided in the IYCF arm.

DISSEMINATION

Trial results will be presented at international conferences and published in open-access journals. Data will be available on request, after publication of the primary trial findings, by contacting the Trial Management Group, with details of data access requests available on the Zvitambo website. Results will be presented to the Ministry of Health and Child Care in Zimbabwe and will be disseminated to the study district through the Community Engagement Advisory Board which comprises peers selected by the community to review ongoing research studies in the community. Results will also be presented to UNICEF to inform the design and scale-up of IYCF programmes in Zimbabwe.

PATIENT AND PUBLIC INVOLVEMENT

Research questions and outcome measures were informed from gaps identified in the SHINE trial, incorporating the priorities, experiences, and preferences of community members. Community members who participated in the SHINE study, living in the same setting the CHAIN trial was conducted informed CHAIN's research questions, design and new test foods used based on their preferences and priorities. Families who participated in the formative study informed the trial design and the burden of the intervention. Trial findings will be disseminated to participants through community engagement meetings coordinated by the Shurugwi Community Engagement Advisory Board

For peer review only - http://bmjopen.bmj.com/site/about/guidelines.xhtml

DISCUSSION

Stunting remains a global health challenge which hinders human capital and perpetuates poverty. There is an urgent need for more efficacious nutrition-specific interventions to enhance child linear growth during complementary feeding. Currently, multiple barriers constrain nutrient intake, uptake and utilization, including marginal diets, EED, perturbations of the microbiome, metabolic dysregulation and chronic inflammation. Current IYCF approaches partly close nutrient gaps but require optimisation to fully restore healthy child growth. Utilising locally available foods with functional properties to supplement current IYCF approaches could have the dual goal of closing nutrient gaps in infancy and ameliorating pathogenic barriers to nutrient uptake and utilisation. We will test these ideas using a rigorous proof-of-concept trial design in a rural, subsistence farming community with a high burden of stunting, and evaluate acceptability, feasibility and sustainability of the approach for longer-term and larger-scale deployment. Identifying new, sustainable ways to improve dietary quality and reduce stunting would help to accelerate progress towards 2030 global targets, and could have major benefits for long-term health, development and human capital.

DECLARATIONS

Ethics approval and consent to participate: Ethical permission has been granted by the Medical Research Council of Zimbabwe (MRCZ/A/2679). All mothers/caregivers will provide written informed consent on behalf of their child to participate.

Authors' contributions: Trial design: LES, CDB, RCR, NVT, DTC, JC, TN, TB, KD, KM, JS, PK, RN, AJP. Secured funding: LES, CDB, RCR, NVT, JC, TN, TB, KD, KM, JS, PK, RN, AJP. Agriculture design and expertise: JC, TN. Laboratory design and methods

For peer review only

development: CDB, RCR, KM, JS, PK, AJP. Data management and analysis plan: BM, BC, RN. Formative work: LES, DTC, SF, NVT, LFL, TB, KD, BM, DC, AJP. Qualitative design and expertise: LES, TB, KD, DTC, SF, EM, NVT, LFL, MM. Training and implementation: AT, BM, DTC, KM. Study oversight: LES, JC, LFL, DTC, RN, AJP. All authors read and approved the final manuscript.

Acknowledgements: We appreciate the families in the formative study and the Shurugwi Community Engagement Advisory Board who helped in the design and conduct of the trial.

Funding: UK Research and Innovation Biotechnology and Biological Sciences Research Council (UKRI-BBSRC), from the Global Challenges Research Fund (GCRF) under Food & Nutrition Research for Health in the Developing World: Bioavailability and Nutrient Content. RCR and AJP are funded by Wellcome (grants 206455/Z/17/Z and 108065/Z/15/Z). CDB is funded by a joint award from Wellcome and the Royal Society (206225/Z/17/Z). The second qualitative sub-study is funded by the Arts and Humanities Research Council (AHRC), UK (AH/T004428/1). Funding bodies had no role in the study design, implementation, analysis and interpretation of the data. The principal investigators had no financial and other competing interests.

Competing interests: The authors declare that they have no competing interests.

For peer review only - http://bmjopen.bmj.com/site/about/guidelines.xhtml

REFERENCES

1. Black RE, Victora CG, Walker SP, et al. Maternal and child undernutrition and overweight in low-income and middle-income countries. *Lancet*. Aug 3 2013;382(9890):427-451. doi:10.1016/S0140-6736(13)60937-X

2. UNICEF: Levels and trends in child malnutrition, United Nations Children's fund (UNICEF). World Health Organization, International Bank for Reconstruction and Development/The World Bank Levels and trends in child malnutrition: key findings of the 2019 edition of the joint child malnutrition estimates Geneva: World Health Organization 2019.

3. Prendergast AJ, Humphrey JH. The stunting syndrome in developing countries. *Paediatr Int Child Health*. Nov 2014;34(4):250-65. doi:10.1179/2046905514Y.0000000158

4. Dewey KG, Adu-Afarwuah S. Systematic review of the efficacy and effectiveness of complementary feeding interventions in developing countries. *Maternal & child nutrition*. Apr 2008;4 Suppl 1:24-85. doi:10.1111/j.1740-8709.2007.00124.x

5. Humphrey JH, Mbuya MNN, Ntozini R, et al. Independent and combined effects of improved water, sanitation, and hygiene, and improved complementary feeding, on child stunting and anaemia in rural Zimbabwe: a cluster-randomised trial. *Lancet Glob Health*. Jan 2019;7(1):e132-e147. doi:10.1016/S2214-109X(18)30374-7

6. Matare CR, Mbuya MN, Dickin KL, et al. Maternal Capabilities are associated with child caregiving behaviors among women in rural Zimbabwe. *The Journal of Nutrition*. 2020;

7. Humphrey J, Mbuya M, Ntozini R. Sanitation Hygiene Infant Nutrition Efficacy (SHINE) Trial Team. Independent and combined effects of improved water, sanitation, and hygiene, and improved complementary feeding, on child stunting and anaemia in rural Zimbabwe: a cluster-randomised trial. *Lancet Glob Health*. 2019;7:e132-47.

8. Rogawski McQuade ET, Platts-Mills JA, Gratz J, et al. Impact of water quality, sanitation, handwashing, and nutritional interventions on enteric infections in rural Zimbabwe: the Sanitation Hygiene Infant Nutrition Efficacy (SHINE) Trial. *The Journal of infectious diseases*. 2020;221(8):1379-1386.

9. Gough EK, Moulton LH, Mutasa K, et al. Effects of improved water, sanitation, and hygiene and improved complementary feeding on environmental enteric dysfunction in children in rural Zimbabwe: A cluster-randomized controlled trial. *PLoS Negl Trop Dis*. Feb 2020;14(2):e0007963. doi:10.1371/journal.pntd.0007963

10. Mutasa K, Ntozini R, Mbuya MNN, et al. Biomarkers of environmental enteric dysfunction are not consistently associated with linear growth velocity in rural Zimbabwean infants. *Am J Clin Nutr*. Mar 19 2021;doi:10.1093/ajcn/nqaa416

11. Gough EK, Moulton LH, Mutasa K, et al. Effects of improved water, sanitation, and hygiene and improved complementary feeding on environmental enteric dysfunction in children in rural Zimbabwe: A cluster-randomized controlled trial. *PLoS neglected tropical diseases*. 2020;14(2):e0007963.

12. Desai A, Smith LE, Mbuya MN, et al. The SHINE trial infant feeding intervention: pilot study of effects on maternal learning and infant diet quality in rural Zimbabwe. *Clinical Infectious Diseases*. 2015;61(suppl_7):S710-S715.

13. Robertson RC, Manges AR, Finlay BB, Prendergast AJ. The human microbiome and child growth–first 1000 days and beyond. *Trends in microbiology*. 2019;27(2):131-147.

14. Prendergast AJ, Rukobo S, Chasekwa B, et al. Stunting is characterized by chronic inflammation in Zimbabwean infants. *PLoS One*. 2014;9(2):e86928. doi:10.1371/journal.pone.0086928

15. Palmer AC, Craft NE, Schulze KJ, et al. Impact of biofortified maize consumption on serum carotenoid concentrations in Zambian children. *Eur J Clin Nutr*. Feb 2018;72(2):301-303. doi:10.1038/s41430-017-0054-1

For peer review only - http://bmjopen.bmj.com/site/about/guidelines.xhtml

16. Palmer AC, Siamusantu W, Chileshe J, et al. Provitamin A–biofortified maize increases serum β-carotene, but not retinol, in marginally nourished children: a cluster-randomized trial in rural Zambia. *The American journal of clinical nutrition*. 2016;104(1):181-190. doi:10.3945/ajcn.116.132571
17. Palmer AC, Jobarteh ML, Chipili M, et al. Biofortified and fortified maize consumption reduces prevalence of low milk retinol, but does not increase vitamin A stores of breastfeeding Zambian infants with adequate reserves: a randomized controlled trial. *The American journal of clinical nutrition*. May 8 2021;113(5):1209-1220. doi:10.1093/ajcn/nqaa429
18. De Moura FF, Palmer AC, Finkelstein JL, et al. Are Biofortified Staple Food Crops Improving Vitamin A and Iron Status in Women and Children? New Evidence from Efficacy Trials. *Advances in nutrition*. 2014;5(5):568-570. doi:10.3945/an.114.006627
19. M Haskell, S.A. Tanumihardjo, A Palmer, A Melse-Boonstra, E Talsma, B Burri. Efficacy of Iron-Biofortified Crops. *African Journal of Food Agriculture Nutrition and Development*. 2017;17(2)doi:10.4314/ajfand.v17i2
20. Smith L, Stoltzfus R, Prendergast A. Food-Chain Mycotoxin Exposure, Gut Health and Impaired Growth: A conceptual framework *Advances in Nutrition*. 2012;3:1-6.
21. Haas JD, Luna SV, Lung'aho MG, et al. Consuming Iron Biofortified Beans Increases Iron Status in Rwandan Women after 128 Days in a Randomized Controlled Feeding Trial. *J Nutr*. Aug 2016;146(8):1586-92. doi:10.3945/jn.115.224741
22. Gomes M.J.C., Martino H.S.D., Tako E. Zinc-biofortified staple food crops to improve zinc status in humans: a systematic review. . *Critical Reviews in Food Science and Nutrition 1-13*. 2021;doi:10.1080/10408398.2021.2010032
23. Finkelstein JL, Haas JD, Mehta S. Iron-biofortified staple food crops for improving iron status: a review of the current evidence. *Current Opinion in Biotechnology*. 2017/04/01/ 2017;44:138-145. doi:https://doi.org/10.1016/j.copbio.2017.01.003
24. Finkelstein JL, Fothergill A, Hackl LS, Haas JD, Mehta S. Iron biofortification interventions to improve iron status and functional outcomes. *Proceedings of the Nutrition Society*. 2019;78(2):197-207. doi:10.1017/S0029665118002847
25. Barichella M, Pezzoli G, Faierman SA, et al. Nutritional characterisation of Zambian Moringa oleifera: acceptability and safety of short-term daily supplementation in a group of malnourished girls. *Int J Food Sci Nutr*. Feb 2019;70(1):107-115. doi:10.1080/09637486.2018.1475550
26. Boateng L, Nyarko R, Asante M, Steiner-Asiedu M. Acceptability of Complementary Foods That Incorporate Moringa oleifera Leaf Powder Among Infants and Their Caregivers. *Food and nutrition bulletin*. Mar 2018;39(1):137-148. doi:10.1177/0379572117708656
27. Iannotti LL, Lutter CK, Stewart CP, et al. Eggs in Early Complementary Feeding and Child Growth: A Randomized Controlled Trial. *Pediatrics*. Jul 2017;140(1)doi:10.1542/peds.2016-3459
28. Semba RD, Shardell M, Sakr Ashour FA, et al. Child Stunting is Associated with Low Circulating Essential Amino Acids. *EBioMedicine*. Apr 2016;6:246-252. doi:10.1016/j.ebiom.2016.02.030
29. Ghosh S, Suri D, Uauy R. Assessment of protein adequacy in developing countries: quality matters. *Br J Nutr*. Aug 2012;108 Suppl 2:S77-87. doi:10.1017/S0007114512002577
30. Zeisel SH, Blusztajn JK. Choline and human nutrition. *Annu Rev Nutr*. 1994;14:269-96. doi:10.1146/annurev.nu.14.070194.001413
31. Baron J, Savendahl L, De Luca F, et al. Short and tall stature: a new paradigm emerges. *Nat Rev Endocrinol*. Dec 2015;11(12):735-46. doi:10.1038/nrendo.2015.165
32. Heiman ML, Greenway FL. A healthy gastrointestinal microbiome is dependent on dietary diversity. *Mol Metab*. May 2016;5(5):317-320. doi:10.1016/j.molmet.2016.02.005

For peer review only - http://bmjopen.bmj.com/site/about/guidelines.xhtml

33. Kelly CJ, Zheng L, Campbell EL, et al. Crosstalk between Microbiota-Derived Short-Chain Fatty Acids and Intestinal Epithelial HIF Augments Tissue Barrier Function. May 13 2015;17(5):662-71. doi:10.1016/j.chom.2015.03.005

34. Agapova SE, Stephenson KB, Divala O, et al. Additional Common Bean in the Diet of Malawian Children Does Not Affect Linear Growth, but Reduces Intestinal Permeability. *J Nutr*. Feb 1 2018;148(2):267-274. doi:10.1093/jn/nxx013

35. Stephenson KB, Agapova SE, Divala O, et al. Complementary feeding with cowpea reduces growth faltering in rural Malawian infants: a blind, randomized controlled clinical trial. *The American journal of clinical nutrition*. Dec 2017;106(6):1500-1507. doi:10.3945/ajcn.117.160986

36. de Medeiros P, Pinto DV, de Almeida JZ, et al. Modulation of Intestinal Immune and Barrier Functions by Vitamin A: Implications for Current Understanding of Malnutrition and Enteric Infections in Children. *Nutrients*. Aug 21 2018;10(9)doi:10.3390/nu10091128

37. Wapnir RA. Zinc deficiency, malnutrition and the gastrointestinal tract. *J Nutr*. May 2000;130(5S Suppl):1388S-92S. doi:10.1093/jn/130.5.1388S

38. Esmaillzadeh A, Azadbakht L. Legume consumption is inversely associated with serum concentrations of adhesion molecules and inflammatory biomarkers among Iranian women. *J Nutr*. Feb 2012;142(2):334-9. doi:10.3945/jn.111.146167

39. Abdel-Latif M, El-Shahawi G, Aboelhadid SM, Abdel-Tawab H. Modulation of murine intestinal immunity by Moringa oleifera extract in experimental hymenolepiasis nana. *J Helminthol*. Mar 2018;92(2):142-153. doi:10.1017/S0022149X17000293

40. Chagwena DT, Fernando S, Tavengwa NV, et al. Formulation and acceptability of local nutrient-dense foods for young children: A formative study for the Child Health Agriculture Integrated Nutrition (CHAIN) Trial in rural Zimbabwe. *Maternal & Child Nutrition: Under Press*.

41. Conway JM, Ingwersen LA, Moshfegh AJ. Accuracy of dietary recall using the USDA five-step multiple-pass method in men: an observational validation study. *Journal of the American Dietetic Association*. Apr 2004;104(4):595-603. doi:10.1016/j.jada.2004.01.007

42. Schakel SF. Maintaining a nutrient database in a changing marketplace: keeping pace with changing food products—a research perspective. Elsevier; 2001. p. 315-322.

43. Charrondiere U, Haytowitz D, Stadlmayr B. FAO/INFOODS density database, version 2.0. 2012:

44. US Department of Agriculture ARS, Nutrient Data Laboratory. USDA National Nutrient Database for Standard Reference, Release 27 (revised). http://www.ars.usda.gov/ba/bhncr/ndl

45. Merchant AT, Dehghan M, Chifamba J, Terera G, Yusuf S. Nutrient estimation from an FFQ developed for a black Zimbabwean population. *Nutrition Journal*. 2005/12/13 2005;4(1):37. doi:10.1186/1475-2891-4-37

46. Brown K, Dewey K, Allen L. Complementary feeding of young children in developing countries: a review of current scientific knowledge. 1998;

47. University UN, Organization WH. *Human Energy Requirements: Report of a Joint FAO/WHO/UNU Expert Consultation: Rome, 17-24 October 2001*. Food & Agriculture Org.; 2004.

48. Butte NF. Energy requirement of infants. *Public Health Nutrition*. 2009 2009;8(7A):953-967. In File.

49. WHO J. Protein and amino acid requirements in human nutrition. *World Health Organization technical report series*. 2007;(935):1.

50. Uauy R, Castillo C. Lipid requirements of infants: implications for nutrient composition of fortified complementary foods. *The Journal of nutrition*. 2003;133(9):2962S-2972S.

For peer review only

51. Micronutrients IoMPo. *Dietary Reference Intakes for Energy, Carbohydrate, Fiber, Fat, Fatty Acids, Cholesterol, Protein and Amino Acids. Panel on Macronutrients Panel on the Definition of Dietary Fiber, Subcommittee on Upper Reference Levels of Nutrients, Subcommittee on Interpretation and Uses of Dietary Reference Intakes, and the Standing Committee on the Scientific Evaluation of Dietary Reference Intakes, Food and Nutrition Board*. National Academies Press; 2005.
52. Dewey KG, Brown KH. Update on technical issues concerning complementary feeding of young children in developing countries and implications for intervention programs. *FOOD AND NUTRITION BULLETIN-UNITED NATIONS UNIVERSITY-*. 2003;24(1):5-28.
53. Callaghan M, Oyama M, Manary M. Sufficient protein quality of food aid varies with the physiologic status of recipients. *The Journal of nutrition*. 2017;147(3):277-280.
54. Intakes UD. SAS Macros for the NCI Method. 2012.
55. Tooze JA, Kipnis V, Buckman DW, et al. A mixed-effects model approach for estimating the distribution of usual intake of nutrients: the NCI method. *Statistics in medicine*. 2010;29(27):2857-2868.
56. Rytter MJH, Kolte L, Briend A, Friis H, Christensen VB. The immune system in children with malnutrition—a systematic review. *PloS one*. 2014;9(8):e105017.
57. Bourke CD, Jones KD, Prendergast AJ. Current understanding of innate immune cell dysfunction in childhood undernutrition. *Frontiers in immunology*. 2019;10:1728.
58. Whiley L, Nye LC, Grant I, et al. Ultrahigh-Performance Liquid Chromatography Tandem Mass Spectrometry with Electrospray Ionization Quantification of Tryptophan Metabolites and Markers of Gut Health in Serum and Plasma-Application to Clinical and Epidemiology Cohorts. *Anal Chem*. Apr 16 2019;91(8):5207-5216. doi:10.1021/acs.analchem.8b05884
59. Subramanian S, Huq S, Yatsunenko T, et al. Persistent gut microbiota immaturity in malnourished Bangladeshi children. *Nature*. 2014;510(7505):417-421.
60. Amoah AS, Obeng BB, Larbi IA, et al. Peanut-specific IgE antibodies in asymptomatic Ghanaian children possibly caused by carbohydrate determinant cross-reactivity. *J Allergy Clin Immunol*. Sep 2013;132(3):639-647. doi:10.1016/j.jaci.2013.04.023
61. Wollmann E, Hamsten C, Sibanda E, et al. Natural clinical tolerance to peanut in African patients is caused by poor allergenic activity of peanut IgE. *Allergy*. Jun 2015;70(6):638-52. doi:10.1111/all.12592

For peer review only

Table 1. Nutrient provision in IYCF and IYCF-plus trial arms

Figure 1. Nutrient requirements from complementary food and provided by IYCF and IYCF-PLUS intervention diets with and without estimated mealie meal consumption for three age groups of children.
Red (fails to meet requirement), orange (meets >88% requirement), green (exceeds requirement).[1]

	6-8 mo			9-11 mo			12-24 mo		
**Nutrient**	**Required from Complementary Food**	**IYCF**	**IYCF-PLUS**	**Required from Complementary Food**	**IYCF**	**IYCF-PLUS**	**Required from Complementary Food**	**IYCF**	**IYCF-PLUS**
Energy (kcal) from supplement alone	270	118	222	451	118	247	746	118	272
Energy (kcal) from supplement + estimated mealie meal intake		243	347		368	497		618	772
Protein (g)	7.7	2.6	10.8	8.1	2.6	12.6	8.1	2.6	14.4
Fat (g)	0.8	9.6	15.7	7.6	9.6	15.8	18.0	9.6	16.0
Vitamin A (µg RE)	207	400	519	222	400	540	238	400	560
Folic Acid (µg)	48	80	132	50	80	160	133	80	188
Calcium (mg)	232	280	375	246	280	438	360	280	502
Iron (mg)	9.2	6.0	9.0	9.2	6.0	10.4	5.7	6.0	11.9
Zinc (mg)	3.8	8.0	9.1	3.9	8.0	9.5	3.8	8.0	9.8
Choline (mg)	47	13	145	55	13	152	114	13	158

[1]Nutrient requirements from complementary food calculated as total requirement less provided in breastmilk. Estimated mealie meal consumption is 125 kcal, 250 kcal, and 500 kcal based on observed intakes during SHINE trial. IYCF diet includes SHINE IYCF behavior change modules + 20 g Nutributter per day. IYCF-PLUS diet includes IYCF behaviour change modules + 20 g Nutributter per day + whole egg powder, moringa powder, and iron and zinc-fortified sugar bean powder.

For peer review only

For peer review only - http://bmjopen.bmj.com/site/about/guidelines.xhtml

Table 2 – Trial outcomes at 9 months of age (window 9-11 months)

Endpoint	Definition
Energy intake	Percentage of infants meeting daily energy requirements at 9 months of age (window 9-11 months), measured by multi-pass 24-hour dietary recall.
Protein, iron, zinc and folate intake	Percentage of infants meeting daily protein, iron, zinc and folate requirements at 9 months of age, measured by multi-pass 24-hour dietary recall.
Length-for-age Z-score	Length-for-age expressed as a Z-score compared to the WHO 2006 reference median
Weight-for-age Z score	Weight-for-age expressed as a Z-score compared to the WHO 2006 reference median
Weight-for-length Z score	Weight-for-length expressed as a Z-score compared to the WHO 2006 reference median
Haemoglobin	Concentration of haemoglobin (in g/dL) in a whole blood sample, measured by HemoCue point-of-care assay and adjusted for altitude
Microbiome maturity	Microbiota-for-age Z-score
Environmental enteric dysfunction (EED)	Biomarkers of intestinal inflammation (faecal neopterin and myeloperoxidase), small intestinal damage (plasma I-FABP and citrulline), intestinal permeability (faecal A1AT), microbial translocation (plasma sCD14, LBP), systemic inflammation (plasma CRP, AGP, TNF-alpha and K:T ratio) and growth hormone axis (IGF-1)
Innate immune cell phenotype	Surface marker expression by peripheral blood monocytes and neutrophils
Innate immune cell function	Surface marker expression and cytokine secretion from innate immune cells challenged with lipopolysaccharide *in vitro* relative to unstimulated controls<br><br>Capacity of innate immune cells to internalise bacteria *in vitro*
Plasma essential amino acids	Plasma concentrations of phenylalanine, valine, threonine, tryptophan, methionine, leucine, isoleucine, lysine and histidine, as measured by LC-MS-MS
Plasma choline	Plasma concentration of choline, as measured by LC-MS-MS
Urinary metabolic signature	Global untargeted metabolomic phenotyping undertaken by ^{1}H nuclear magnetic resonance spectroscopy

For peer review only - http://bmjopen.bmj.com/site/about/guidelines.xhtml

Table 3 – CHAIN trial schedule[a]

**Procedure**	**Visit 1[b] Screening and enrolment**	**Randomisation and start of monthly IYCF or IYCF-plus interventions**	**Visit 2 Baseline**	**Visit 3[c] Endline**
Screening and eligibility check *Form 2*	x			
Informed consent *Form 3 and 4*	x			
Locator and contact information *Form 5*	x		x	x
Baseline interview *Form 6*			x	
Maternal weight, height and MUAC			x	
Infant weight, height, MUAC and head circumference			x	x
Infant blood collection[d]			x	x
Infant haemoglobin			x	x
Infant stool collection[c]			x	x
Infant urine collection[d]			x	x
Endline interview *Form 9*				x
24-hour dietary recall[e] *Form 10*				x
Informed consent for qualitative sub-studies [f] *Form 11*				
Qualitative sub-study 1 guide *Form 12a*				x
Informed consent for qualitative sub studies *Form 11* [g]				
Qualitative sub-study 2 guide *Form 12b*				x

[a]Trial enrolment began April, 2021 and concluded in August, 2021.

[b]For mothers who wish to provide informed consent on a subsequent day to screening, these visits will be separated.

[c]Target date 9 months of infant age (visit window 9-11 months or 274-334 days)

[d]If any specimens cannot be collected during the visit (e.g. if the infant fails to pass stool), the specimen collection will be rescheduled for the next day, or as soon as possible after the visit.

For peer review only

Rarely it may be necessary to repeat a specimen collection if the sample was insufficient, or fails quality control checks during processing in the laboratory. All blood draws will be kept to a safe limit, defined as maximum of 1ml/kg body weight.

[e]The primary outcome, measured by multi-pass 24-hour dietary recall, will be repeated 1 week later in a sub-sample of 50% infants for methods validation.

[f]Sub-group of up to 20 purposively selected households. The social scientist will visit the family during a separate visit at 7-9 months (214-273 days) of infant age, and obtain separate written informed consent for the qualitative interviews.

[g]Sub-group of up to 30 purposively selected households. The social scientist will obtain written informed consent for the qualitative interviews regarding migration, which will be conducted throughout the course of the study.

For peer review only

For peer review only - http://bmjopen.bmj.com/site/about/guidelines.xhtml

Table 4 – Module delivery

Infant age (window period)[a]	Home Visit	Infant age (window period)[a]	CONTROL ARM<br>IYCF modules and supplies	INTERVENTION ARM<br>IYCF-plus modules and supplies
5 months (5 months- <6 months)	1	5 months (5 months- <6 months) (153 – 182 days)	MODULE 1<br>• Nutrition for your baby<br>• Composition and functions of SQ-LNS [Feeding as a chore or actual work; stomach capacity and graph on nutrient gap of breastmilk and mealie meal]	MODULE 1<br>• Nutrition for your baby + introduction of the 3 powders<br>• Composition and functions of SQ-LNS and the 3 powders [Feeding as chore or actual work; stomach capacity and graph on nutrient gap of breastmilk and mealie meal)
6 months (6 months- <7 months)	2	6 months (6 months-<7 months) (183 – 213 days)	MODULE 2<br>• Introducing solid foods<br>• Additional information on SQ-LNS<br>• Breastmilk + porridge + SQ-LNS	MODULE 2<br>• Introducing solid foods<br>• Additional information on SQ-LNS and the 3 foods *(Bean powder, Egg powder and Moringa powder)*<br>• Breastmilk + porridge + SQ-LNS
6 months 1 week (6 months 1 week- <7 months)	3	6 months 1 week (6 months 1 week- <7 months) (190 - 213 days)	MODULE 2.1<br>• Frequency of complementary foods<br>• Reinforce messages<br>• Breastmilk + porridge + SQ-LNS	MODULE 2.1<br>• Introduce Bean Powder<br>• Frequency of complementary foods<br>• Reinforce messages<br>• Breastmilk + porridge + SQ-LNS + bean powder
6 months 2 weeks (6 months 2 weeks- <7 months)	4	6 months 2 weeks (6 months 2 weeks- <7 months) (197 – 213 days)	MODULE 2.2<br>Nutrient-dense complementary meals<br>Reinforce messages<br>Breastmilk + porridge+ SQ-LNS + nutrient density	MODULE 2.2<br>Introduce Egg Powder<br>Nutrient-dense complementary meals<br>Reinforce messages<br>Breastmilk +porridge+ SQ-LNS + bean powder + egg powder
6 months 3 weeks (6 months 3 weeks- <7 months)	5	6 months 3 weeks (6 months 3 weeks- <7 months) (204 – 213 days)	MODULE 2.3<br>• Complementary feeding schedule and family support<br>• Reinforce messages<br>• Breastmilk + porridge + SQ-LNS	MODULE 2.3<br>• Introduce Moringa<br>• Complementary feeding schedule and family support<br>• Reinforce messages<br>• Breastmilk + porridge + SQ-LNS + bean powder + egg powder + moringa powder
7 months (7 months- <8 months)	6	7 months (7 months- <8 months) (214 – 243 days)	MODULE 3<br>• Introducing more foods<br>• Reinforce messages<br>• Breastmilk + SQ-LNS + porridge (sadza/soup/potatoes/maheu+ other locally available foods e.g. vegetables etc.)	MODULE 3<br>• Introducing more foods<br>• Reinforce messages<br>• Breastmilk + SQ-LNS + bean powder + egg powder + moringa powder + porridge<br>(sadza /soup/potatoes/maheu + other locally available foods e.g. vegetables etc.)
8 months (8 months- <9 months)	7	8 months (8 months- <9 months) (244 – 273 days)	MODULE 4<br>• Feeding during illness<br>• Reinforce messages	MODULE 4<br>• Feeding during illness<br>• Reinforce messages

41

			• Breastmilk + SQ-LNS + porridge (sadza/soup/potatoes/maheu+ other locally available foods e.g. vegetables etc.)	• Breastmilk + SQ-LNS + bean powder + egg powder + moringa powder + porridge (sadza /soup/potatoes/maheu + other locally available foods e.g. vegetables etc.)
9 months (9 months- <10 months)	8	9 months (9 months- <10 months) (274 – 304 days)	MODULE 5<br>• Dietary Diversity<br>• Reinforce messages<br>• Dietary diversity<br>• Breastmilk + SQ-LNS + porridge (sadza/soup/potatoes/maheu+ other locally available foods e.g. vegetables etc.)	MODULE 5<br>• Dietary Diversity<br>• Reinforce messages<br>• Dietary diversity<br>• Breastmilk + SQ-LNS + bean powder + egg powder + moringa powder + porridge<br>(sadza /soup/potatoes/maheu + other locally available foods e.g. vegetables etc.)
10 months (10 months- <11 months)	9	10 months (10 months- <11 months) (305 – 334 days)	MODULE 6<br>• Reinforcing all Modules<br>• Breastmilk + SQ-LNS + porridge (sadza/soup/potatoes/maheu+ other locally available foods e.g. vegetables etc.)	MODULE 6<br>• Reinforcing all Modules<br>• Breastmilk + SQ-LNS + bean powder + egg powder + moringa powder + porridge<br>(sadza /soup/potatoes/maheu + other locally available foods e.g. vegetables etc.)
11 months (11 months- <12 months)	10	11 months (11 months- <12 months) (345 – 365 days)	MODULE 6<br>• Reinforcing all Modules<br>• Breastmilk + SQ-LNS + porridge (sadza/soup/potatoes/maheu+ other locally available foods e.g. vegetables etc.)	MODULE 6<br>• Reinforcing all Modules<br>• Breastmilk + SQ-LNS + bean powder + egg powder + moringa powder + porridge<br>(sadza /soup/potatoes/maheu + other locally available foods e.g. vegetables etc.)

[a] Each module session will be delivered for approximately 60 minutes. If a module is not delivered within the intervention window (i.e. appropriate infant age), the CHW will try to catch up by scheduling a new date as soon as possible. Each module will be delivered to the mother and her family. If the rescheduled module for modules 1.0 and 2.0 overlap, these two modules will be delivered at the same time. If the rescheduled module overlaps with the next visit for other modules (2.0, 2.1, 2.2, 2.3 etc) the visits will be scheduled at least 3 days apart so that families have time to absorb the new material. Delivery of IYCF-plus modules has therefore been designed to be flexible following complementary feeding guidance. Experience from formative work showed that it is feasible to deliver the combined modules at once.

For peer review only - http://bmjopen.bmj.com/site/about/guidelines.xhtml

Table 5: Quantities of food supplements in the IYCF-plus arm

Infant Age Group (months)	Mealie Meal (PVA maize)[1]	SQ-LNS[2]	Whole egg powder[3]	Moringa leaf, dried and ground[4]	Sugar bean legume, finely ground[5]
**6-8**	≥ 42 g (3 Tbsp)	20 g	14 g (3 tsp)	5 g (1 tsp)	5 g (1 tsp)
**9-11**	≥ 71 g (4.5 Tbsp)	20 g	14 g (3 tsp)	10 g (2 tsp)	10 g (2 tsp)

Food supplements will be delivered monthly by CHWs. [1]Mealie Meal (PVA maize) will be provided in 500g bags, with 3 bags/month (1500g) between 6-8 months of age and 5 bags/month (2500g) between 9-11 months of age. Households in the IYCF arm will receive the same amount of mealie meal per month [2]SQ-LNS will be supplied monthly to ensure 1 x 20g sachet per day can be provided (30 or 31 sachets per month). [3]Whole egg powder will be delivered as a 500g bag per month. [4]Moringa leaf powder will be supplied in 175g bags, with 1 bag/month (175g) between 6-8 months of age and 2 bags/month (350g) between 9-11 months of age. [5]NUA 45 sugar bean powder will be supplied in 175g bags, with 1 bag/month (175g) between 6-8 months of age and 2 bags/month (375g) between 9-11 months of age. These quantities allow for 15% extra in case of spillage or sharing

For peer review only - http://bmjopen.bmj.com/site/about/guidelines.xhtml

Table 6 – Laboratory analyses

Sample type	Assay	Method	Location of work	Study subjects	Time-points
Plasma	I-FABP, CRP, AGP, TNFα, LBP, sCD14, IGF-1	ELISA	Zvitambo	All	Baseline, endline
Peripheral blood leukocytes	Innate immune cell phenotype[a]	Flow cytometry	Zvitambo	All	Baseline, endline
Whole blood	Whole Blood Culture with and without LPS	Cell culture, flow cytometry and ELISA	Zvitambo	All	Baseline, endline
Whole blood	Bacterial binding Assay	Cell culture, flow cytometry	Zvitambo	All	Baseline, endline
Stool	Myeloperoxidase, neopterin, alpha-1 antitrypsin	ELISA	Zvitambo	All	Baseline, endline
Urine	Global untargeted metabolomic phenotyping	^{1}H NMR spectroscopy	Southampton, UK	All	Baseline, endline
Plasma	Global untargeted metabolomic phenotyping	^{1}H NMR spectroscopy	Southampton, UK	All	Baseline, endline
Plasma	Kynurenine:tryptophan ratio, citrulline, essential amino acids, choline	Ultrahigh-performance liquid chromatography tandem mass spectrometry with electrospray ionization	Southampton, UK	All	Baseline, endline
Stool	Whole metagenome shotgun sequencing	Illumina HiSeq	Blizard Institute, UK	All	Baseline, endline
Stool	Metabolic phenotyping of fecal water	^{1}H NMR spectroscopy	Southampton, UK	All	Baseline, endline
Urine	Aflatoxin M1 and creatinine	ELISA	Zvitambo	All	Baseline, endline

[a] Expression of surface activation markers HLA-DR, CD64 and CD16 on monocytes and HLA-DR, CD64, CD16 and CD62L on neutrophils

I-FABP: Intestinal fatty acid binding protein; AGP: Alpha-1 acid glycoprotein; CRP: C-reactive protein; LBP: lipopolysaccharide binding protein; IGF-1: insulin like growth factor 1

For peer review only - http://bmjopen.bmj.com/site/about/guidelines.xhtml

List of Figures

Figure 1 – Hypothesized impact of IYCF-plus intervention on barriers to nutrient intake, uptake and utilization for healthy child growth

For peer review only

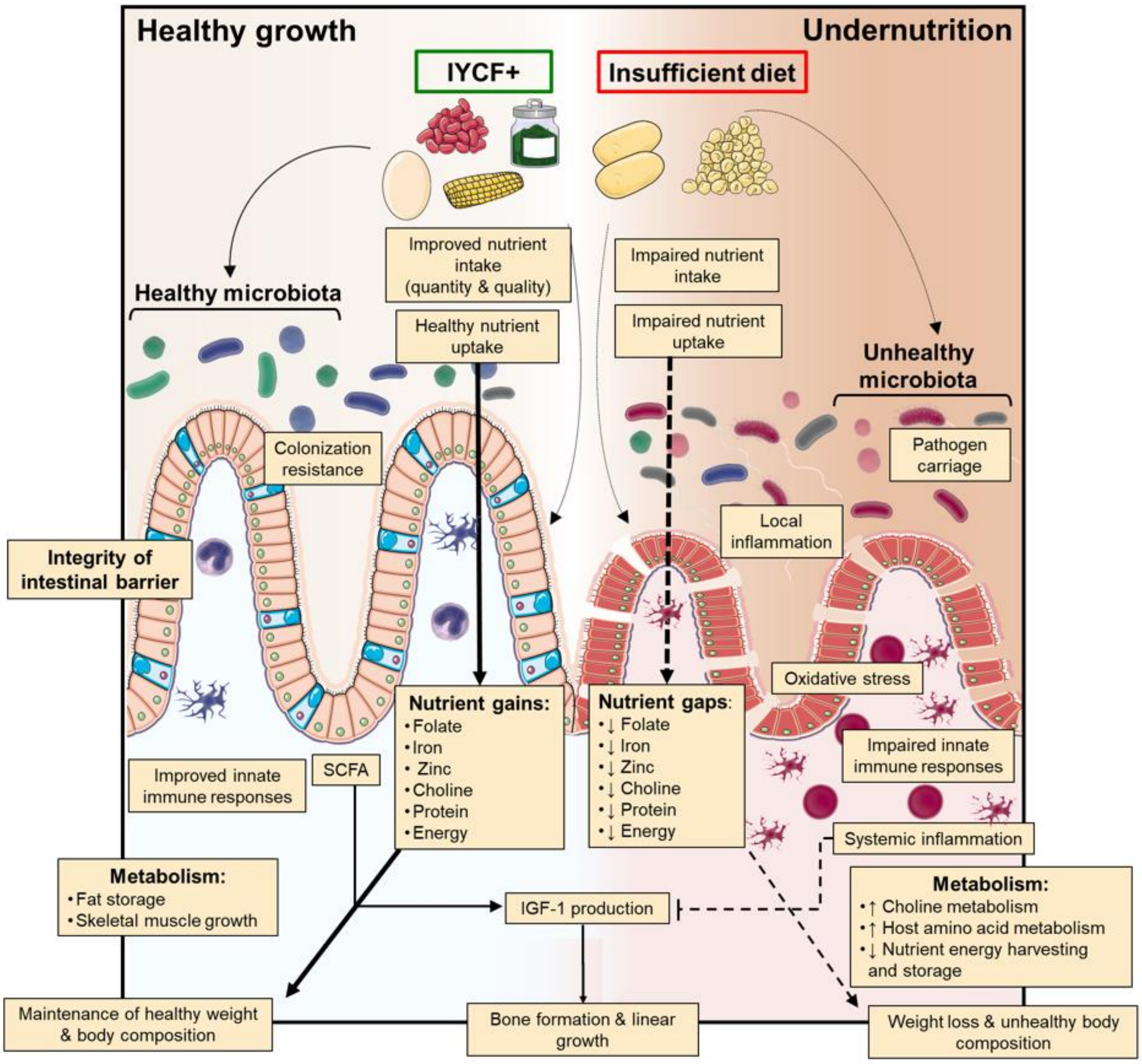

For peer review only - http://bmjopen.bmj.com/site/about/guidelines.xhtml

SPIRIT

STANDARD PROTOCOL ITEMS: RECOMMENDATIONS FOR INTERVENTIONAL TRIALS

SPIRIT 2013 Checklist: Recommended items to address in a clinical trial protocol and related documents*

Section/item	Item No	Description	CHAIN
**Administrative information**			
Title	1	Descriptive title identifying the study design, population, interventions, and, if applicable, trial acronym	1
Trial registration	2a	Trial identifier and registry name. If not yet registered, name of intended registry	3
	2b	All items from the World Health Organization Trial Registration Data Set	3
Protocol version	3	Date and version identifier	3
Funding	4	Sources and types of financial, material, and other support	x
Roles and responsibilities	5a	Names, affiliations, and roles of protocol contributors	1
	5b	Name and contact information for the trial sponsor	31
	5c	Role of study sponsor and funders, if any, in study design; collection, management, analysis, and interpretation of data; writing of the report; and the decision to submit the report for publication, including whether they will have ultimate authority over any of these activities	31

	5d	Composition, roles, and responsibilities of the coordinating centre, steering committee, endpoint adjudication committee, data management team, and other individuals or groups overseeing the trial, if applicable (see Item 21a for data monitoring committee)	31
**Introduction**			
Background and rationale	6a	Description of research question and justification for undertaking the trial, including summary of relevant studies (published and unpublished) examining benefits and harms for each intervention	4
	6b	Explanation for choice of comparators	5
Objectives	7	Specific objectives or hypotheses	6
Trial design	8	Description of trial design including type of trial (eg, parallel group, crossover, factorial, single group), allocation ratio, and framework (eg, superiority, equivalence, noninferiority, exploratory)	6
**Methods: Participants, interventions, and outcomes**			
Study setting	9	Description of study settings (eg, community clinic, academic hospital) and list of countries where data will be collected. Reference to where list of study sites can be obtained	12
Eligibility criteria	10	Inclusion and exclusion criteria for participants. If applicable, eligibility criteria for study centres and individuals who will perform the interventions (eg, surgeons, psychotherapists)	13

SAMPLE
For peer review only

Interventions	11a	Interventions for each group with sufficient detail to allow replication, including how and when they will be administered	15
	11b	Criteria for discontinuing or modifying allocated interventions for a given trial participant (eg, drug dose change in response to harms, participant request, or improving/worsening disease)	27
	11c	Strategies to improve adherence to intervention protocols, and any procedures for monitoring adherence (eg, drug tablet return, laboratory tests)	26
	11d	Relevant concomitant care and interventions that are permitted or prohibited during the trial	27
Outcomes	12	Primary, secondary, and other outcomes, including the specific measurement variable (eg, systolic blood pressure), analysis metric (eg, change from baseline, final value, time to event), method of aggregation (eg, median, proportion), and time point for each outcome. Explanation of the clinical relevance of chosen efficacy and harm outcomes is strongly recommended	22, 38
Participant timeline	13	Time schedule of enrolment, interventions (including any run-ins and washouts), assessments, and visits for participants. A schematic diagram is highly recommended (see Figure)	41

Sample size	14	Estimated number of participants needed to achieve study objectives and how it was determined, including clinical and statistical assumptions supporting any sample size calculations	22
Recruitment	15	Strategies for achieving adequate participant enrolment to reach target sample size	12
**Methods: Assignment of interventions (for controlled trials)**			
Allocation:			
Sequence generation	16a	Method of generating the allocation sequence (eg, computer-generated random numbers), and list of any factors for stratification. To reduce predictability of a random sequence, details of any planned restriction (eg, blocking) should be provided in a separate document that is unavailable to those who enrol participants or assign interventions	15
Allocation concealment mechanism	16b	Mechanism of implementing the allocation sequence (eg, central telephone; sequentially numbered, opaque, sealed envelopes), describing any steps to conceal the sequence until interventions are assigned	15
Implementation	16c	Who will generate the allocation sequence, who will enrol participants, and who will assign participants to interventions	15
Blinding (masking)	17a	Who will be blinded after assignment to interventions (eg, trial participants, care providers, outcome assessors, data analysts), and how	15

For peer review only - http://bmjopen.bmj.com/site/about/guidelines.xhtml

	17b	If blinded, circumstances under which unblinding is permissible, and procedure for revealing a participant's allocated intervention during the trial	15
**Methods: Data collection, management, and analysis**			
Data collection methods	18a	Plans for assessment and collection of outcome, baseline, and other trial data, including any related processes to promote data quality (eg, duplicate measurements, training of assessors) and a description of study instruments (eg, questionnaires, laboratory tests) along with their reliability and validity, if known. Reference to where data collection forms can be found, if not in the protocol	14
	18b	Plans to promote participant retention and complete follow-up, including list of any outcome data to be collected for participants who discontinue or deviate from intervention protocols	17
Data management	19	Plans for data entry, coding, security, and storage, including any related processes to promote data quality (eg, double data entry; range checks for data values). Reference to where details of data management procedures can be found, if not in the protocol	20
Statistical methods	20a	Statistical methods for analysing primary and secondary outcomes. Reference to where other details of the statistical analysis plan can be found, if not in the protocol	22
	20b	Methods for any additional analyses (eg, subgroup and adjusted analyses)	22

For peer review only - http://bmjopen.bmj.com/site/about/guidelines.xhtml

	20c	Definition of analysis population relating to protocol non-adherence (eg, as randomised analysis), and any statistical methods to handle missing data (eg, multiple imputation)	22
**Methods: Monitoring**			
Data monitoring	21a	Composition of data monitoring committee (DMC); summary of its role and reporting structure; statement of whether it is independent from the sponsor and competing interests; and reference to where further details about its charter can be found, if not in the protocol. Alternatively, an explanation of why a DMC is not needed	28
	21b	Description of any interim analyses and stopping guidelines, including who will have access to these interim results and make the final decision to terminate the trial	28
Harms	22	Plans for collecting, assessing, reporting, and managing solicited and spontaneously reported adverse events and other unintended effects of trial interventions or trial conduct	27
Auditing	23	Frequency and procedures for auditing trial conduct, if any, and whether the process will be independent from investigators and the sponsor	28
**Ethics and dissemination**			
Research ethics approval	24	Plans for seeking research ethics committee/institutional review board (REC/IRB) approval	28

Protocol amendments	25	Plans for communicating important protocol modifications (eg, changes to eligibility criteria, outcomes, analyses) to relevant parties (eg, investigators, REC/IRBs, trial participants, trial registries, journals, regulators)	28
Consent or assent	26a	Who will obtain informed consent or assent from potential trial participants or authorised surrogates, and how (see Item 32)	29
	26b	Additional consent provisions for collection and use of participant data and biological specimens in ancillary studies, if applicable	29
Confidentiality	27	How personal information about potential and enrolled participants will be collected, shared, and maintained in order to protect confidentiality before, during, and after the trial	29
Declaration of interests	28	Financial and other competing interests for principal investigators for the overall trial and each study site	32
Access to data	29	Statement of who will have access to the final trial dataset, and disclosure of contractual agreements that limit such access for investigators	21
Ancillary and post-trial care	30	Provisions, if any, for ancillary and post-trial care, and for compensation to those who suffer harm from trial participation	28

For peer review only - http://bmjopen.bmj.com/site/about/guidelines.xhtml

Dissemination policy	31a	Plans for investigators and sponsor to communicate trial results to participants, healthcare professionals, the public, and other relevant groups (eg, via publication, reporting in results databases, or other data sharing arrangements), including any publication restrictions	29
	31b	Authorship eligibility guidelines and any intended use of professional writers	Not included in this manuscript
	31c	Plans, if any, for granting public access to the full protocol, participant-level dataset, and statistical code	29
**Appendices**			
Informed consent materials	32	Model consent form and other related documentation given to participants and authorised surrogates	Available online at Open Science Framework (https://osf.io/njy2a/).
Biological specimens	33	Plans for collection, laboratory evaluation, and storage of biological specimens for genetic or molecular analysis in the current trial and for future use in ancillary studies, if applicable	23

*It is strongly recommended that this checklist be read in conjunction with the SPIRIT 2013 Explanation & Elaboration for important clarification on the items. Amendments to the protocol should be tracked and dated. The SPIRIT checklist is copyrighted by the SPIRIT Group under the Creative Commons "Attribution-NonCommercial-NoDerivs 3.0 Unported" license.

SAMPLE

For peer review only
